# Copper-catalyzed one-pot synthesis of isoquinolines *via* oxidative α-amination under mild and sustainable conditions

**DOI:** 10.1039/d6ra01207h

**Published:** 2026-04-24

**Authors:** Ali Akbari, Danial Zand Hoseinshahi

**Affiliations:** a Department of Chemistry, Faculty of Science, University of Jiroft Jiroft Iran P. O. Box 8767161167 a.akbari@ujiroft.ac.ir +98-344-334-7065 +98-344-334-7061

## Abstract

A copper-catalyzed multicomponent protocol is described for the synthesis of structurally diverse isoquinolines from readily available phenylacetaldehyde derivatives, benzaldehydes, and ammonia. A commercially available 4 M solution of ammonia in methanol serves as a practical and comparatively benign nitrogen source, avoiding the use of prefunctionalized amine substrates. The transformation proceeds at room temperature *via* a modified Pomeranz–Fritsch-type cyclization, enabling direct one-pot access to the target heterocycles in good to excellent yields under operationally simple conditions. The multicomponent design, combined with the avoidance of stoichiometric activating reagents and strongly acidic media, leads to improved atom economy and reduced waste generation relative to conventional isoquinoline syntheses. Mechanistic studies indicate a stepwise pathway involving the formation and isolation of a key 1,4-diphenyl-2-azabutadiene intermediate, while TEMPO trapping experiments support the involvement of radical species. Notably, a heterogeneous CuO/TiO_2_ catalyst can be readily recovered and reused with minimal loss of activity, further enhancing the sustainability profile of the methodology. Overall, this approach integrates earth-abundant copper catalysis, mild conditions, multicomponent efficiency, and mechanistic insight for the environmentally considerate construction of isoquinoline frameworks.

## Abstract 2

Copper-catalyzed multicomponent one-pot synthesis of isoquinolines *via* a modified Pomeranz–Fritsch-type cyclization.

## Graphical abstract

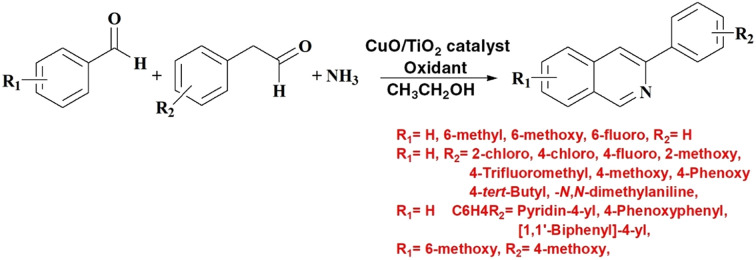

## Introduction

**Fig. 1:**
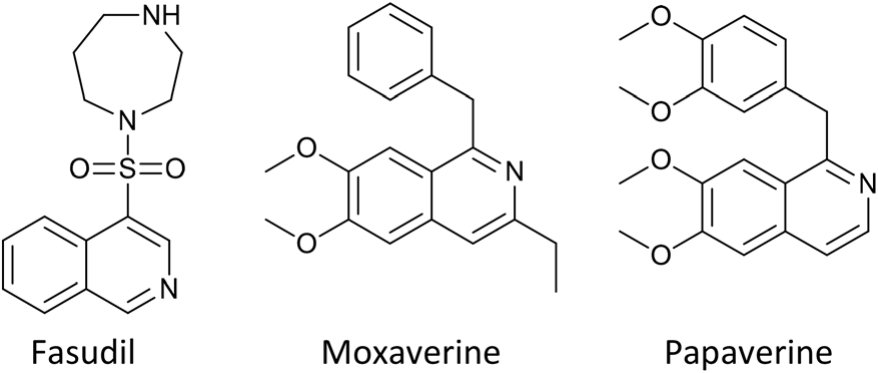
Known marketed drugs of the isoquinoline.

Isoquinolines constitute an important class of nitrogen-containing heterocycles that have attracted considerable attention due to their broad spectrum of biological activities. Isoquinoline-based compounds have been extensively reported to exhibit anticancer,^1,2^ antibacterial,^3,4^ antiviral,^5,6^ antitumor,^7^ antitubercular, antifungal,^8^ and antimalarial^9^ properties. Owing to this remarkable pharmacological versatility, the isoquinoline scaffold is frequently encountered as a core structural motif in numerous clinically approved and commercially important drugs, including Fasudil, Moxaverine, and Papaverine (Fig. 1).^10^

In recent years, a large number of structurally diverse isoquinoline compounds have been isolated from natural sources, particularly microorganisms and plants.^11^ Isoquinoline alkaloids exhibit a wide range of biological activities, including anticancer,^12^ antitumor,^13^ antifungal,^14^ antibacterial,^15^ antiviral,^16,17^ and broad-spectrum antimicrobial effects.^18,19^ Beyond their pharmaceutical relevance, isoquinoline derivatives—especially isoquinoline *N*-oxides—have attracted increasing attention as photoactive motifs and play important roles in photochemical transformations.^20^ Isoquinolines have also found extensive application as ligands in transition-metal-catalyzed reactions, notably those involving cobalt,^21^ ruthenium,^22,23^ and nickel^24^ complexes, further underscoring the versatility of this heterocyclic scaffold. Owing to these important attributes, considerable efforts have been devoted to the development of efficient synthetic methodologies for isoquinoline derivatives. Classical approaches include the Pomeranz–Fritsch reaction between benzaldehydes and 2,2-dialkoxyethylamines under acidic conditions,^25^ the Bischler–Napieralski cyclization of β-arylethylamides or carbamates,^26^ the Pictet–Spengler condensation of β-arylethylamines with aldehydes or ketones,^27^ and the Pictet–Gams cyclization of β-hydroxy-β-phenethylamides.^28^ Despite their historical significance, many of these traditional methods suffer from inherent drawbacks that contradict modern sustainability metrics. For example, the Pomeranz–Fritsch reaction typically requires strong aqueous acids (≈6 M) and elevated temperatures (>100 °C), which severely restrict substrate scope and result in high *E*-factors due to extensive neutralization and aqueous waste.^29^ Subsequent modifications employing excess Lewis's acids, such as aluminum trichloride,^30^ strong Brønsted acids such as trifluoroacetic acid, or transition-metal catalysis have been reported.^31,32^ Nevertheless, many existing approaches to isoquinoline synthesis continue to suffer from drawbacks such as harsh reaction conditions, the use of stoichiometric hazardous reagents, multiple isolation steps, and poor atom economy. Despite extensive efforts devoted to the construction of isoquinoline frameworks, a general, mild, and highly efficient method that minimizes environmental impact remains critically needed. This gap in current methodology prompted us to explore an alternative, milder approach to isoquinolines based on the α-amination of phenylacetaldehyde derivatives. Radical-mediated transformations have recently emerged as powerful tools for C–N bond formation, benefiting from mild reaction conditions, broad functional-group tolerance, and unique reactivity patterns, aligning closely with the principles of Green Chemistry.^33–36^ Although α-aminocarbonyl compounds are well established as versatile intermediates in radical chemistry, their application in the assembly of isoquinoline frameworks has remained largely unexplored.^37–40^ We propose that a copper-catalyzed, radical-driven α-amination strategy, utilizing an earth-abundant catalyst and aqueous ammonia, could provide an exceptionally efficient and environmentally benign access to isoquinoline derivatives from readily available phenylacetaldehyde precursors, while entirely circumventing the harsh conditions and multistep protocols associated with classical methods.

Compared with classical isoquinoline syntheses such as the Pomeranz–Fritsch, Bischler–Napieralski, and Pictet–Spengler reactions, the present methodology operates under markedly milder and more sustainable conditions (Table 1). The avoidance of strong mineral acids, stoichiometric dehydrating agents, and elevated temperatures, combined with a one pot multicomponent design and visible light activation, results in significantly reduced waste generation and improved atom efficiency.

**Table 1:** Optimization of the reaction conditions for the synthesis of 3-phenylisoquinoline (3a)^a^

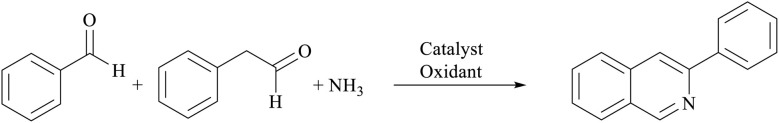								
1a–3a								
Entry	CuO/TiO_2_ (mg)	CuCl (mol%)	Ascorbic acid (mol%)	Solvent (10 mL)	Time (h)	Temp (°C)	Light	Yield (%)
1	—	—	—	H_2_O	24	r.t	Visible	0
2	—	—	35	H_2_O	24	r.t	Visible	0
3	—	8	—	H_2_O	24	r.t	Visible	Trace^b^
4	—	8	35	H_2_O	24	r.t	Visible	23
5	30	—	35	H_2_O	24	r.t	Visible	Trace
6	30	8	—	H_2_O	24	r.t	Visible	41
7	30	8	35	H_2_O	24	r.t	Dark	0
8	30	8	35	H_2_O	24	r.t	Visible	90
9	30	8	35	EtOH	24	r.t	Visible	92
10	30	8	35	bEtOH 95%	24	r.t	Visible	93
11	30	8	35	CH_2_Cl_2_	24	r.t	Visible	35
12	30	8	35	Toluene	24	r.t	Visible	42
13	30	8	35	DMF	24	r.t	Visible	75
14	TiO_2_ (30)	8	35	EtOH 95%	24	r.t	Visible	Trace
15	CuO (30)	8	35	EtOH 95%	24	r.t	Visible	37
16	20	8	35	EtOH 95%	24	r.t	Visible	93
17	20	8	45	EtOH 95%	24	r.t	Visible	93
18	20	8	25	EtOH 95%	24	r.t	Visible	93
19	20	8	20	EtOH 95%	24	r.t	Visible	83
20	20	8	15	EtOH 95%	24	r.t	Visible	72
21	20	8	20	EtOH 95%	14	35	Visible	90
22	20	8	20	EtOH 95%	14	35	Visible	90
23	20	8	25	EtOH 95%	14	50	Visible	82
24	20	8	25	EtOH 95%	14	70	Visible	75
25	20	8	25	EtOH 95%	14	r.t	Visible	93
26	20	8	25	EtOH 95%	14	r.t	LED c	86

a Reaction conditions: phenylacetaldehyde (1.0 mmol), benzaldehyde (1.02 mL, 10 mmol), NH_3_·MeOH (4 M, 0.25 mL), CuO/TiO_2_, CuCl, ascorbic acid, solvent (10 mL), visible-light irradiation using a 23 W household fluorescent lamp.

b The product was monitored with TLC.

c 10 W, 450 nm.

## Results and discussion

### Characterization of the catalyst

The CuO/TiO_2_ catalyst was prepared following previously reported procedures with slight modifications.^41,42^ In brief, the material was synthesized *via* an electrochemical method using a metallic copper electrode in a TiO_2_ sol–gel medium acting as the electrolyte. During the electrochemical process, copper(i) species were generated through the oxidation of metallic copper at the electrode–solution interface. The resulting Cu(i) species subsequently reacted with water to form CuOH, which upon dehydration yielded Cu_2_O nanostructures. Further oxidation of Cu_2_O on the electrode surface led to the formation of CuO nanostructures immobilized within the TiO_2_ matrix. The structural, morphological, and compositional properties of the synthesized CuO/TiO_2_ catalyst were systematically investigated using X-ray diffraction (XRD), Fourier-transform infrared spectroscopy (FT-IR), textural properties (N_2_ adsorption–desorption), scanning electron microscopy (SEM), and energy-dispersive X-ray spectroscopy (EDS). The electrochemical co-deposition approach enabled efficient incorporation of copper species into the TiO_2_ matrix, resulting in nanocomposites of uniform composition and morphology. As confirmed by energy-dispersive X-ray spectroscopy (EDS), the CuO/TiO_2_ nanocomposite contained approximately 5 wt% copper. This observation was further supported by inductively coupled plasma mass spectrometry (ICP-MS) analysis, which quantitatively determined a copper content of 5.0 wt%. The close agreement between the targeted and experimentally determined values verifies the controllability and reproducibility of the electrochemical synthesis parameters—specifically, the applied voltage (8–10 V) and anodization time (1 h)—for achieving precise copper loading. The consistency of both EDS and ICP-MS data also indicates that copper species are homogeneously distributed within the TiO_2_ framework rather than forming segregated aggregates, confirming the effectiveness of the electrochemical deposition strategy and its suitability for catalytic applications. X-ray diffraction (XRD) analysis further corroborated the formation of crystalline CuO domains embedded within the anatase TiO_2_ lattice, while FT-IR spectra displayed characteristic Cu–O stretching bands, confirming the chemical incorporation of copper oxide species into the composite structure.

## Results and discussion (revised with XRD analysis)

**Fig. 2:**
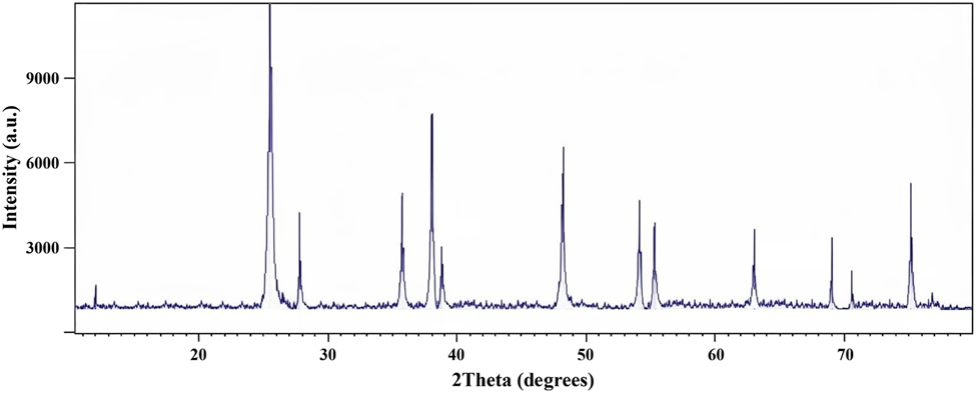
X-ray diffraction (XRD) pattern of the CuO/TiO_2_ composite, synthesized *via* a green chemistry method. Indexed to anatase TiO_2_ (JCPDS No. 21-1272) and monoclinic CuO (JCPDS No. 48-1548), confirming successful composite formation for potential applications in sustainable catalysis.

X-ray diffraction (XRD) analysis was employed to ascertain the crystalline structure and phase composition of the synthesized CuO/TiO_2_ composite. The resulting diffraction pattern (Fig. 2) confirms the presence of both anatase TiO_2_ and monoclinic CuO phases, consistent with established reference data (JCPDS No. 21 1272 for TiO_2_ and JCPDS No. 48 1548 for CuO). The absence of extraneous peaks indicates a high degree of phase purity. The prominent diffraction peaks at 2*θ* ≈ 25.3°, 37.8°, and 48.0° are assigned to the (101), (004), and (200) planes of anatase TiO_2_, respectively. Simultaneously, peaks at 2*θ* ≈ 32.5°, 35.5°, and 38.7° are attributed to the (110), (−111), and (111) planes of monoclinic CuO. These observations corroborate the successful formation and incorporation of CuO within the TiO_2_ support matrix.

Furthermore, the sharpness and well-defined nature of the diffraction peaks imply a high level of crystallinity and a homogeneous distribution of CuO nanoparticles on the TiO_2_ support. These characteristics are crucial for maximizing the surface area accessible for catalytic activity, reinforcing the potential of this composite material for sustainable chemical transformations. The absence of secondary phases further validates the controlled synthesis of the CuO/TiO_2_ composite. These findings strongly support the application of this material within Green Chemistry frameworks by providing a stable, highly crystalline, and catalytically active heterogeneous catalyst.

### FTIR analysis of synthesized material

**Fig. 3:**
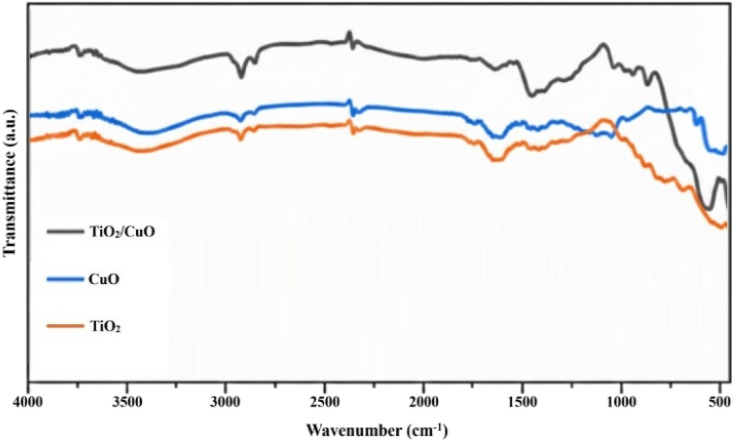
FTIR spectra of TiO_2_/CuO composite, CuO, and TiO_2_, confirming successful synthesis and revealing characteristic peaks indicative of each component and interfacial interactions.

The FT-IR spectra of TiO_2_, CuO, and the TiO_2_/CuO composite are illustrated in Fig. 3. The broad absorption band observed around 3000–3400 cm^−1^ is attributed to the O–H stretching vibrations of adsorbed water molecules on the surface of the photocatalysts. The characteristic peaks at approximately 1630 cm^−1^ and 1450 cm^−1^ correspond to the bending vibrations of O–H groups and the stretching of Ti–O bonds, respectively. In the lower wavenumber region, the distinct peak at 550 cm^−1^ is assigned to the Cu–O stretching mode, confirming the successful incorporation of CuO into the composite. For the TiO_2_/CuO hybrid, a combination of these characteristic vibrational modes is observed. A slight shift in the peak positions compared to the pure components suggests a strong interfacial interaction between TiO_2_ and CuO, which can facilitate charge carrier separation—a crucial factor for enhanced catalytic performance in green chemical processes.

**Fig. 4:**
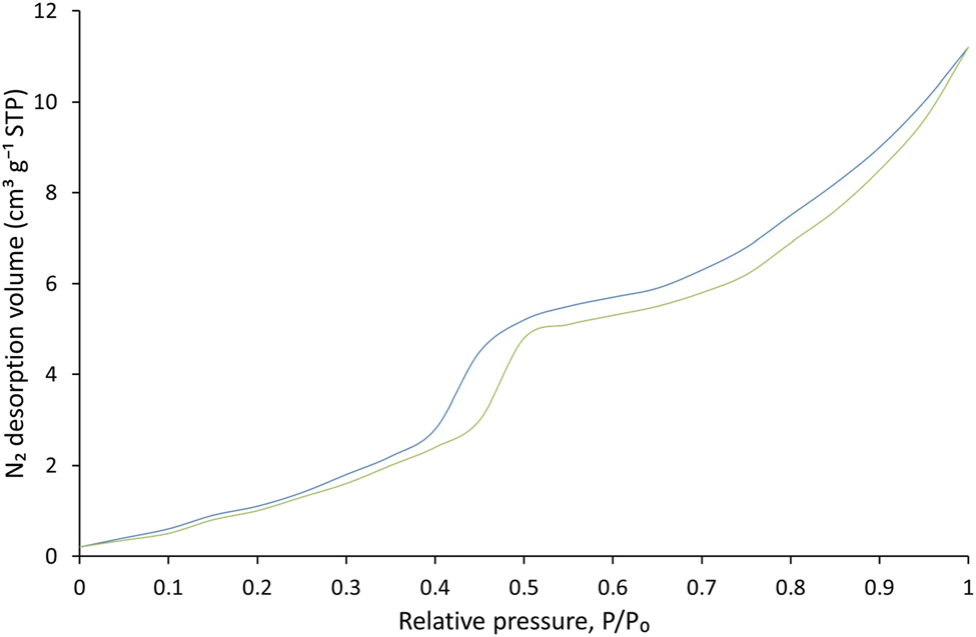
N_2_ adsorption–desorption isotherms of the synthesized material measured at 77 K, displaying a type IV isotherm with a hysteresis loop characteristic of mesoporous structures.

The nitrogen adsorption–desorption isotherm (Fig. 4) confirms the mesoporous nature of the material, displaying a type IV isotherm with a characteristic hysteresis loop indicative of pores between 2–50 nm, beneficial for enhanced mass transport and surface interactions. The specific surface area is approximately 25 m^2^ g^−1^, representing moderate porosity. The observed hysteresis loop suggests the presence of capillary-like pores, potentially arising from particle aggregation and aligning with expected SEM observations. Initial low adsorption at lower pressures indicates limited initial accessibility, with significant adsorption enhancement at higher pressures, signifying increased pore accessibility. Notably, the composite material (blue curve) exhibits a significantly larger surface area compared to the individual components (green curve), highlighting a synergistic effect that enhances surface reactivity. Overall, these data demonstrate a material with a favorable pore structure suitable for catalytic or adsorptive applications, with the composite demonstrating improved performance relative to its constituent parts.

### Morphological analysis (SEM)

**Fig. 5:**
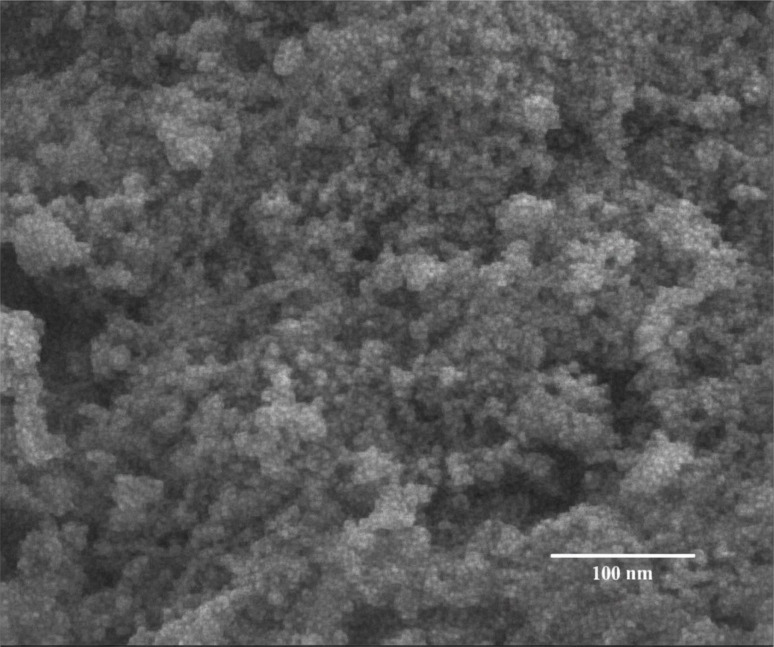
Scanning electron microscopy (SEM) image of the synthesized material showing a densely packed nanogranular morphology with interconnected nanoparticles. Scale bar: 100 nm.

The SEM image (Fig. 5) reveals a nanogranular morphology characterized by aggregated nanoparticles that form an interconnected porous network. These nanoparticles are uniformly distributed without the presence of large, well-defined crystals, resulting in a rough and porous surface texture.

This nanostructured architecture provides a high surface area to volume ratio, which is highly advantageous for catalytic and adsorption applications by enhancing surface reactivity and the accessibility of active sites. The morphology observed thus supports the material's potential for improved performance in catalytic processes due to the synergistic effects of nanoparticle aggregation and porosity.

### Elemental composition and distribution (EDS)

**Fig. 6:**
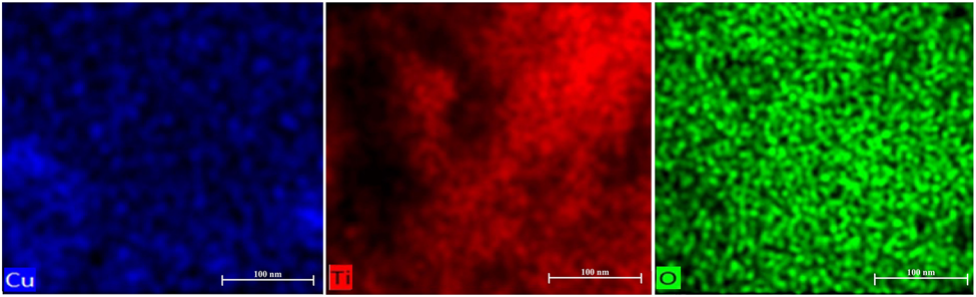
Energy-dispersive X-ray (EDS) elemental mapping images of the synthesized material showing the spatial distribution of Cu, Ti and O. The uniform distribution of Ti and O confirms the formation of a homogeneous TiO_2_ matrix, while the weak Cu signal originates from the copper grid used during SEM/EDS measurements. Scale bar: 100 nm.

Fig. 6 presents energy-dispersive X-ray (EDS) elemental mapping, revealing the spatial distribution of copper (Cu), titanium (Ti), and oxygen (O) within the synthesized material. The images demonstrate a homogeneous distribution of titanium and oxygen, supporting the formation of a consistent TiO_2_ matrix. The observed copper signal is minimal and localized to the surface, attributable to the copper grid used during SEM/EDS analysis, rather than representing significant copper incorporation into the material's structure. This uniformity is a desirable characteristic in heterogeneous catalysts, promoting consistent reactivity and minimizing potential mass transport limitations. The absence of significant copper distribution also aligns with Green Chemistry principles by minimizing the presence of potentially hazardous or non-active elements within the catalytic material.

**Fig. 7:**
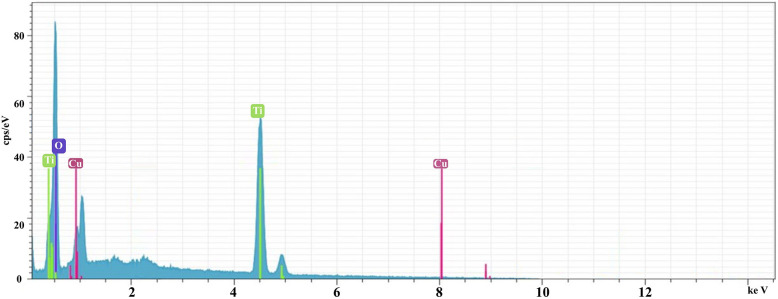
Energy-dispersive X-ray (EDS) spectrum of the synthesized material, confirming the presence of titanium (Ti) and oxygen (O) as the main components, alongside characteristic signals from copper (Cu). The weak Cu peaks are attributed to the copper grid used during SEM/EDS analysis.

The EDS spectrum reveals the presence of titanium (Ti), oxygen (O), and copper (Cu) within the analyzed sample. The prominent peaks at approximately 3.2 keV (Ti), 5.1 keV (O), and 7.9 keV (Cu) indicate the successful incorporation of these elements into the material (Fig. 7). The relative intensities of the peaks can be used to determine the elemental composition and stoichiometry of the sample. For example, the peak height for Ti is significantly higher than that of Cu, suggesting a higher concentration of titanium in the material. The presence of oxygen is expected due to the material's composition and potential oxidation states. This confirms the successful synthesis of Cu-decorated TiO_2_ with a well-defined composition, essential for consistent performance in catalytic and adsorptive processes. It should be noted that the EDS analysis was performed using a copper TEM grid; therefore, the observed Cu signal in the EDS mapping may partially originate from the grid and overlap with the signal from the catalyst. Consequently, the copper content of the catalyst was more accurately determined by ICP analysis, which confirmed a copper loading of approximately 5 wt%.

### Optimization of reaction conditions

We developed a copper-catalyzed, one-pot strategy that enables the direct synthesis of isoquinolines without intermediate isolation steps, affording the desired products in good to excellent yields under remarkably mild and operationally simple conditions. This transformation proceeds *via* oxidative homocoupling of phenylacetaldehyde derivatives to generate α-aminocarbonyl intermediates, followed by intramolecular cyclization to furnish isoquinolines. The protocol shows broad substrate generality and provides a complementary copper-catalyzed route to isoquinoline frameworks.

Notably, the key 1,4-diphenyl-2-azabutadiene intermediate could be readily isolated and fully characterized (see SI). Under the standard reaction conditions, this intermediate was smoothly converted into the corresponding isoquinoline through an oxidative rearrangement process, confirming its competence as a key precursor.

### 1,4-Diphenyl-2-azabutadiene

The key 1,4-diphenyl-2-azabutadiene intermediate was readily isolated. After completion of the reaction, the mixture was extracted with ethyl acetate (3 × 30 mL). The combined organic layers were dried over anhydrous MgSO_4_, filtered, and concentrated under reduced pressure to afford a solid residue. Purification by short-column chromatography on silica gel (Et_2_O as eluent), followed by recrystallization from hexane, furnished the desired compound as a white solid (mp 43–54 °C).

^1^H NMR (500 MHz, CDCl_3_) *δ* 8.40 (s, 1H), 7.85 (m, 2H), 7.25–7.65 (m, 9H), 7.05 (d, *J* = 13.0 Hz, 1H).

^13^C NMR (125 MHz, CDCl_3_) *δ* 161.3, 141.8, 136.2, 136.1, 131.1, 128.7, 128.6, 127.9, 126.8.

**Fig. 8:**
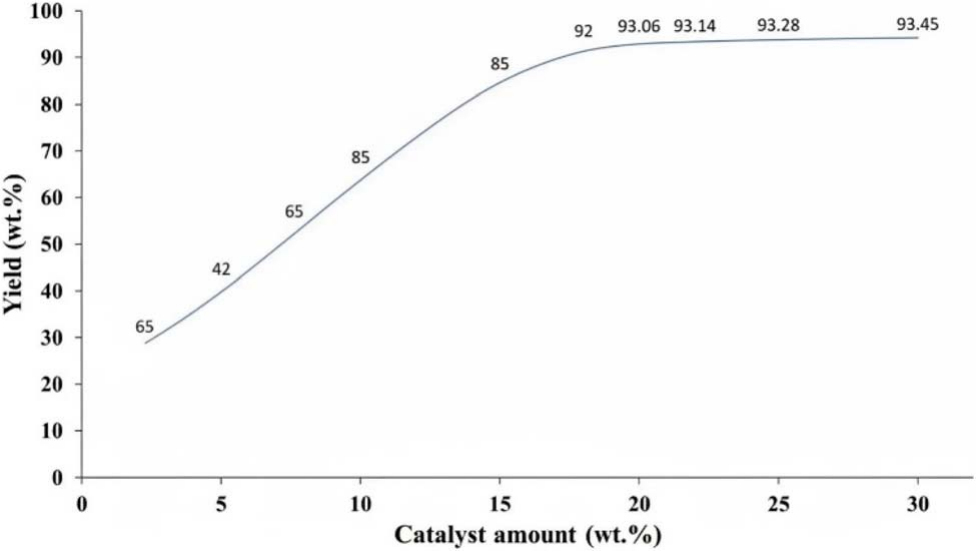
Effect of catalyst loading on the yield of 3-phenylisoquinoline (3a).

Fig. 8 demonstrates the influence of catalyst loading on product yield, revealing a marked increase in yield with increasing catalyst concentration up to 20 wt%. The highest yield of 93.45% is achieved at a catalyst loading of 28 wt%, suggesting an optimal loading range around 20–28 wt%. Beyond this range, the yield plateaus, indicating that further increases in catalyst concentration do not significantly enhance product formation and may introduce unnecessary costs and waste. This optimization of catalyst loading is a key principle in Green Chemistry, aiming to maximize efficiency and minimize environmental impact through responsible resource utilization. Further investigation into the catalyst's performance at various loadings would aid in refining the process for sustainable chemical synthesis.

**Fig. 9:**
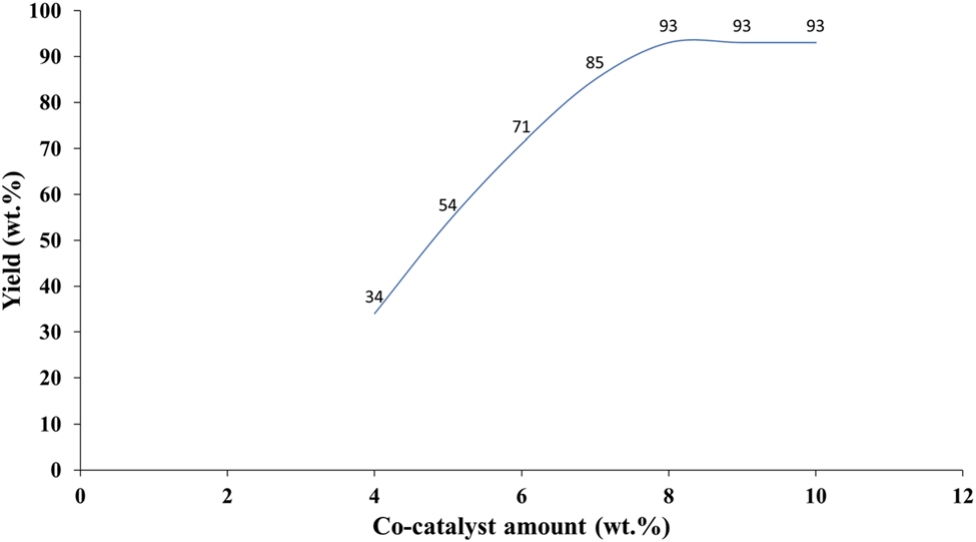
Effect of co-catalyst loading on the yield of 3-phenylisoquinoline (3a).

Fig. 9 illustrates the impact of co-catalyst loading on product yield, revealing a significant increase in efficiency as the co-catalyst concentration increases from 0 to 8 wt%.

The highest yield of 93 wt% is observed at co-catalyst loadings of 8 and 9 wt%, indicating an optimal range for enhanced catalytic activity. Beyond this point, the yield plateaus, suggesting that further increases in co-catalyst concentration do not lead to substantial improvements in product formation and may increase material costs. This optimization of co-catalyst loading is a crucial aspect of Green Chemistry, aiming to maximize process efficiency while minimizing the use of resources and potential waste generation. Further investigation into the synergistic effects of different co-catalysts at optimized loadings could lead to more sustainable and cost-effective chemical processes.

**Fig. 10:**
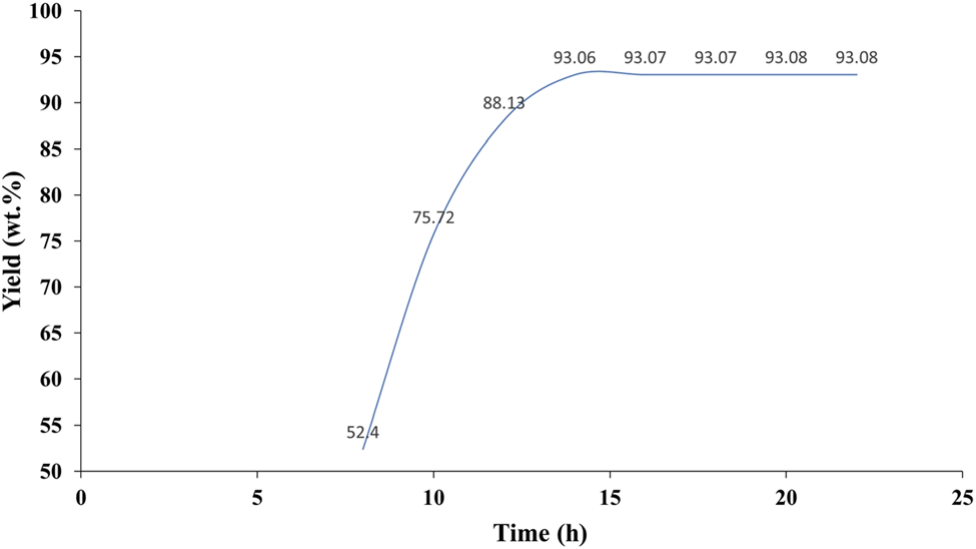
Effect of reaction time on the yield of 3-phenylisoquinoline (3a).

Fig. 10 illustrates the effect of reaction time on the yield of 3-phenylisoquinoline (3a). The yield exhibits a gradual increase initially, reaching 52.4% after 5 hours. A significant increase occurs between 5 and 10 hours, with the yield rising to 88.13%. The highest yield, approximately 93.08%, is achieved after 22 hours, suggesting a prolonged reaction period can further enhance product formation. However, the rate of yield increase appears to slow considerably after 14 hours, suggesting that 14 hours may represent a near-optimal reaction time, balancing product yield with reaction efficiency. This data indicates that optimizing reaction parameters to achieve efficient product synthesis is crucial. The observed plateau in yield after 14 hours indicates the approach of a kinetic limit or saturation of active sites, highlighting the importance of carefully balancing reaction time for maximized output and resource utilization.

**Fig. 11:**
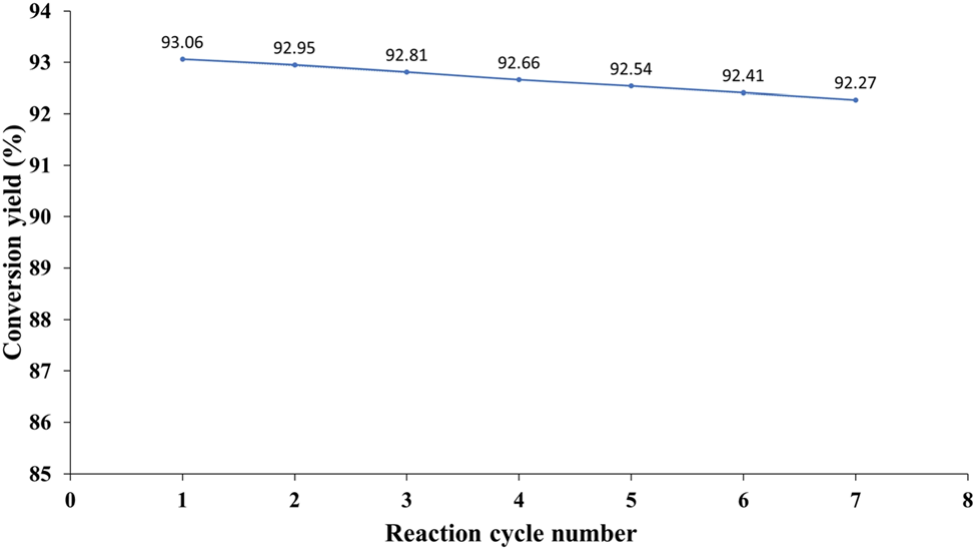
Catalyst stability and consistent conversion (92–93%) across seven reaction cycles for sustainable chemistry.

Fig. 11 demonstrates the stability of the catalyst through seven reaction cycles, showing a consistent conversion yield of approximately 92–93%. While a slight decrease in conversion is observed across cycles, the overall performance remains robust, indicating the catalyst's resilience to repeated use.

This stability is crucial for industrial applications, minimizing waste generation associated with catalyst replacement and contributing to sustainable chemical processes. The minor yield variation observed may be attributed to subtle changes in reaction conditions or catalyst surface properties over time, highlighting the importance of careful process control to maintain catalytic efficiency and minimize environmental impact. Further investigation into the catalyst's long-term stability under varying reaction conditions would be beneficial for optimizing its application in Green Chemistry protocols.

### Post-reaction morphological analysis

**Fig. 12:**
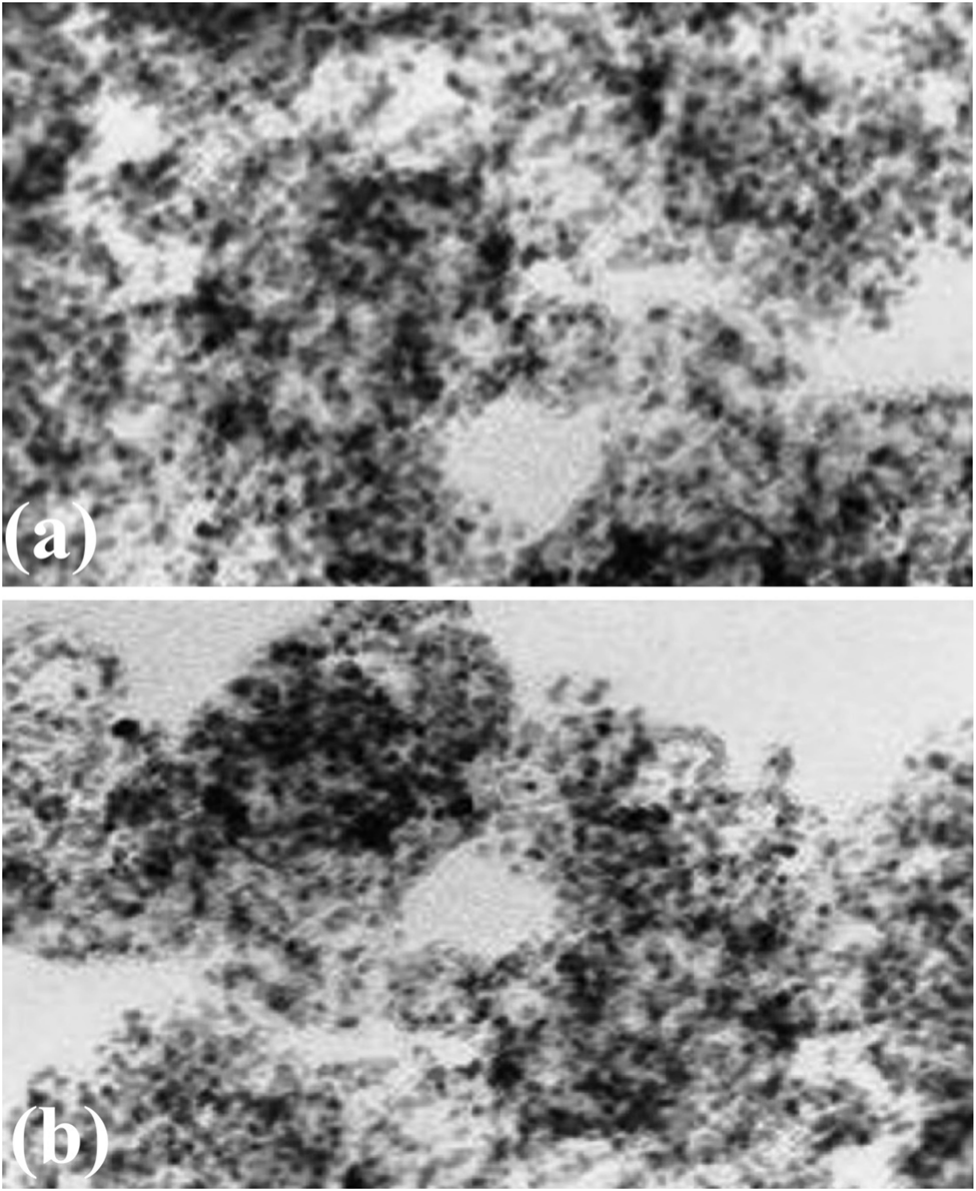
TEM images of the heterogeneous CuO/TiO_2_ catalyst (a) before use and (b) after seven catalytic cycles, showing preservation of nanoscale morphology and absence of significant particle aggregation.

To assess the robustness of the heterogeneous CuO/TiO_2_ catalyst under the optimized conditions, its morphology was examined by TEM before use and after seven consecutive catalytic cycles. As shown in Fig. 12, the fresh catalyst displays a distribution of finely dispersed CuO nanoparticles over the TiO_2_ support. After seven runs, the overall nanostructure remains essentially preserved: the CuO nanoparticles maintain their dispersed character without notable aggregation, particle coarsening, or collapse of the underlying TiO_2_ matrix. The absence of appreciable morphological deterioration indicates that the catalyst withstands the reaction conditions without significant structural degradation. Although XRD measurements could not be performed under the present constraints, the TEM analysis clearly demonstrates that the CuO/TiO_2_ catalyst retains its nanoscale morphology during repeated operation.

This morphological stability is in line with the experimental observation that the catalyst can be readily recovered and reused for at least seven consecutive cycles with minimal loss of activity.

### Leaching test

To examine the possible leaching of copper species from the heterogeneous CuO/TiO_2_ catalyst, a leaching experiment was performed under the optimized reaction conditions. The reaction was allowed to proceed for 5 h (approximately 40–50% conversion), after which the reaction mixture was rapidly centrifuged to separate the solid catalyst. The clear supernatant was then transferred to a new reaction vessel and allowed to react further under identical conditions in the absence of the solid catalyst. No significant increase in product formation was observed after additional stirring, as monitored by TLC analysis, indicating that the catalytic activity did not continue in the absence of the solid material. Furthermore, ICP analysis of the filtrate revealed only trace amounts of copper in the reaction solution. These results suggest that copper leaching from the CuO/TiO_2_ catalyst is minimal and that the catalytic transformation predominantly occurs on the surface of the heterogeneous catalyst.

### The optimization of reaction conditions

The optimization of reaction conditions for the synthesis of 3-phenylisoquinoline (3a) was systematically investigated, as detailed in Table 1. Crucially, the reaction's efficiency was found to be dependent on the simultaneous presence of the heterogeneous CuO/TiO_2_ catalyst, a homogeneous copper source (CuCl), a reducing agent (ascorbic acid), and visible light irradiation. Entries 1–7 clearly demonstrate that the absence of any one of these components either prevents the reaction entirely or results in negligible product formation, underscoring the photoredox and radical nature of the process. Subsequent studies focused on solvent selection, where greener solvents, particularly 95% ethanol, yielded the highest product yield (93%), significantly outperforming nonpolar or chlorinated solvents like toluene and dichloromethane (entries 11 and 12). The optimal loading for the CuO/TiO_2_ catalyst was determined to be 20 mg (entry 16). Variation in ascorbic acid concentration indicated that a range of 20–35 mol% provided consistently high yields, with lower amounts leading to a decrease in efficiency (entries 19 and 20). Further optimization of reaction time and temperature revealed that mild conditions, specifically room temperature to 35 °C and a reaction duration of 14–24 hours, were optimal, while elevated temperatures (50–70 °C) negatively impacted the yield (entries 21–24). Therefore, the established optimal conditions involve 20 mg of CuO/TiO_2_, 8 mol% CuCl, 20–35 mol% ascorbic acid in 95% ethanol under visible light irradiation at ambient temperature for 24 hours, aligning well with the principles of Green Chemistry through reduced energy consumption, use of environmentally benign solvents, and high process efficiency.

### Comparative assessment of light sources (LED *vs.* CFL)

To further evaluate the sustainability of the photochemical step, the reaction was examined under different light sources, including compact fluorescent lamp (CFL, 23 W, 6500 K) and a light-emitting diode (LED, 10 W, 450 nm). Both lamps provided visible-light irradiation sufficient for the activation of the CuO/TiO_2_ catalyst.

Under LED irradiation, the reaction proceeds with a slightly lower isolated yield (86%) compared to CFL (93%), but with significantly reduced energy consumption (10 W *vs.* 23 W), resulting in an approximately two-fold improvement in photonic efficiency (Table 2). Although the emission maximum of blue LEDs (450 nm) lies slightly above the intrinsic band-gap absorption of TiO_2_ (≈3.2 eV, *ca.* 390–400 nm), visible-light activation is enabled through CuO sensitization and defect states, which facilitate efficient charge separation while minimizing non-productive heating. Under these conditions, TiO_2_ may primarily function as a support that stabilizes and disperses the copper species, while the copper centers likely play a more dominant role in the catalytic process. In contrast, CFL sources emit a broader spectrum extending into the near-UV and infrared regions, leading to partial energy loss as heat. Consequently, the LED setup reduces the specific energy input from approximately 1.5 kWh mol^−1^ (CFL) to 0.7 kWh mol^−1^ (LED), indicating an overall improvement in the energy profile of the photochemical step. From a green chemistry perspective, the LED-based system aligns with several sustainability criteria—primarily reduced electricity usage, lower heat generation, and milder operating conditions—although it does not fully satisfy all green chemistry principles. Overall, replacing CFL lamps with narrow-band LEDs enhances the energy efficiency of the visible-light-induced isoquinoline synthesis, while further improvements may be achievable through continuous-flow photoreactors employing low-power LEDs to maximize photon utilization and further reduce environmental impact.

**Table 2:** Photophysical and catalytic performance comparison of different light sources

Light source	Power consumption (W)	Wavelength range (nm)	Emission maximum (nm)	Irradiance (mW cm^−2^)	Yield of 3a (%)	Photonic efficiency^a^	Sustainability remarks
CFL, household (23 W)	23	400–700	6500 K (white)	≈1.8	93	0.043	Readily available; moderate efficiency; minor UV component generates waste heat
LED panel (10 W, blue 450 nm)	10	430–470	450 nm	≈2.3	86	0.095	Higher photonic efficiency, lower power demand, negligible IR/UV; reduced energy consumption
Dark control	—	—	—	—	0	—	No reaction

a Photonic efficiency calculated as yield (%)/irradiance (mW cm^−2^ × power (W)) for comparative purposes, normalized to 1 h illumination.

With the optimized conditions established, the generality of the protocol was explored using a range of substituted phenylacetaldehydes and benzaldehydes (Table 3). Isoquinoline derivatives 3a–p were obtained in good to excellent yields, demonstrating the broad applicability of the method. The reaction outcome was found to be sensitive to electronic effects. Phenylacetaldehydes bearing electron-donating substituents (*e.g.*, methoxy) afforded higher yields than those containing electron-withdrawing groups such as fluoro substituents. Similarly, benzaldehydes with electron-donating substituents generally resulted in higher yields compared to their electron-deficient counterparts. These trends are consistent with the involvement of electron-rich intermediates in the oxidative coupling and cyclization steps.

**Table 3:** Reaction scope for the copper-catalyzed synthesis of isoquinoline derivatives (3a–p)^a^

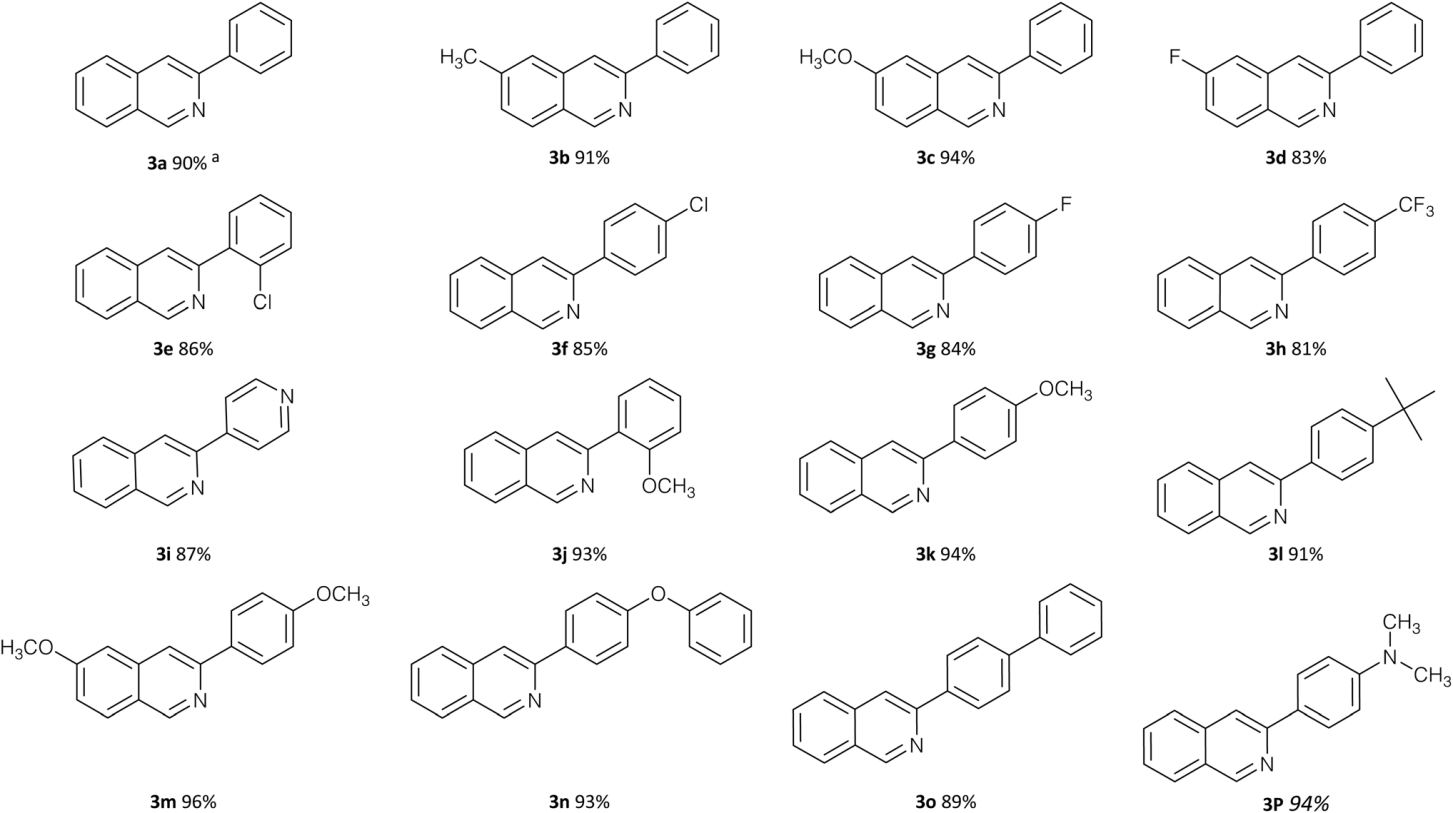

a All products (3a–p) were purified by column chromatography.

### Mechanistic investigations and proposed pathway

To gain insight into the reaction pathway, a series of mechanistic experiments were performed. The 1,4-diphenyl-2-azabutadiene intermediate was independently synthesized, isolated, and subjected to the optimized catalytic conditions, affording the corresponding isoquinoline in excellent yield. This result strongly supports the involvement of this species as a key intermediate in the reaction sequence.

The possible participation of radical intermediates was investigated through radical trapping experiments. When 2 equivalents of TEMPO were introduced under the standard reaction conditions, the formation of the isoquinoline product was almost completely suppressed, and only trace amounts of product were detected by TLC after 18 h. This pronounced inhibition suggests the involvement of radical species in the reaction pathway.

**Scheme 1:**
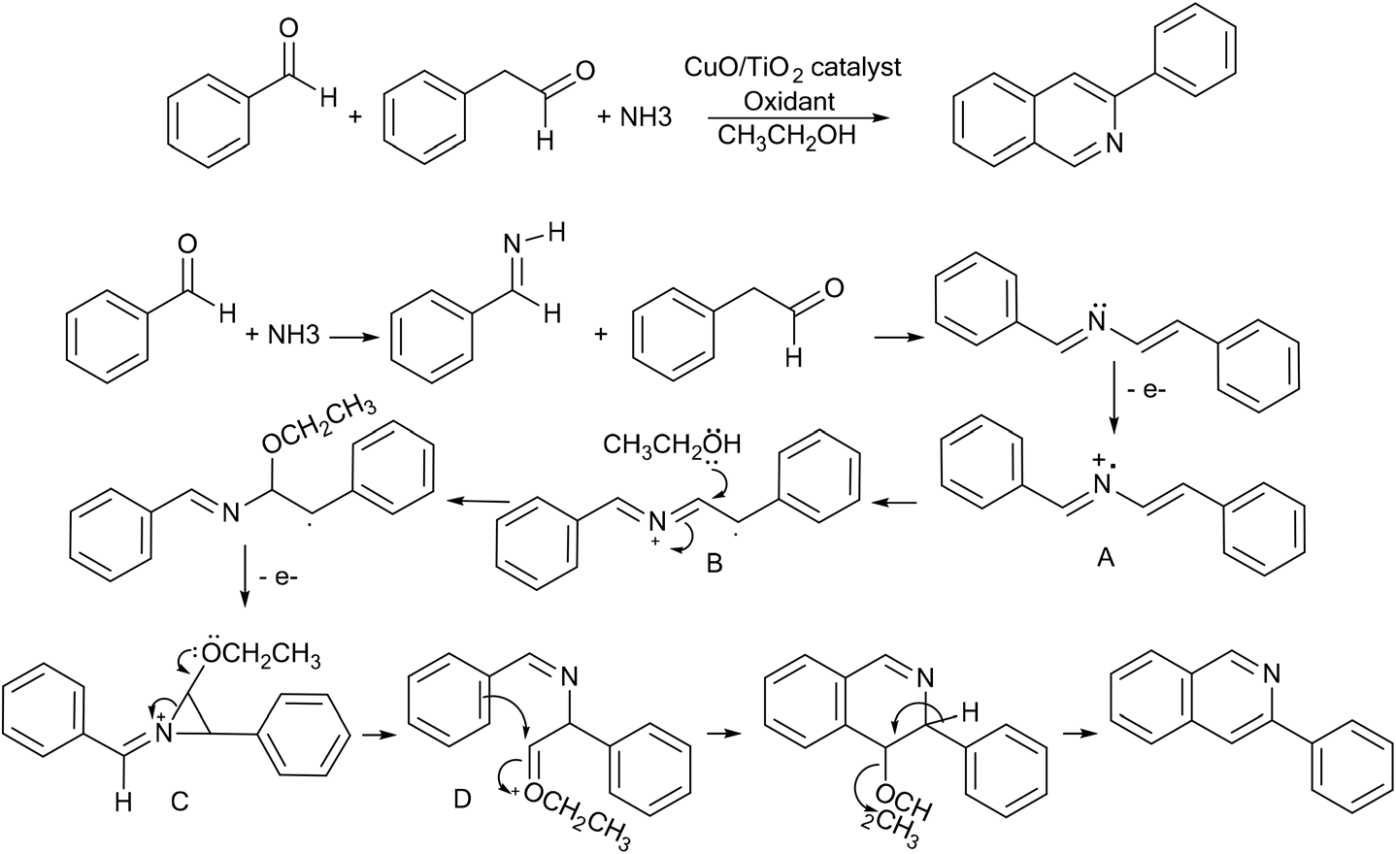
Proposed mechanistic pathway for the CuO/TiO_2_-photocatalyzed synthesis of isoquinolines. The mechanism involves the formation of an α-carbonyl radical, condensation to form the isolated 1,4-diphenyl-2-azabutadiene intermediate, followed by radical-mediated oxidative rearrangement and intramolecular cyclization.

Based on these observations, a plausible reaction pathway is proposed (Scheme 1). It should be noted that the TEMPO inhibition experiment alone does not provide conclusive mechanistic evidence; therefore, the proposed mechanism should be regarded as tentative and is suggested based on the experimental observations together with relevant precedents reported in the literature.^43^ Initially, Cu(i) species react with TBHP to generate *tert*-butoxy radicals along with higher-valent copper species. Subsequent hydrogen atom abstraction from phenylacetaldehyde affords an α-carbonyl radical intermediate. This intermediate undergoes condensation with ammonia and benzaldehyde to form the corresponding 1,4-diphenyl-2-azabutadiene intermediate.

Further single-electron transfer processes may generate cationic radical species, which subsequently undergo nucleophilic addition of methanol followed by oxidative rearrangement. The resulting intermediate then undergoes intramolecular cyclization and aromatization to furnish the isoquinoline framework.

In the catalytic system, CuO/TiO_2_ is assumed to function as a heterogeneous catalytic platform that provides active surface sites for the transformation. CuCl may participate in the Cu(i)/Cu(ii) redox cycle and facilitate the oxidative α-amination process through the formation of reactive copper species. Ascorbic acid likely acts as a reducing agent that promotes the *in situ* regeneration of catalytically active Cu(i) species, thereby sustaining the copper redox cycle under the reaction conditions. In addition, visible light may facilitate the formation of reactive radical intermediates and assist in the activation of the catalytic system. However, further mechanistic investigations would be required to fully elucidate the exact roles of these components.

Overall, the successful isolation of the azabutadiene intermediate together with the strong inhibitory effect observed in the presence of TEMPO collectively support a stepwise radical-mediated oxidative rearrangement and cyclization pathway leading to the formation of isoquinoline derivatives under the present catalytic conditions.

To quantitatively substantiate the sustainability claims of the present methodology, key green chemistry metrics were evaluated for the optimized copper-catalyzed one-pot synthesis of 3-phenylisoquinoline (3a). As summarized in Table S1 (SI), the multicomponent, single-step protocol exhibits a high theoretical atom economy (≈78%) and a favorable reaction mass efficiency (≈72%), reflecting efficient incorporation of starting materials into the target heterocycle. The avoidance of stoichiometric activating reagents, protecting groups, and strongly acidic media, together with the use of ethanol as a recommended green solvent, results in a qualitatively low *E*-factor. Energy consumption analysis further reveals a specific energy input of approximately 1.5 kWh mol^−1^ under CFL irradiation, which can be reduced to *ca.* 0.7 kWh mol^−1^ by employing low-power visible-light LEDs, corresponding to an energy reduction of about 50%. In addition, the heterogeneous CuO/TiO_2_ catalyst can be readily recovered and reused for at least seven consecutive cycles with minimal loss of activity, further enhancing the overall sustainability profile of the protocol.

Compared with classical isoquinoline syntheses such as the Pomeranz–Fritsch, Bischler–Napieralski, and Pictet–Spengler reactions, the present methodology operates under markedly milder and more sustainable conditions (Table 4). The avoidance of strong mineral acids, stoichiometric dehydrating agents, and elevated temperatures, combined with a one pot multicomponent design and visible light activation, results in significantly reduced waste generation and improved atom efficiency.

**Table 4:** Comparison of representative classical isoquinoline syntheses with the present copper-catalyzed multicomponent strategy from a green chemistry perspective

Method	Key starting materials	Reaction conditions	Hazardous reagents/media	Steps	Energy input	Waste generation	Green chemistry assessment
Pomeranz–Fritsch	Benzaldehyde + aminoacetal	Strong aqueous acid (≈6 M), >100 °C	Concentrated mineral acids	≥2	High (heating)	High (acid neutralization, aqueous waste)	Poor: harsh conditions, high *E*-factor
Bischler–Napieralski	β-Arylethylamides	POCl_3_/P_2_O_5_, reflux	Stoichiometric toxic dehydrating agents	≥2	High	High (stoichiometric waste)	Poor: hazardous reagents, low atom economy
Pictet–Spengler	β-Arylethylamines + aldehydes	Strong Brønsted or Lewis acids	TFA, AlCl_3_, HCl	≥2	Moderate–high	Moderate–high	Moderate: acidic media, limited sustainability
This work	Phenylacetaldehydes + benzaldehydes + NH_3_	r.t., visible light, EtOH (95%)	None (earth-abundant Cu catalyst)	1 (one-pot)	Low	Low	Excellent: mild, catalytic, high efficiency

## Experimental

All starting materials, solvents, and reagents were purchased from Sigma–Aldrich and used as received unless otherwise stated. ^1^H and ^13^C NMR spectra were recorded on a Bruker AVANCE spectrometer operating at 500 MHz and 125 MHz, respectively. Chemical shifts (*δ*) are reported in parts per million (ppm) relative to tetramethylsilane (TMS) as the internal standard.

### General procedure for the visible-light-induced synthesis of 3-phenylisoquinoline

In a 25 mL two-necked round-bottom flask equipped with a magnetic stirring bar, the corresponding phenylacetaldehyde (1.20 g, 10 mmol) was dissolved in ethanol 95% (10 mL). Subsequently, ammonia solution in methanol (4 M, 0.25 mL, 1.0 mmol), CuCl (8 mol%), ascorbic acid (25 mol%), and CuO/TiO_2_ photocatalyst (20 mg) were added sequentially. The reaction mixture was degassed by bubbling nitrogen gas for 30 min to establish an inert atmosphere. The flask was then sealed with a rubber septum and irradiated using a 23 W household fluorescent lamp (6500 K) positioned at a fixed distance of approximately 2 cm from the reaction vessel; the same distance was maintained for all light sources. The reaction was carried out at room temperature under continuous magnetic stirring for 18 h, and the progress was monitored by thin-layer chromatography (TLC). Upon completion of the reaction, the heterogeneous photocatalyst was separated by centrifugation at 7500 rpm for 10 min, washed thoroughly with methanol, and dried for reuse. The combined organic layers were dried over anhydrous sodium sulfate, filtered, and concentrated under reduced pressure using a rotary evaporator. The resulting residue was purified by column chromatography on silica gel eluting with isooctane/ethyl acetate (4 : 1, v/v) to afford product (3a) as a solid (1.90 g, 93% yield).

#### 3-Phenylisoquinoline (3a)

White solid, mp: 101–102 °C.

^1^H NMR (500 MHz, CDCl_3_): *δ* 9.35 (s, 1H), 8.18–8.12 (m, 2H), 8.06 (s, 1H), 7.97 (d, *J* = 8.5 Hz, 1H), 7.85 (d, *J* = 8.0 Hz, 1H), 7.70–7.65 (m, 1H), 7.60–7.55 (m, 1H), 7.55–7.50 (m, 2H) 7.45–7.40 (m, 1H) ppm.

^13^C NMR (125 MHz, CDCl_3_): *δ* 152.5, 151.3, 139.7, 136.7, 130.6, 128.9, 128.6, 127.8, 127.7, 127.2, 127.1, 127.0, 116.6 ppm.

HRMS (ESI) *m*/*z*: calcd for C_15_H_12_N [M + H]^+^: 206.0964. Found: 206.0966.

#### 6-Methyl-3-phenylisoquinoline (3b)

Yield: 2.00 g (91%), yellow solid, mp: 115–116 °C.

^1^H NMR (500 MHz, CDCl_3_): *δ* 9.26 (s, 1H), 8.12 (d, *J* = 7.5 Hz, 2H), 8.02 (s, 1H), 7.80–7.70 (m, 2H), 7.55–7.45 (m, 3H), 7.41 (t, *J* = 7.5 Hz, 1H), 2.55 (s, 3H) ppm.

^13^C NMR (125 MHz, CDCl_3_); *δ* 151.8, 150.5, 139.7, 137.3, 135.1, 133.1, 128.9, 128.5, 128.1, 127.0, 126.9, 126.5, 116.6, 22.0 ppm.

HRMS (ESI) *m*/*z*: calcd for C_16_H_14_N [M + H]^+^: 220.1121. Found: 220.1127.

#### 6-Methoxy-3-phenylisoquinoline (3c)

Yield: 2.21 g (94%), light yellow solid, mp: 101–102 °C.

^1^H NMR (500 MHz, CDCl_3_): *δ* 9.19 (s, 1H), 8.11 (dd, *J* = 8.0,1.5 Hz, 2H), 7.97 (s, 1H), 7.87 (d, *J* = 9.0 Hz, 1H), 7.50 (tt, *J* = 7.5,1.5 Hz 2H), 7.42 (tt, *J* = 7.0,1.5 Hz 1H), 7.20 (dd, *J* = 8.5,2.0 Hz 1H), 7.11 (sd, *J* = 2.0 Hz, 1H), 3.96 (s, 3H) ppm.

^13^C NMR (125 MHz, CDCl_3_): *δ* 161.3, 151.8, 151.6, 139.8, 138.8, 129.4, 128.9, 128.6, 127.1, 123.7, 120.4, 116.1, 104.5, 55.6 ppm.

HRMS (ESI) *m*/*z*: calcd for C_16_H_14_NO [M + H]^+^: 236.1070. Found: 236.1065.

#### 6-Fluoro-3-phenylisoquinoline (3d)

Yield: 1.85 g (83%), light yellow solid, mp: 123–124 °C.

^1^H NMR (500 MHz, CDCl_3_): *δ* 9.29 (s, 1H), 8.11 (d, *J* = 8.5 Hz, 2H), 8.50–7.93 (m, 2H), 7.52 (t, *J* = 8.5 Hz 2H), 7.48–7.40 (m, 2H), 7.33 (td, *J* = 8.5,2.0 Hz, 1H) ppm.

^13^C NMR (125 MHz, CDCl_3_): *δ* 163.5 (d, *J* = 252.5 Hz), 152.10 152.06, 139.2, 138.2 (d, *J* = 10.6 Hz), 130.6 (d, *J* = 9.9 Hz), 128.9, 127.1, 125.0, 117.7 (d, *J* = 25.9 Hz), 116.1 (d, *J* = 5.5 Hz), 110.30 (d, *J* = 21.1 Hz) ppm.

HRMS (ESI) *m*/*z*: Calcd for C_15_H_11_FN [M + H]^+^: 224.0870. Found: 224.0875.

#### 3-(2-Chlorophenyl)isoquinoline (3e)

Yield: 2.13 g (86%), white solid, mp: 45–46 °C.

^1^H NMR (500 MHz, CDCl_3_): *δ* 9.36 (s, 1H), 8.05–8.00 (m, 2H), 8.87 (d, *J* = 8.0 Hz, 1H), 7.76–7.68 (m, 2H), 7.66 (m, 1H), 7.52 (dd, *J* = 8.0,1.5 Hz, 1H), 7.39 (td, *J* = 7.5,1.5 Hz 1H), 7.34 (td, *J* = 7.5,2.0 Hz 1H) ppm.

^13^C NMR (125 MHz, CDCl_3_): *δ* 152.2, 150.3, 139.3, 136.0, 132.6, 132.0, 130.8, 130.3, 129.4, 127.71, 127.67, 127.66, 127.11, 127.07, 121.4 ppm.

HRMS (ESI) *m*/*z*: Calcd for C_15_H_11_ClN [M + H]^+^: 240.0574. Found: 240.0575.

#### 3-(4-Chlorophenyl)isoquinoline (3f)

Yield: 2.04 g (85%), light yellow solid, mp: 141–142 °C.

^1^H NMR (500 MHz, CDCl_3_): *δ* 9.32 (s, 1H), 8.07 (d, *J* = 8.5 Hz 2H), 8.03 (s, 1H), 7.98 (d, *J* = 8.0 Hz 1H), 7.86 (d, *J* = 8.0 Hz 1H), 7.67–7.73 (m, 1H), 7.62–7.56 (m, 1H), 7.47 (d, *J* = 8.5 Hz 2H)ppm.

^13^C NMR (125 MHz, CDCl_3_): *δ* 152.5, 150.0, 137.9, 136.7, 134.8, 131.0, 129.1, 128.4, 127.9, 127.8, 127.5, 127.1, 116.7 ppm.

HRMS (ESI) *m*/*z*: calcd for C_15_H_11_ClN [M + H]^+^: 240.0574. Found: 240.0573.

#### 3-(4-Fluorophenyl)isoquinoline (3g)

Yield: 1.88 g (84%), yellow solid, mp: 107–108 °C. ^1^H NMR (500 MHz, CDCl_3_): *δ* 9.31 (s, 1H), 8.13–8.07 (m, 2H), 8.02–7.95 (m, 2H), 7.85(d, *J* = 8.0 Hz 1H), 7.72–7.66 (m, 1H), 7.62–7.56 (m, 1H), 7.19 (t, *J* = 9.0 Hz 2H) ppm.

^13^C NMR (101 MHz, CDCl_3_): *δ* 163.4 (d, *J* = 222.3 Hz), 152.5, 150.3, 136.8, 135.7 (d, *J* = 3.0 Hz), 130.9, 128.9 (d, *J* = 8.2 Hz), 127.7, 127.3, 127.0, 116.4, 115.8 (d, *J* = 21.8 Hz) ppm.

HRMS (ESI) *m*/*z*: calcd for C_15_H_11_FN [M + H]^+^: 224.0870. Found: 224.0874.

#### 3-(4-(Trifluoromethyl)phenyl)isoquinoline (3h)

Yield: 2.21 g (81%), yellow solid, mp: 158–159 °C.

^1^H NMR (500 MHz, CDCl_3_): *δ* 9.34 (s, 1H), 8.23 (d, *J* = 8.0 Hz, 2H), 8.08 (s, 1H), 7.99 (d, *J* = 8.0 Hz, 1H), 7.87 (d, *J* = 8.5 Hz, 1H), 7.76–7.70 (m, 3H), 7.64–7.58 (m, 1H) ppm.

^13^C NMR (125 MHz, CDCl_3_): *δ* 152.8, 149.6, 143.0, 136.6, 131.0, 130.4 (d, *J* = 45.4 Hz), 129.1(d, *J* = 146.8 Hz), 128.2, 127.8, 127.7, 127.3, 127.2, 125.8 (q, *J* = 3.9 Hz), 117.4 ppm.

HRMS (ESI) *m*/*z*: calcd for C_16_H_11_F_3_N [M + H]^+^: 274.0838. Found: 274.0831.

#### 3-(Pyridin-4-yl)isoquinoline (3i)

Yield: 1.79 g (87%), yellow solid, mp: 127–128 °C.

^1^H NMR (500 MHz, CDCl_3_): *δ* 9.32 (s, 1H), 8.74–8.69 (m, 2H), 8.12 (s, 1H), 8.02–7.95 (m, 3H), 7.87 (d, *J* = 8.0 Hz 1H), 7.74–7.68 (m, 1H), 7.65–7.60 (m, 1H) ppm.

^13^C NMR (125 MHz, CDCl_3_): *δ* 152.9, 150.4, 148.3, 146.8, 136.3, 131.0, 128.6, 128.2, 127.7, 127.3, 121.2, 117.8 ppm.

HRMS (ESI) *m*/*z*: Calcd for C_14_H_11_N_2_ [M + H]^+^: 207.0917. Found: 207.0915.

#### 3-(2-Methoxyphenyl)isoquinoline (3j)

Yield: 2.19 g (93%), yellow solid, mp: 129–130 °C.

^1^H NMR (500 MHz, CDCl_3_): *δ* 9.36 (s, 1H), 8.22 (s, 1H), 8.00–7.92 (m, 2H), 7.84 (d, *J* = 8.5 Hz, 1H), 7.70–7.63 (m, 1H), 7.60–7.52 (m, 1H), 7.43–7.36 (m, 1H), 7.18–7.12 (m, 1H), 7.04 (d, *J* = 8.5 Hz 1H), 3.89 (s, 3H) ppm.

^13^C NMR (125 MHz, CDCl_3_): *δ* 157.1, 151.9, 149.2, 136.2, 131.5, 130.3, 129.6, 129.1, 127.43, 127.36, 127.03, 127.01, 121.2, 121.1, 111.4, 55.7 ppm.

HRMS (ESI) *m*/*z*: calcd for C_16_H_14_NO [M + H]^+^: 236.1070. Found: 236.1061.

#### 3-(4-Methoxyphenyl)isoquinoline (3k)

Yield: 2.21 g (94%), yellow solid, mp: 100–101 °C.

^1^H NMR (500 MHz, CDCl_3_): *δ* 9.31 (s, 1H), 8.08 (d, *J* = 9.0 Hz 2H), 7.98 (s, 1H), 7.96 (d, *J* = 8.5, 1H), 7.83 (d, *J* = 8.5, 1H), 7.69–7.64 (m, 1H), 7.57–7.52 (m, 1H), 7.04 (d, *J* = 8.5 2H), 3.88 (s, 3H) ppm.

^13^C NMR (125 MHz, CDCl_3_): *δ* 160.3, 152.3, 151.0, 137.0, 132.1, 130.7, 128.4, 127.8, 127.5, 126.9, 115.6, 114.3, 55.5 ppm.

HRMS (ESI) *m*/*z*: Calcd for C_16_H_14_NO [M + H]^+^: 236.1070. Found: 236.1066.

#### 3-(4-(*tert*-Butyl)phenyl)isoquinoline (3l)

Yield: 2.38 g (91%), yellow solid, mp: 74–75 °C.

^1^H NMR (500 MHz, CDCl_3_): *δ* 9.35 (s, 1H), 8.10 (d, *J* = 8.5, 2H), 8.05 (s, 1H), 7.97 (d, *J* = 8.0 Hz, 1H), 7.85 (d, *J* = 8.0 Hz, 1H), 7.69–7.64 (m, 1H), 7.59–7.52 (m, 3H), 1.42 (s, 9H) ppm.

^13^CNMR (125 MHz, CDCl_3_): *δ* 152.4, 151.7, 151.3, 136.82, 136.77, 130.5, 127.72, 127.66, 127.0, 126.8, 125.9, 116.2, 34.8, 31.4 ppm.

HRMS (ESI) *m*/*z*: calcd for C_19_H_20_N [M + H]^+^: 262.1590. Found: 262.1596.

#### 6-Methoxy-3-(4-methoxyphenyl)isoquinoline (3m)

Yield: 2.55 g (96%), yellow solid, mp: 113–114 °C.

^1^H NMR (500 MHz, CDCl_3_): *δ* 9.16 (s, 1H), 8.08 (d, *J* = 9.0 Hz, 2H), 7.89 (s, 1H), 7.83 (d, *J* = 8.5 Hz, 1H), 7.21 (d, *J* = 8.0 Hz, 1H), 7.08 (d, *J* = 8.5 Hz, 1H), 7.04 (d, *J* = 8.5 Hz, 2H), 3.95 (s, 3H), 3.88 (s, 3H) ppm.

^13^C NMR (125 MHz, CDCl_3_): *δ* 161.3, 160.3, 151.0, 139.1, 132.1, 130.7, 128.5, 128.3, 123.3, 120.4, 115.6, 114.3, 104.3, 55.5, 55.0 ppm.

HRMS (ESI) *m*/*z*: calcd for C_17_H_16_NO_2_ [M + H]^+^: 266.1176. Found, 266.1168.

#### 3-(4-Phenoxyphenyl)isoquinoline (3p)

Yield: 2.76 g (93%), brown solid, mp: 106–107 °C.

^1^H NMR (500 MHz, CDCl_3_): *δ* 9.33 (s, 1H), 8.12 (d, *J* = 9.0 Hz 2H), 8.02 (s, 1H), 7.97 (dd, *J* = 8.0,1.0 Hz, 1H), 7.85 (dd, *J* = 8.0,1.0 Hz, 1H), 7.70–7.66 (m, 1H), 7.60–7.55 (m, 1H), 7.41–7.35 (m, 2H), 7.18–7.12 (m, 3H), 7.12–7.08 (m, 2H) ppm.

^13^C NMR (125 MHz, CDCl_3_): *δ* 157.9, 157.1, 152.5, 150.8, 136.8, 134.8, 130.7, 129.9, 128.6, 127.69, 127.66, 127.1, 126.9, 123.6, 119.2, 119.1, 116.0 ppm.

HRMS (ESI) *m*/*z*: calcd for C_21_H_16_NO [M + H]^+^: 298.1226. Found: 298.1221.

#### 3-([1,1′-Biphenyl]-4 yl)isoquinoline (3o)

Yield: 2.50 g (89%), yellow solid, mp: 97 – 99 °C.

^1^H NMR (500 MHz, CDCl_3_): *δ* 9.37 (s, 1H), 8.24 (d, *J* = 8.0,1.0 Hz, 2H), 8.11 (s, 1H), 7.99 (d, *J* = 8.5 Hz, 1H), 7.86 (d, *J* = 8.5 Hz, 1H), 7.77 (d, *J* = 8.0, 2H), 7.73–7.65 (m, 3H), 7.58 (t, *J* = 8.0 1H), 7.49 (t, *J* = 7.5 Hz, 2H), 7.39 (t, *J* = 7.5 Hz, 1H) ppm.

^13^C NMR (125 MHz, CDCl_3_): *δ* 152.5, 150.8, 141.4, 140.8, 138.5, 136.8, 130.7, 128.9, 127.9, 127.7, 127.62, 127.58, 127.5, 127.24, 127.20, 127.0, 116.6 ppm.

HRMS (ESI) *m*/*z*: calcd for C_21_H_16_N [M + H]^+^: 282.1277. Found: 282.1272.

#### 4-(Isoquinolin-3-yl)-*N*,*N*-dimethylaniline (3af)

Yield: 2.33 g (94%), light yellow solid, mp: 138–139 °C.

^1^H NMR (500 MHz, CDCl_3_): *δ* 9.29 (s, 1H), 8.06 (d, *J* = 9.0 Hz, 2H), 7.97–7.92 (m, 2H), 7.81 (dd, *J* = 8.5, 1.0 Hz, 1H), 7.66–7.62 (m, 1H), 7.52–7.48 (m, 1H), 6.85 (d, *J* = 9.0 Hz 2H), 3.04 (s, 6H) ppm.

^13^C NMR (125 MHz, CDCl_3_); *δ* 152.1, 151.5, 150.9, 137.1, 130.6, 127.9, 127.7, 127.3, 127.2, 126.8, 126.4, 114.5, 112.6, 40.6 ppm.

HRMS (ESI) *m*/*z*: calcd for C_17_H_17_N_2_ [M + H]^+^: 249.1386. Found: 249.1382.

## Conclusions

In summary, an efficient, sustainable, and operationally simple multicomponent strategy has been developed for the synthesis of isoquinoline derivatives from inexpensive and readily available starting materials. This methodology is based on a copper-catalyzed oxidative α-amination of phenylacetaldehyde derivatives, employing a commercially available ammonia solution in methanol as a practical and comparatively benign nitrogen source. The transformation proceeds under remarkably mild conditions at room temperature and under visible-light irradiation, avoiding the use of strong acids, stoichiometric activating reagents, and elevated temperatures. The one-pot multicomponent design, combined with the use of an earth-abundant and recyclable heterogeneous CuO/TiO_2_ catalyst, affords a broad range of isoquinolines in good to excellent yields with improved atom economy, reduced waste generation, and enhanced energy efficiency. Mechanistic studies, including the isolation of a key 1,4-diphenyl-2-azabutadiene intermediate and radical trapping experiments, support a stepwise radical-mediated pathway. Overall, the integration of visible-light activation, mild reaction conditions, heterogeneous copper catalysis, and multicomponent efficiency renders this protocol a valuable and environmentally considerate alternative to classical isoquinoline syntheses, with promising potential for applications in heterocycle synthesis and medicinal chemistry.

## Author contributions

A. A.: conceptualization, supervision, formal analysis, manuscript revision. D. Z. H. S.: experimental investigation, data curation, and original draft preparation.

## Conflicts of interest

There are no conflicts to declare.

## Supplementary Material

RA-016-D6RA01207H-s001

## Data Availability

All data supporting the findings of this study are available within the article and its supplementary information (SI). Supplementary information: the calculation and assessment of green chemistry metrics for the copper-catalyzed one-pot synthesis of isoquinolines, including atom economy, reaction mass efficiency, energy input analysis, catalyst recyclability, and overall sustainability indicators (Table S1). It also provides full characterization data for all synthesized isoquinoline derivatives, including ^1^H and ^13^C NMR spectra and high-resolution mass spectrometry (HRMS) data. See DOI: https://doi.org/10.1039/d6ra01207h.
