# Supplementary material for: Copper-catalyzed one-pot synthesis of isoquinolines via oxidative α-amination under mild and sustainable conditions
Source: RSC Adv. 2026 Apr 24;16(23):21188–200. doi: 10.1039/d6ra01207h (PMC13108578; doi:10.1039/d6ra01207h)
Supplement: RA-016-D6RA01207H-s001 [file RA-016-D6RA01207H-s001.pdf]

Supplementary Information (SI) for RSC Advances.
This journal is © The Royal Society of Chemistry 2026

Copper-Catalyzed One-Pot Synthesis of Isoquinolines *via* Oxidative α-Amination Under Mild and Sustainable Conditions

Ali Akbari [a, ∗], Danial Zand Hoseinshahi[a]

Department of Chemistry, Faculty of Science, University of Jiroft, Jiroft, P. O. Box 8767161167, Iran. Tel.: +98-344-334-7061; fax: +98-344-334-7065; e-mail: a.akbari@ujiroft.ac.ir

ABSTRACT

A highly sustainable and operationally simple, copper-catalyzed multicomponent strategy is reported for the synthesis of structurally diverse isoquinolines from inexpensive and readily available phenylacetaldehyde derivatives, benzaldehydes, and ammonia (using 4 M NH_3 in methanol as a convenient nitrogen source). The transformation proceeds under remarkably mild conditions (room temperature), constituting a modified Pomeranz–Fritsch type cyclization that enables direct one-pot access to the target scaffolds in good to excellent yields. The protocol notably avoids harsh acidic conditions and prefunctionalized amine substrates. Mechanistic studies confirm a stepwise pathway involving the isolation of a key 1,4-diphenyl-2-azabutadiene intermediate and provide compelling evidence for radical involvement via TEMPO trapping. The combination of high atom efficiency, use of an earth-abundant copper catalyst, mild operational parameters, and mechanistic understanding demonstrates a practical and environmentally appealing approach to constructing important isoquinoline frameworks.

Calculation and assessment of green-chemistry metrics

Atom economy (AE) was calculated based on the stoichiometric incorporation of phenylacetaldehyde, benzaldehyde, and ammonia into the isoquinoline framework, while excluding catalytic species and solvent from the calculation. Reaction mass efficiency (RME) was estimated from the isolated yield and the theoretical atom economy of the reaction.

Energy input values were calculated by multiplying the electrical power of the irradiation source by the total irradiation time and normalizing the value per mole of product formed. For comparison, energy consumption was evaluated for both CFL (23 W) and LED (10 W) light sources under the optimized reaction conditions.

The E factor was assessed qualitatively due to the complexity of accurately quantifying all waste streams. The evaluation considered the absence of stoichiometric activating reagents, the use of ethanol as the reaction solvent, the one-pot nature of the transformation, and the recyclability of the CuO/TiO_2 catalyst.

∗ Corresponding author.
E-mail address: author@university.edu

Table S1. Quantitative green chemistry metrics (AE, RME, energy input, catalyst recyclability, and sustainability indicators) for the optimized copper-catalyzed one-pot synthesis of 3-phenylisoquinoline (3a).

**Metric**	**Value**	**Basis / Notes**
Reaction type	Multicomponent, one pot	Avoids isolation of intermediates; reduces operational steps
Number of steps	1	Direct conversion to isoquinoline
Catalyst	CuO/TiO_2 (heterogeneous)	Earth-abundant support; recyclable under the tested conditions
Catalyst loading	20 mg (≈5 wt% Cu)	Reused for ≥7 cycles with minor performance decline
Solvent	EtOH (95%)	Relatively low-toxicity solvent; generally considered a preferable option
Temperature	Room temperature	No external heating required under optimized conditions
Light source	CFL (23 W) / LED (10 W)	Visible-light irradiation; LED reduces energy input
Reaction time	14–24 h	Optimized at 14 h
Isolated yield (3a)	93% (CFL), 86% (LED)	Corresponds to Table 1, Entry 25
Atom Economy (AE)	≈78%	Calculated from overall stoichiometry
Reaction Mass Efficiency (RME)	≈72%	AE × isolated yield
Stoichiometric reagents	None	Only catalytic quantities of additives used
Protecting groups	None	Direct transformation
E factor (qualitative)	Moderate	Simple workup; solvent and oxidant still contribute to waste
Energy input (CFL)	≈1.5 kWh mol^{-1}	23 W × 24 h
Energy input (LED)	≈0.7 kWh mol^{-1}	10 W × 14 h
Energy reduction (LED vs CFL)	~53%	Normalized energy comparison
Catalyst recyclability	≥7 cycles	Yield maintained at ≈92–93% across cycles
Overall sustainability assessment	Moderately good	Meets several sustainability criteria (reduced energy, mild conditions, one-pot design) but **does not fully satisfy all green chemistry principles**

Characterization data for isoquinoline derivative

3-Phenylisoquinoline (3a). Yield: 1.84 g (90%), white solid, mp: 101 – 102 °C.
^{1}H NMR (500 MHz, $CDCl_3$): δ 9.35 (s, 1H), 8.18 – 8.12 (m, 2H), 8.06 (s, 1H), 7.97 (d, *J* = 8.5 Hz, 1H), 7.85 (d, *J* = 8.0 Hz, 1H), 7.70 – 7.65 (m, 1H), 7.60 – 7.55 (m, 1H), 7.55 – 7.50 (m, 2H) 7.45 – 7.40 (m, 1H) ppm.
^{13}C NMR (125 MHz, $CDCl_3$): δ 152.5, 151.3, 139.7, 136.7, 130.6, 128.9, 128.6, 127.8, 127.7, 127.2, 127.1, 127.0, 116.6 ppm.
HRMS (ESI) m/z: Calcd for $C_{15}H_{12}N$ $[M+H]^+$: 206.0964. Found: 206.0966.
6-Methyl-3-phenylisoquinoline (3b). Yield: 2.00 g (91%), yellow solid, mp: 115 –116 °C.
^{1}H NMR (500 MHz, $CDCl_3$): *δ* 9.26 (s, 1H), 8.12 (d, *J* = 7.5 Hz, 2H), 8.02 (s, 1H), 7.80– 7.70 (m, 2H), 7.55 – 7.45 (m, 3H), 7.41 (t, *J* = 7.5 Hz, 1H), 2.55 (s, 3H) ppm.
^{13}C NMR (125 MHz, $CDCl_3$); *δ* 151.8, 150.5, 139.7, 137.3, 135.1, 133.1, 128.9, 128.5, 128.1, 127.0, 126.9, 126.5, 116.6, 22.0 ppm.
HRMS (ESI) m/z: Calcd for $C_{16}H_{14}N$ $[M+H]^+$: 220.1121. Found: 220.1127.
6-Methoxy-3-phenylisoquinoline (3c). Yield: 2.21 g (94%), Light yellow solid, mp: 101 –102 °C.
^{1}H NMR (500 MHz, $CDCl_3$): *δ* 9.19 (s, 1H), 8.11 (dd, *J* = 8.0,1.5 Hz, 2H), 7.97 (s, 1H), 7.87 (d, *J* = 9.0 Hz, 1H), 7.50 (tt, *J* = 7.5,1.5 Hz 2H), 7.42 (tt, *J* = 7.0,1.5 Hz 1H), 7.20 (dd, *J* = 8.5,2.0 Hz 1H), 7.11 (sd, *J* = 2.0 Hz, 1H), 3.96 (s, 3H) ppm.
^{13}C NMR (125 MHz, $CDCl_3$): *δ* 161.3, 151.8, 151.6, 139.8, 138.8, 129.4, 128.9, 128.6, 127.1, 123.7, 120.4, 116.1, 104.5, 55.6 ppm.
HRMS (ESI) m/z: Calcd for $C_{16}H_{14}NO$ $[M+H]^+$: 236.1070. Found: 236.1065.
6-Fluoro-3-phenylisoquinoline (3d). Yield: 1.85 g (83%), Light yellow solid, mp: 123 –124 °C.
^{1}H NMR (500 MHz, $CDCl_3$): *δ* 9.29 (s, 1H), 8.11 (d, *J* = 8.5 Hz, 2H), 8.50 – 7.93 (m, 2H), 7.52 (t, *J* = 8.5 Hz 2H), 7.48 – 7.40 (m, 2H), 7.33 (td, *J* = 8.5,2.0 Hz, 1H) ppm.
^{13}C NMR (125 MHz, $CDCl_3$): *δ* 163.5 (d, *J*= 252.5 Hz), 152.10 152.06, 139.2, 138.2 (d, *J* = 10.6 Hz), 130.6 (d, *J* = 9.9 Hz), 128.9, 127.1, 125.0, 117.7 (d, *J* = 25.9 Hz), 116.1 (d, *J* = 5.5 Hz), 110.30 (d, *J*= 21.1 Hz) ppm.
HRMS (ESI) m/z: Calcd for $C_{15}H_{11}FN$ $[M+H]^+$: 224.0870. Found: 224.0875.
3-(2-Chlorophenyl)isoquinoline (3e). Yield: 2.13 g (86%), white solid, mp: 45 – 46 °C.
^{1}H NMR (500 MHz, $CDCl_3$): *δ* 9.36 (s, 1H), 8.05 – 8.00 (m, 2H), 8.87 (d, *J* = 8.0Hz, 1H), 7.76 – 7.68 (m, 2H), 7.66 (m, 1H), 7.52 (dd, *J* = 8.0,1.5 Hz, 1H), 7.39 (td, *J* = 7.5,1.5 Hz 1H), 7.34 (td, *J* = 7.5,2.0 Hz 1H) ppm.
^{13}C NMR (125 MHz, $CDCl_3$): *δ* 152.2, 150.3, 139.3, 136.0, 132.6, 132.0, 130.8, 130.3, 129.4, 127.71, 127.67, 127.66, 127.11, 127.07, 121.4 ppm.
HRMS (ESI) m/z: Calcd for $C_{15}H_{11}ClN$ $[M+H]^+$: 240.0574. Found: 240.0575.
3-(4-Chlorophenyl)isoquinoline (3f). Yield: 2.04 g (85%), Light yellow solid, mp: 141 – 142 °C.
^{1}H NMR (500 MHz, $CDCl_3$): *δ* 9.32 (s, 1H), 8.07 (d, *J* = 8.5 Hz 2H), 8.03 (s, 1H), 7.98 (d, *J* = 8.0 Hz 1H), 7.86 (d, *J* = 8.0 Hz 1H), 7.67– 7.73 (m, 1H), 7.62– 7.56 (m, 1H), 7.47 (d, *J* = 8.5 Hz 2H)ppm.
^{13}C NMR (125 MHz, $CDCl_3$): *δ* 152.5, 150.0, 137.9, 136.7, 134.8, 131.0, 129.1, 128.4, 127.9, 127.8, 127.5, 127.1, 116.7 ppm.
HRMS (ESI) m/z: Calcd for $C_{15}H_{11}ClN$ $[M+H]^+$: 240.0574. Found: 240.0573.
3-(4-Fluorophenyl)isoquinoline (3g). Yield: 1.88 g (84%), yellow solid, mp: 107 – 108 °C.
^{1}H NMR (500 MHz, $CDCl_3$): *δ* 9.31 (s, 1H), 8.13 – 8.07 (m, 2H), 8.02 – 7.95 (m, 2H), 7.85(d, *J* = 8.0 Hz 1H), 7.72 – 7.66 (m, 1H), 7.62 – 7.56 (m, 1H), 7.19 (t, *J* = 9.0 Hz 2H) ppm.
^{13}C NMR (101 MHz, $CDCl_3$): *δ* 163.4 (d, *J* = 222.3 Hz), 152.5, 150.3, 136.8, 135.7 (d, *J*= 3.0 Hz), 130.9, 128.9 (d, *J*= 8.2 Hz), 127.7, 127.3, 127.0, 116.4, 115.8 (d, *J*= 21.8 Hz) ppm.
HRMS (ESI) m/z: Calcd for $C_{15}H_{11}FN$ $[M+H]^+$: 224.0870. Found: 224.0874.
3-(4-(Trifluoromethyl)phenyl)isoquinoline (3h). Yield: 2.21 g (81%), yellow solid, mp: 158 – 159 °C.
^{1}H NMR (500 MHz, $CDCl_3$) : *δ* 9.34 (s, 1H), 8.23 (d, *J* = 8.0 Hz, 2H), 8.08 (s, 1H), 7.99 (d, *J* = 8.0 Hz, 1H), 7.87 (d, *J* = 8.5 Hz, 1H), 7.76 – 7.70 (m, 3H), 7.64 – 7.58 (m, 1H) ppm.
^{13}C NMR (125 MHz, $CDCl_3$): *δ* 152.8, 149.6, 143.0, 136.6, 131.0, 130.4 (d, *J*= 45.4 Hz), 129.1(d, *J*=146.8 Hz), 128.2, 127.8, 127.7, 127.3, 127.2, 125.8 (q, *J*= 3.9 Hz), 117.4 ppm.
HRMS (ESI) m/z: Calcd for $C_{16}H_{11}F_3N$ $[M+H]^+$: 274.0838. Found: 274.0831.
3-(Pyridin-4-yl)isoquinoline (3i). Yield: 1.79 g (87%), yellow solid, mp: 127 –128 °C.
^{1}H NMR (500 MHz, $CDCl_3$): *δ* 9.32 (s, 1H), 8.74 – 8.69 (m, 2H), 8.12 (s, 1H), 8.02 – 7.95 (m, 3H), 7.87 (d, *J*= 8.0 Hz 1H), 7.74 – 7.68 (m, 1H), 7.65 – 7.60 (m, 1H) ppm.
^{13}C NMR (125 MHz, $CDCl_3$): *δ* 152.9, 150.4, 148.3, 146.8, 136.3, 131.0, 128.6, 128.2, 127.7, 127.3, 121.2, 117.8 ppm.
HRMS (ESI) m/z: Calcd for $C_{14}H_{11}N_2$ $[M+H]^+$: 207.0917. Found: 207.0915.
3-(2-Methoxyphenyl)isoquinoline (3j). Yield: 2.19 g (93%), yellow solid, mp: 129 – 130 °C.
^{1}H NMR (500 MHz, $CDCl_3$): *δ* 9.36 (s, 1H), 8.22 (s, 1H), 8.00 – 7.92 (m, 2H), 7.84 (d, *J*= 8.5 Hz, 1H), 7.70 – 7.63 (m, 1H), 7.60 – 7.52 (m, 1H), 7.43 – 7.36 (m, 1H), 7.18 – 7.12 (m, 1H), 7.04 (d, *J*= 8.5 Hz 1H), 3.89 (s, 3H) ppm.

^{13}C NMR (125 MHz, $CDCl_3$): δ 157.1, 151. 9, 149.2, 136.2, 131.5, 130.3, 129.6, 129.1, 127.43, 127.36, 127.03, 127.01, 121.2, 121.1, 111.4, 55.7 ppm.

HRMS (ESI) m/z: Calcd for $C_{16}H_{14}NO$ $[M+H]^+$: 236.1070. Found: 236.1061.

3-(4-Methoxyphenyl)isoquinoline (3k). Yield: 2.21 g (94%), yellow solid, mp: 100 – 101 °C.

^1H NMR (500 MHz, $CDCl_3$): δ 9.31 (s, 1H), 8.08 (d, *J*= 9.0 Hz 2H), 7.98 (s, 1H), 7.96 (d, *J* = 8.5, 1H), 7.83 (d, *J* = 8.5, 1H), 7.69 – 7.64 (m, 1H), 7.57 – 7.52 (m, 1H), 7.04 (d, *J* = 8.5 2H), 3.88 (s, 3H) ppm.

^{13}C NMR (125 MHz, $CDCl_3$): δ 160.3, 152.3, 151.0, 137.0, 132.1, 130.7, 128.4, 127.8, 127.5, 126.9, 115.6, 114.3, 55.5 ppm.

HRMS (ESI) m/z: Calcd for $C_{16}H_{14}NO$ $[M+H]^+$: 236.1070. Found: 236.1066.

3-(4-(*tert*-Butyl)phenyl)isoquinoline (3l). Yield: 2.38 g (91%), yellow solid, mp: 74 – 75 °C.

^1H NMR (500 MHz, $CDCl_3$): δ 9.35 (s, 1H), 8.10 (d, *J* = 8.5, 2H), 8.05 (s, 1H), 7.97 (d, *J* = 8.0 Hz, 1H), 7.85 (d, *J* = 8.0 Hz, 1H), 7.69 – 7.64 (m, 1H), 7.59 – 7.52 (m, 3H), 1.42 (s, 9H) ppm.

^{13}CNMR (125 MHz, $CDCl_3$): δ 152.4, 151.7, 151.3, 136.82, 136.77, 130.5, 127.72, 127.66, 127.0, 126.8, 125.9, 116.2, 34.8, 31.4 ppm.

HRMS (ESI) m/z: Calcd for $C_{19}H_{20}N$ $[M+H]^+$: 262.1590. Found: 262.1596.

6-Methoxy-3-(4-methoxyphenyl)isoquinoline (3m). Yield: 2.55 g (96%), yellow solid, mp: 113 –114 °C.

^1H NMR (500 MHz, $CDCl_3$): δ 9.16 (s, 1H), 8.08 (d, *J* = 9.0 Hz, 2H), 7.89 (s, 1H), 7.83 (d, *J* = 8.5 Hz, 1H), 7.21 (d, *J* = 8.0 Hz, 1H), 7.08 (d, *J* = 8.5 Hz, 1H), 7.04 (d, *J* = 8.5 Hz, 2H), 3.95 (s, 3H), 3.88 (s, 3H) ppm.

^{13}C NMR (125 MHz, $CDCl_3$): δ 161.3, 160.3, 151.0, 139.1, 132.1, 130.7, 128.5, 128.3, 123.3, 120.4, 115.6, 114.3, 104.3, 55.5, 55.0 ppm.

HRMS (ESI) m/z: calcd for $C_{17}H_{16}NO_2$ $[M + H]^+$: 266.1176. found, 266.1168.

3-(4-Phenoxyphenyl)isoquinoline (3p). Yield: 2.76 g (93%), brown solid, mp: 106 – 107 °C.

^1H NMR (500 MHz, $CDCl_3$): δ 9.33 (s, 1H), 8.12 (d, *J* = 9.0 Hz 2H), 8.02 (s, 1H), 7.97 (dd, *J* = 8.0,1.0 Hz, 1H), 7.85 (dd, *J* = 8.0,1.0 Hz, 1H), 7.70 – 7.66 (m, 1H), 7.60 – 7.55 (m, 1H), 7.41 – 7.35 (m, 2H), 7.18 – 7.12 (m, 3H), 7.12 – 7.08 (m, 2H) ppm.

^{13}C NMR (125 MHz, $CDCl_3$): δ 157.9, 157.1, 152.5, 150.8, 136.8, 134.8, 130.7, 129.9, 128.6, 127.69, 127.66, 127.1, 126.9, 123.6, 119.2, 119.1, 116.0 ppm.

HRMS (ESI) m/z: Calcd for $C_{21}H_{16}NO$ $[M+H]^+$: 298.1226. Found: 298.1221.

3-([1,1'-Biphenyl]-4-yl)isoquinoline (3o). Yield: 2.50 g (89%), yellow solid, mp: 97 – 99 °C.

^1H NMR (500 MHz, $CDCl_3$): δ 9.37 (s, 1H), 8.24 (d, *J* = 8.0,1.0 Hz, 2H), 8.11 (s, 1H), 7.99 (d, *J* = 8.5 Hz, 1H), 7.86(d, *J* = 8.5 Hz, 1H), 7.77 (d, *J* = 8.0, 2H), 7.73 – 7.65 (m, 3H), 7.58 (t, *J* = 8.0 1H), 7.49 (t, *J* = 7.5 Hz, 2H), 7.39 (t, *J* = 7.5 Hz, 1H) ppm.

^{13}C NMR (125 MHz, $CDCl_3$): δ 152.5, 150.8, 141.4, 140.8, 138.5, 136.8, 130.7, 128.9, 127.9, 127.7, 127.62, 127.58, 127.5, 127.24, 127.20, 127.0, 116.6 ppm.

HRMS (ESI) m/z: Calcd for $C_{21}H_{16}N$ $[M+H]^+$: 282.1277. Found: 282.1272.

4-(Isoquinolin-3-yl)-*N,N*-dimethylaniline (3af). Yield: 2.33 g (94%), Light yellow solid, mp: 138 – 139 °C.

^1H NMR (500 MHz, $CDCl_3$): δ 9.29 (s, 1H), 8.06 (d, *J* = 9.0 Hz, 2H), 7.97 – 7.92 (m, 2H), 7.81 (dd, *J* = 8.5, 1.0 Hz, 1H), 7.66 – 7.62 (m, 1H), 7.52 – 7.48 (m, 1H), 6.85 (d, *J* = 9.0 Hz 2H), 3.04 (s, 6H) ppm.

^{13}C NMR (125 MHz, $CDCl_3$); δ 152.1, 151.5, 150.9, 137.1, 130.6, 127.9, 127.7, 127.3, 127.2, 126.8, 126.4, 114.5, 112.6, 40.6 ppm.

HRMS (ESI) m/z: Calcd for $C_{17}H_{17}N_2$ $[M+H]^+$: 249.1386. Found: 249.1382.

NMR spectra

The ^1H and ^{13}C NMR spectra of all synthesized compounds are provided below.

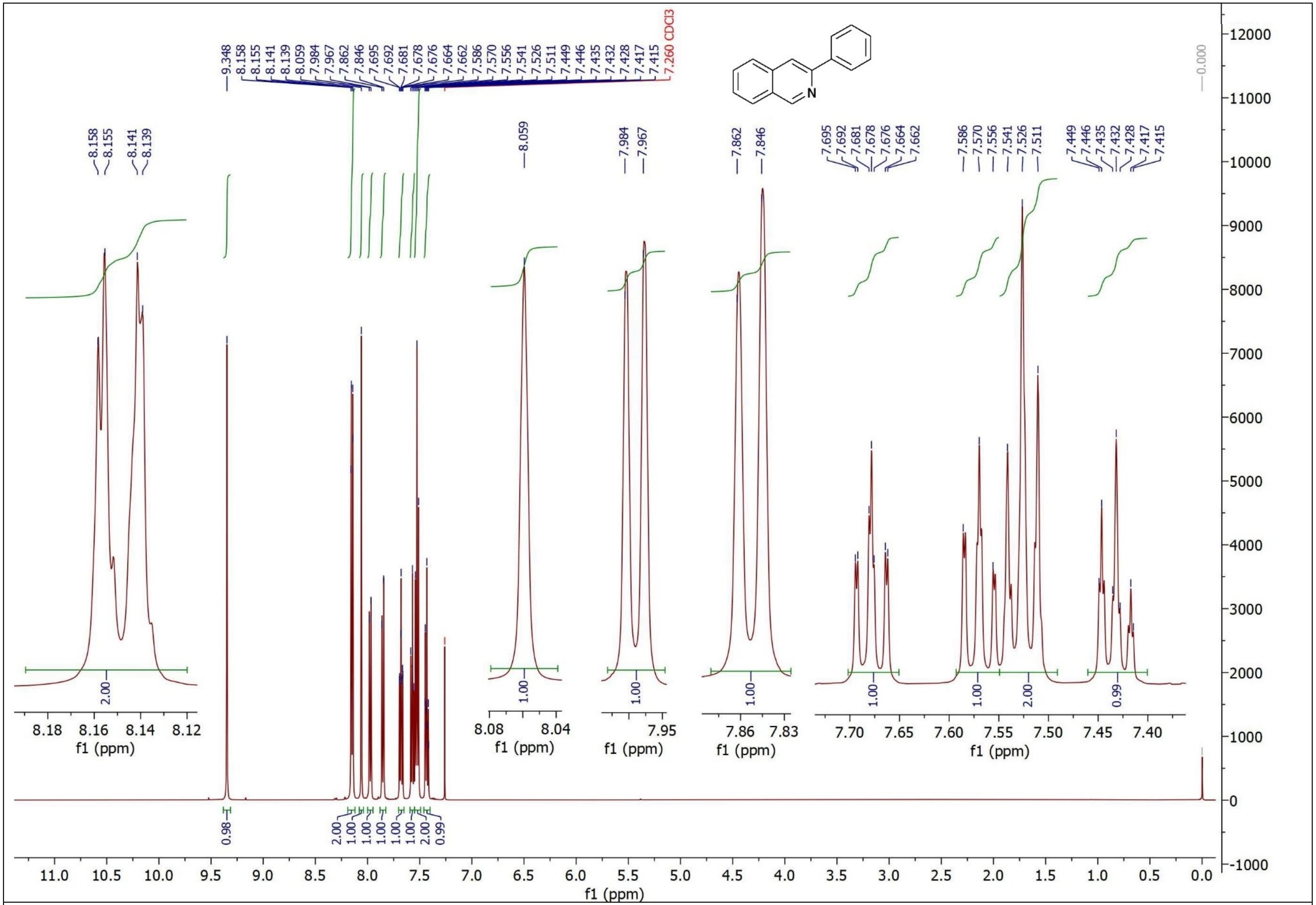

^{1}H NMR (500 MHz, $CDCl_3$): **3-Phenylisoquinoline (3a)**

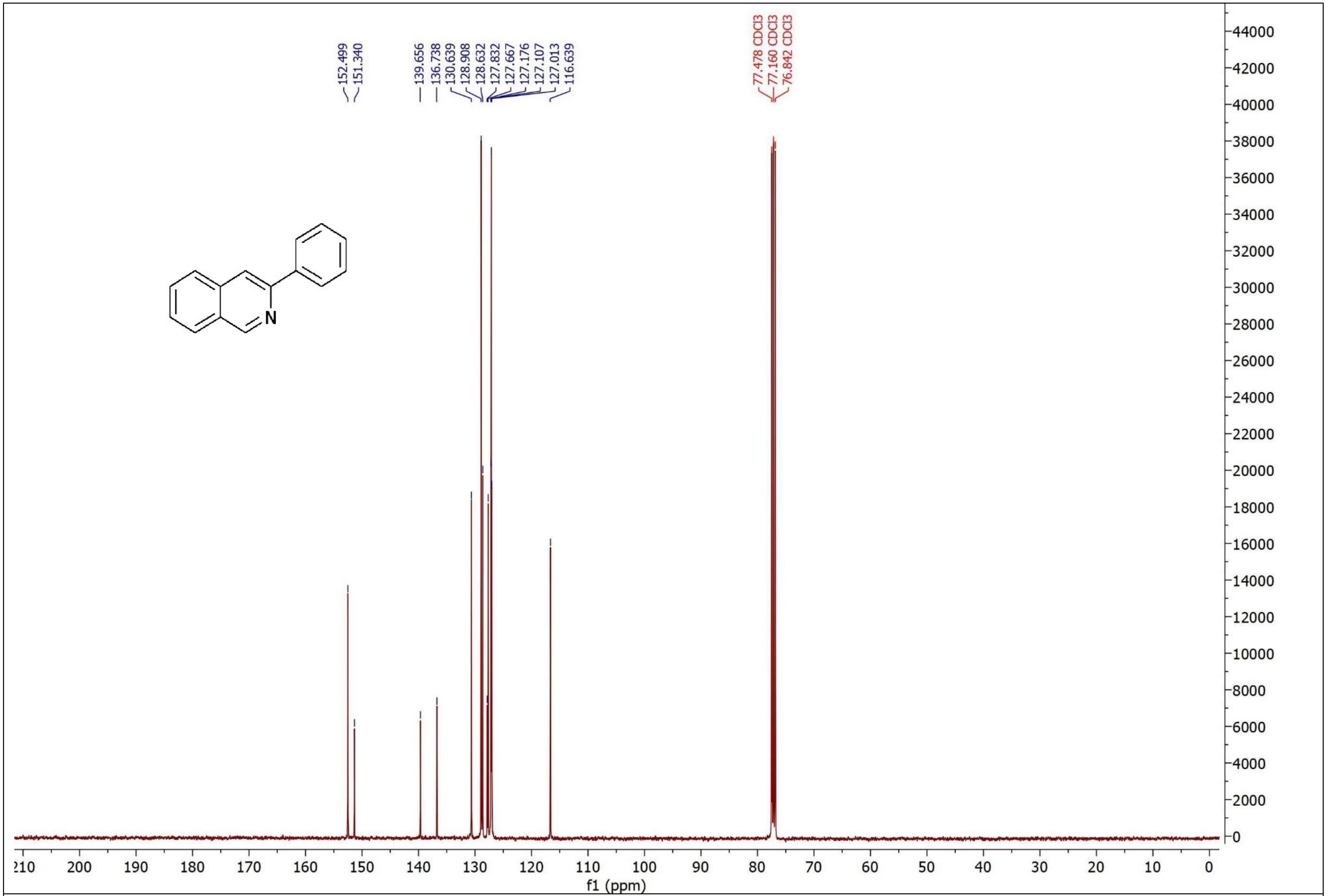

^{13}C NMR (125 MHz, $CDCl_3$): **3-Phenylisoquinoline (3a)**

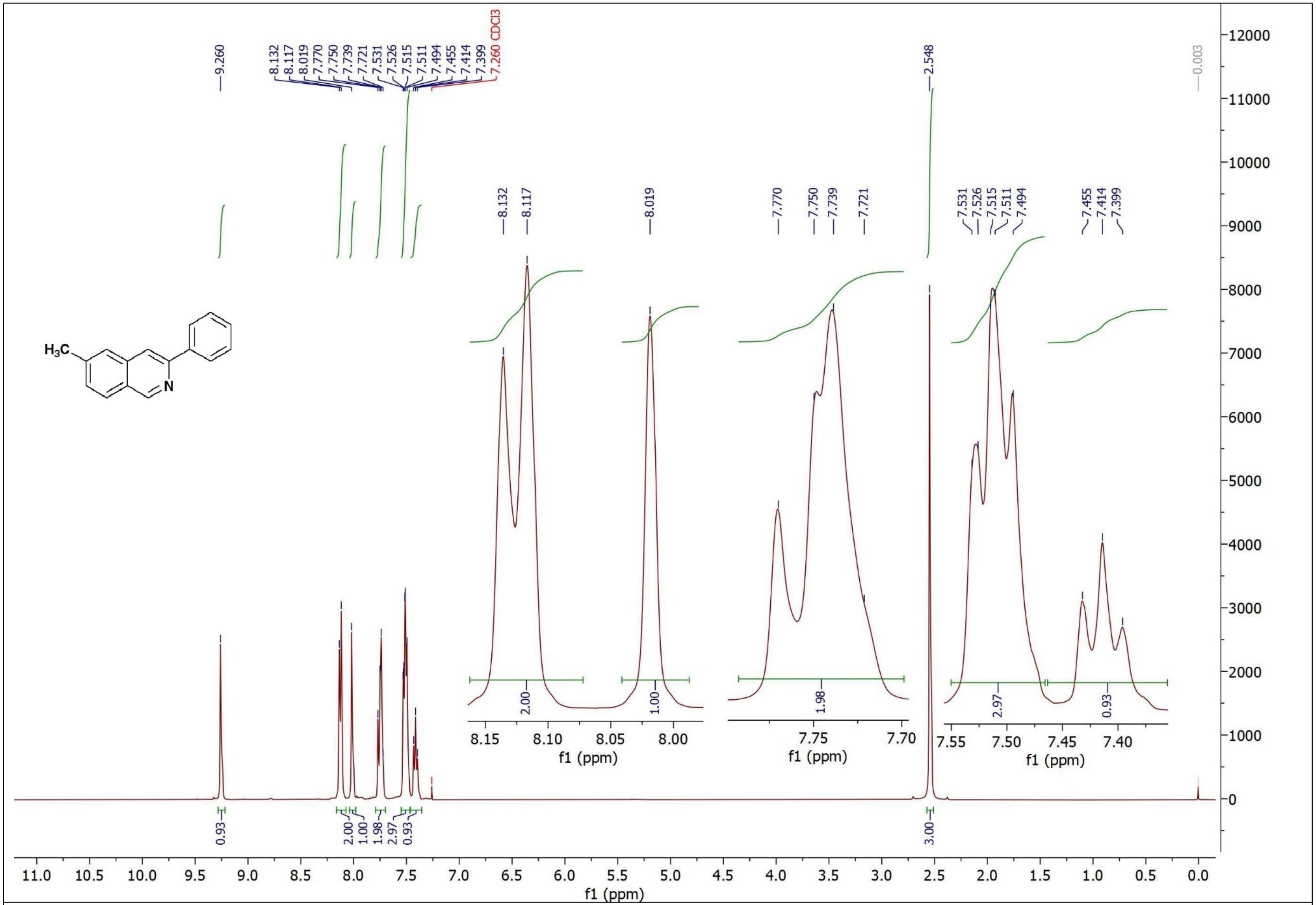

^{1}H NMR (500 MHz, $CDCl_3$): **6-Methyl-3-phenylisoquinoline (3b)**

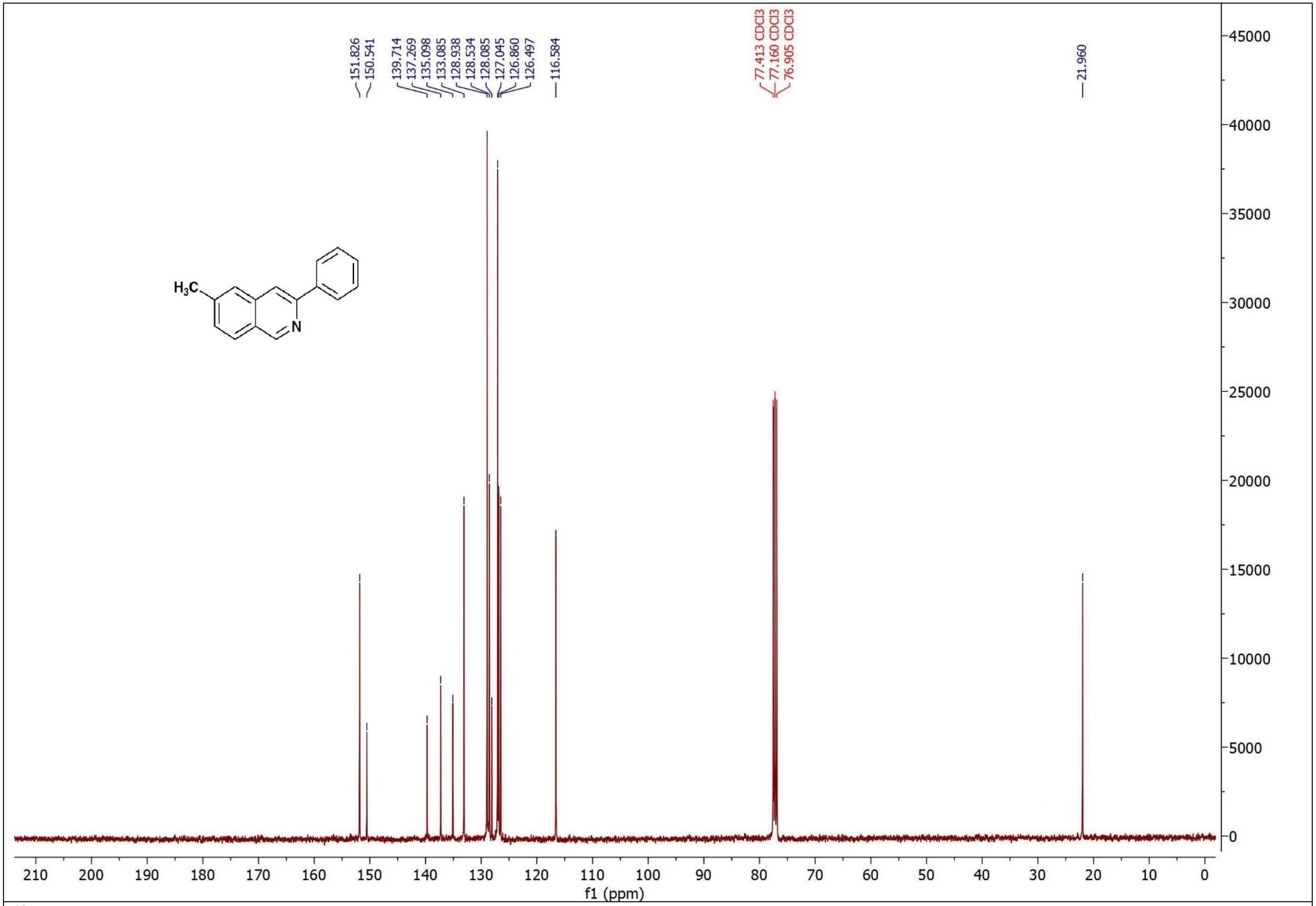

^{13}C NMR (125 MHz, $CDCl_3$): **6-Methyl-3-phenylisoquinoline (3b)**

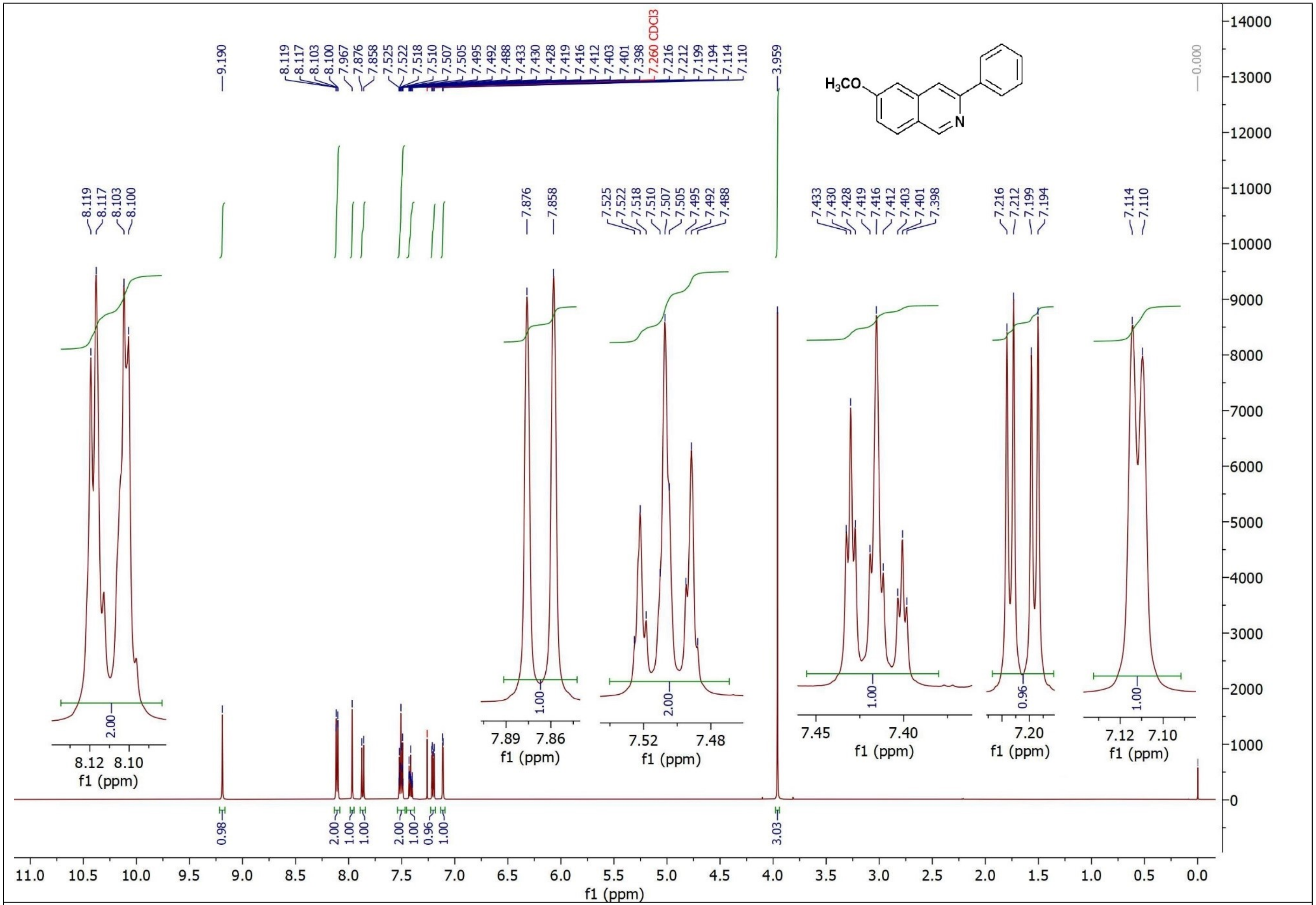

^{1}H NMR (500 MHz, $CDCl_3$): **6-Methoxy-3-phenylisoquinoline (3c)**

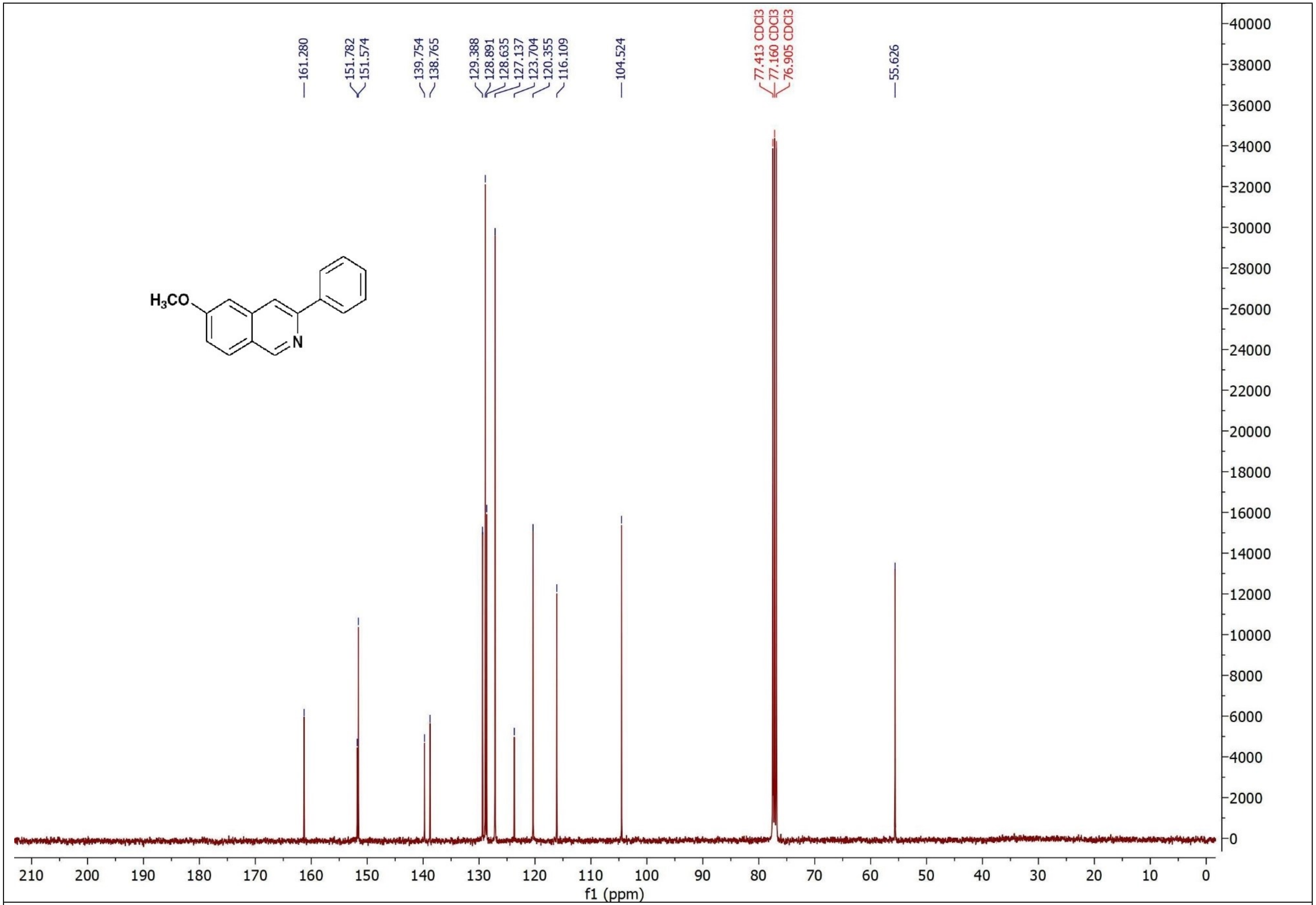

^{13}C NMR (125 MHz, $CDCl_3$): **6-Methoxy-3-phenylisoquinoline (3c)**

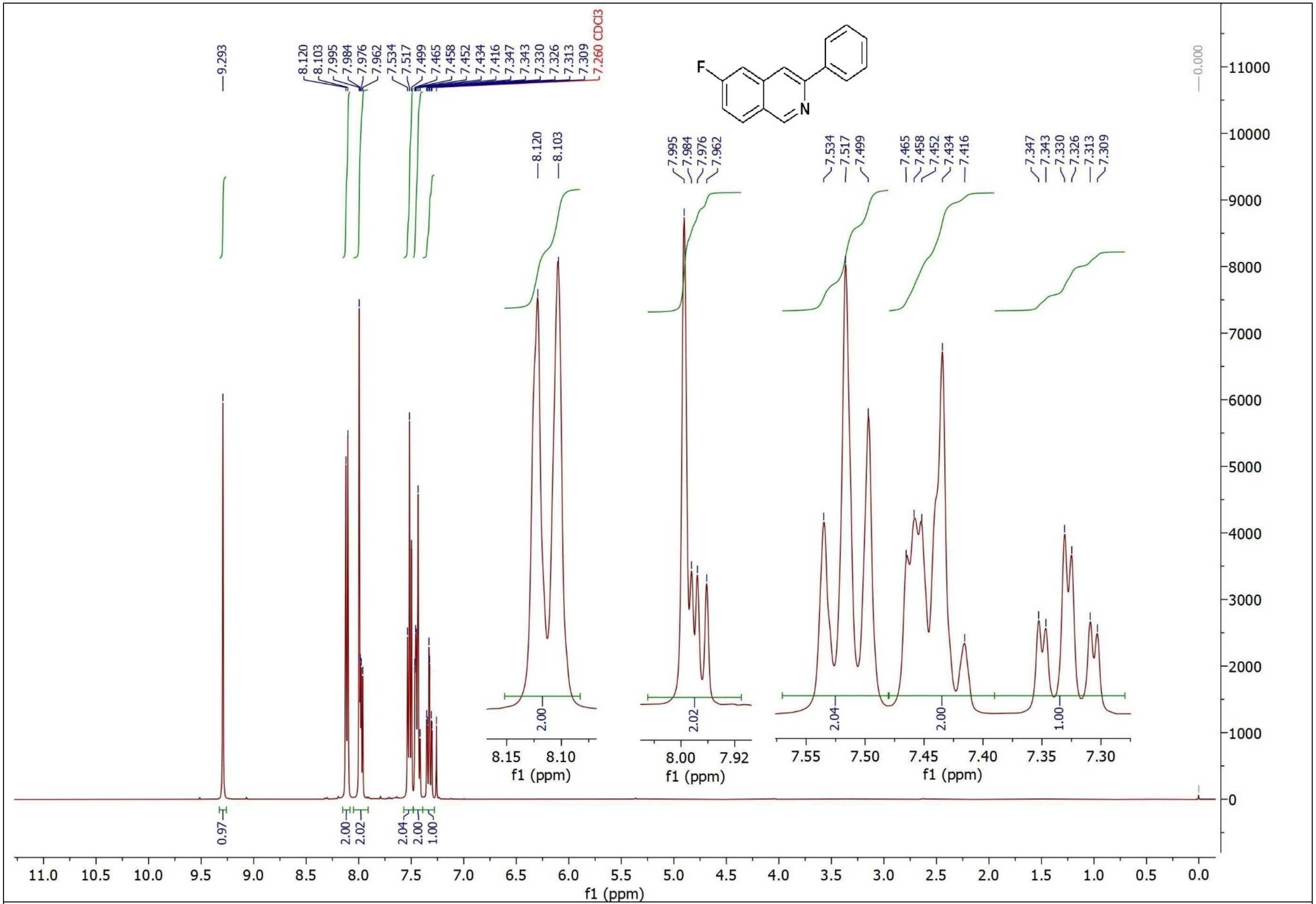

^{1}H NMR (500 MHz, $CDCl_3$): **6-Fluoro-3-phenylisoquinoline (3d)**

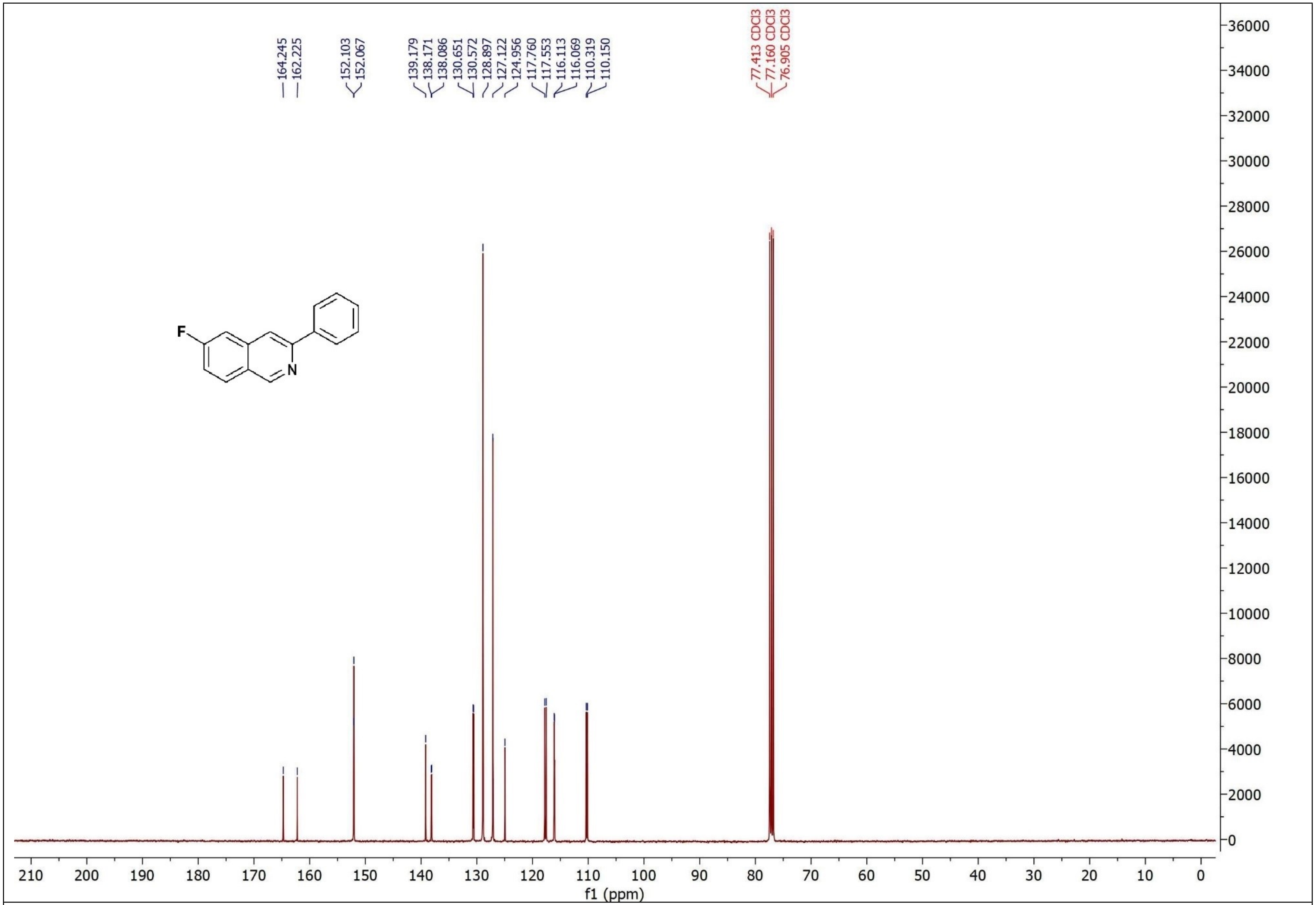

^{13}C NMR (125 MHz, $CDCl_3$): **6-Fluoro-3-phenylisoquinoline (3d)**

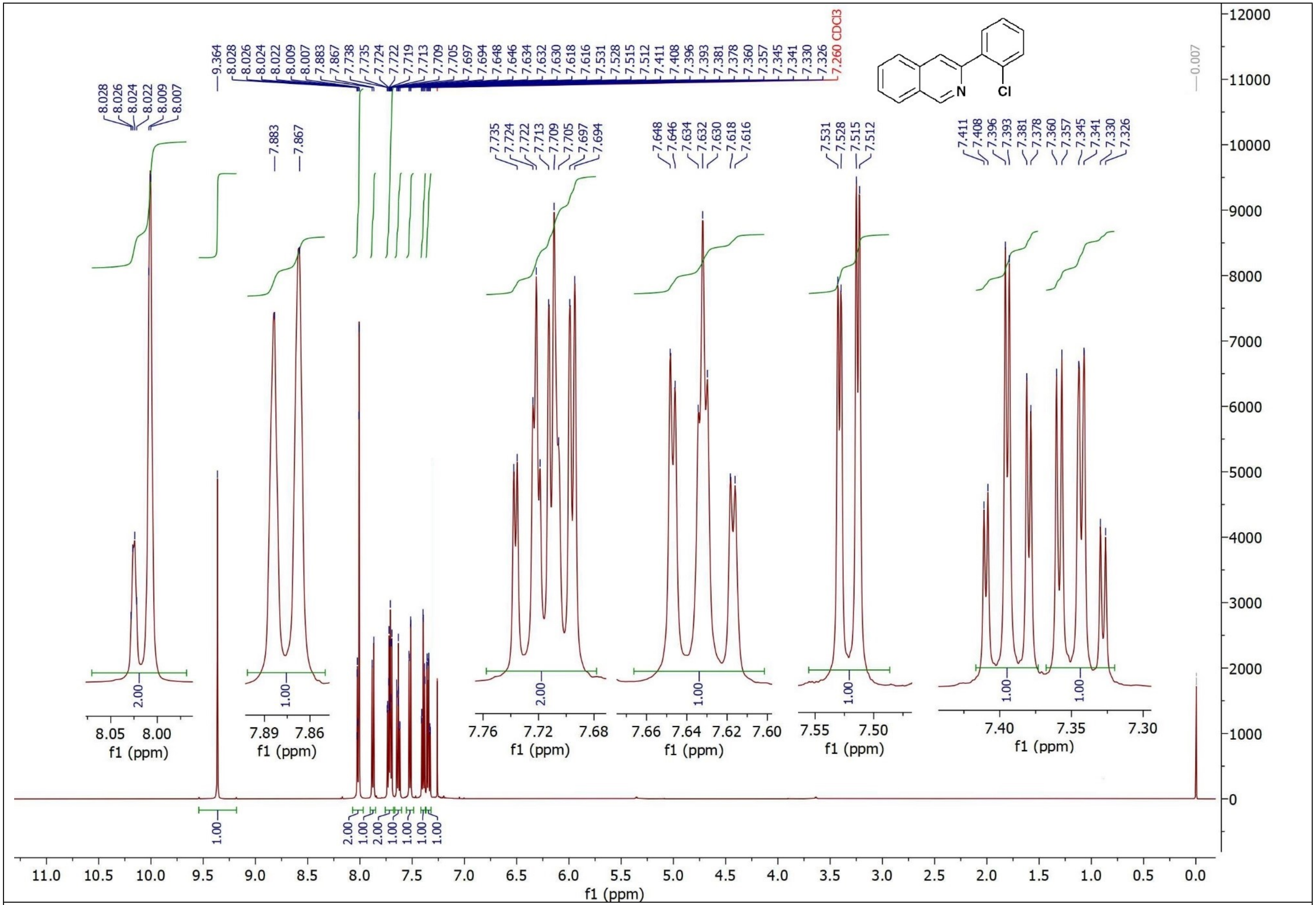

^{1}H NMR (500 MHz, $CDCl_3$): **3-(2-Chlorophenyl)isoquinoline (3e)**

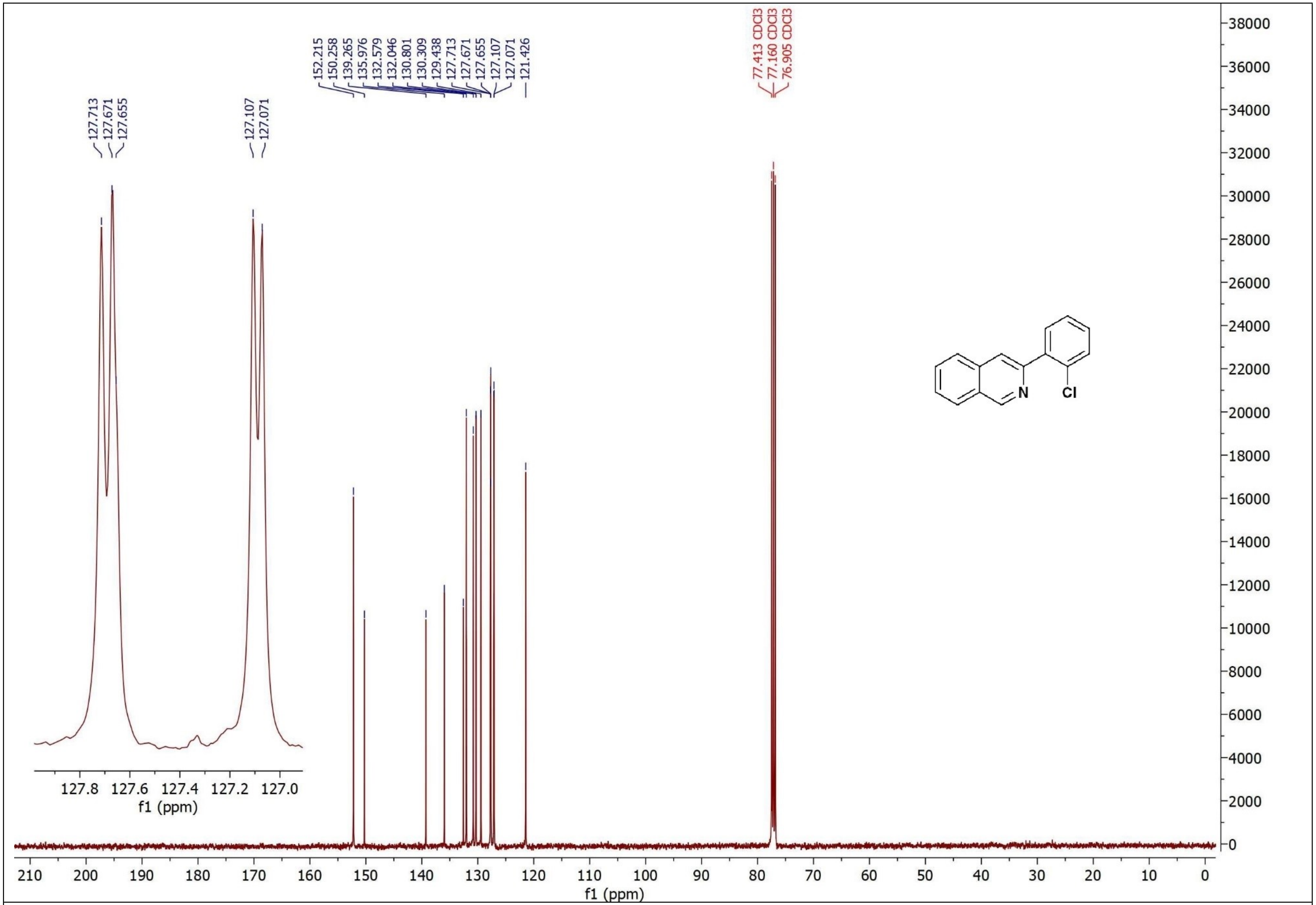

^{13}C NMR (125 MHz, $CDCl_3$): **3-(2-Chlorophenyl)isoquinoline (3e)**

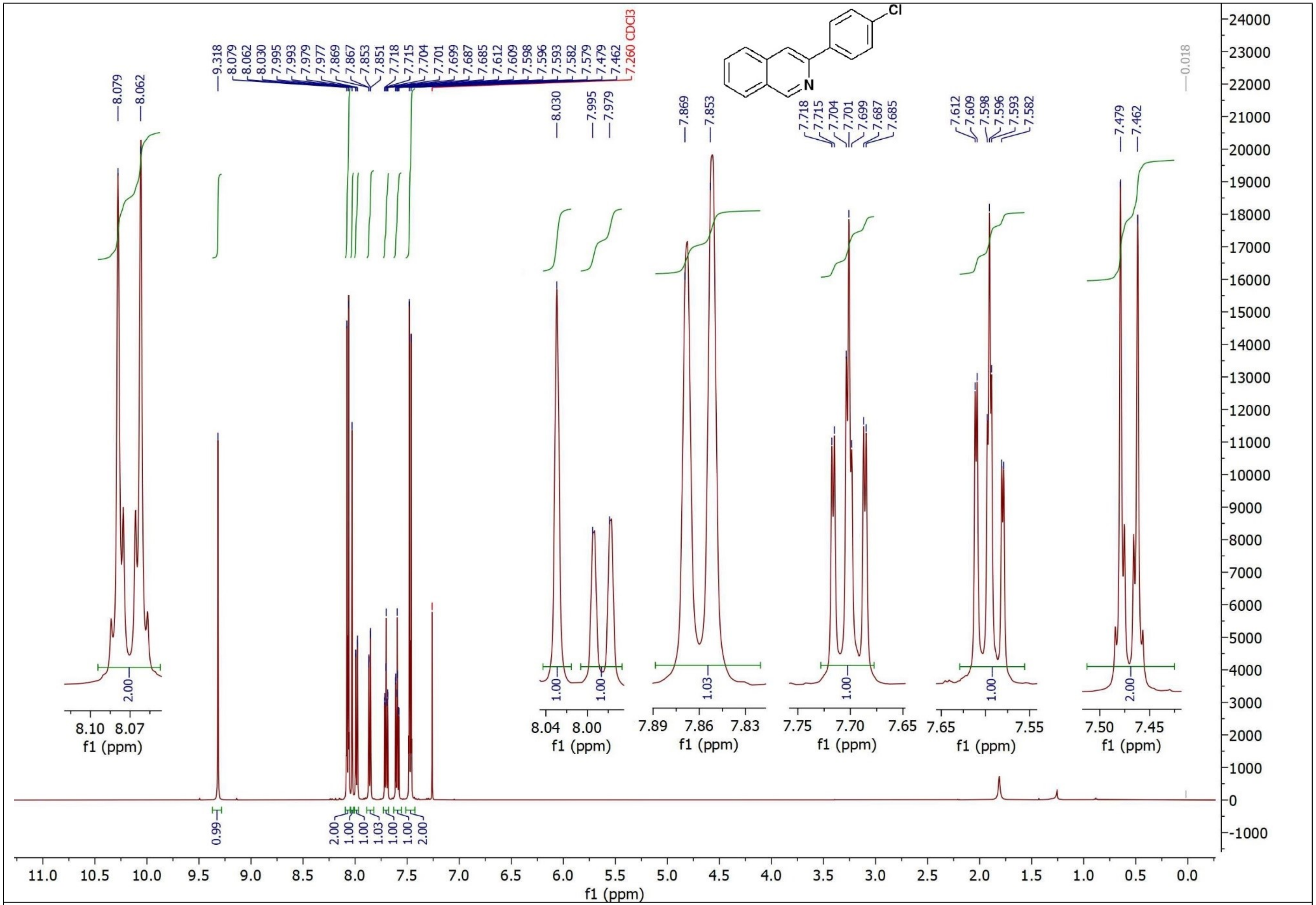

^{1}H NMR (500 MHz, $CDCl_3$): **3-(4-Chlorophenyl)isoquinoline (3f)**

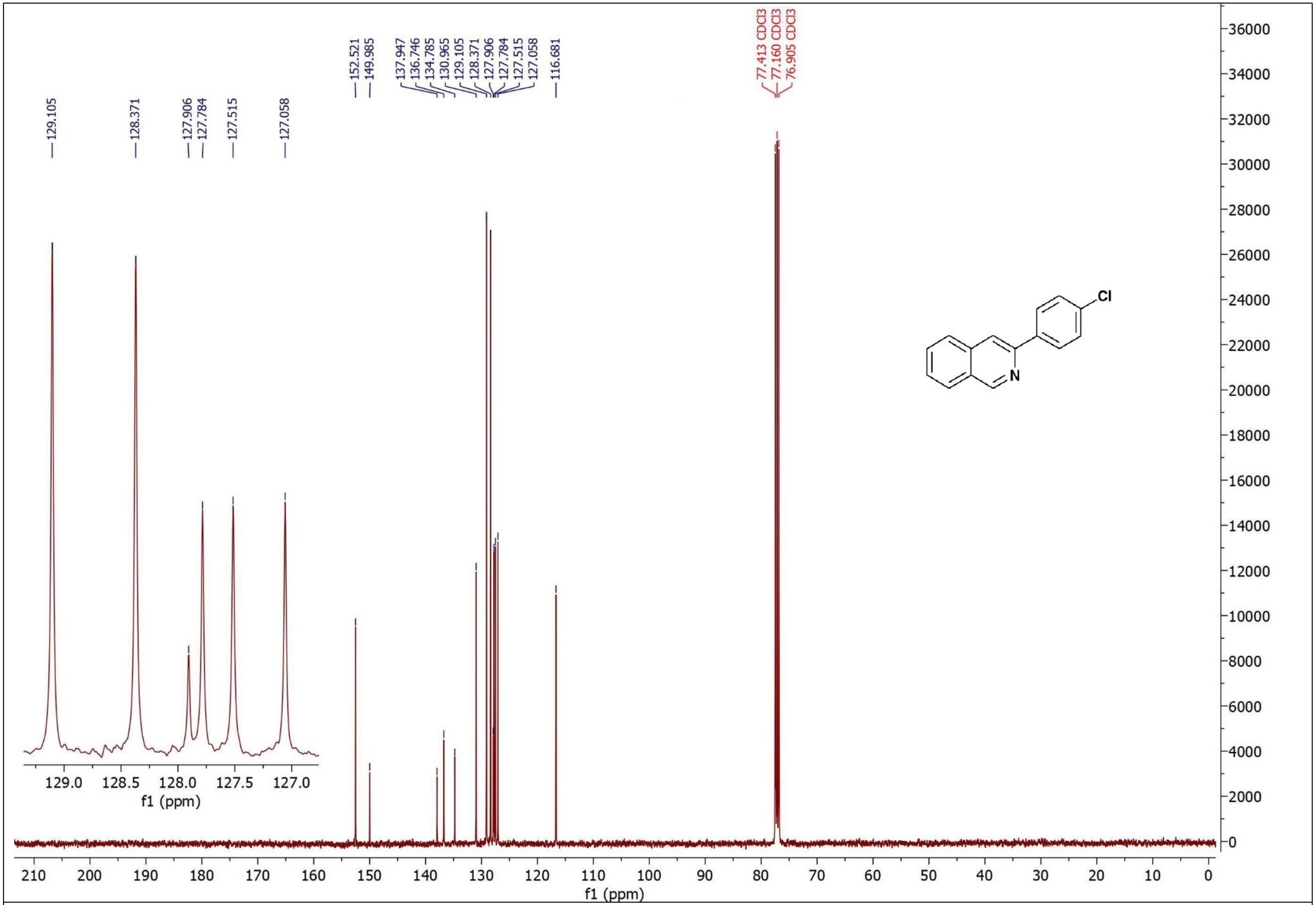

^{13}C NMR (125 MHz, $CDCl_3$): **3-(4-Chlorophenyl)isoquinoline (3f)**

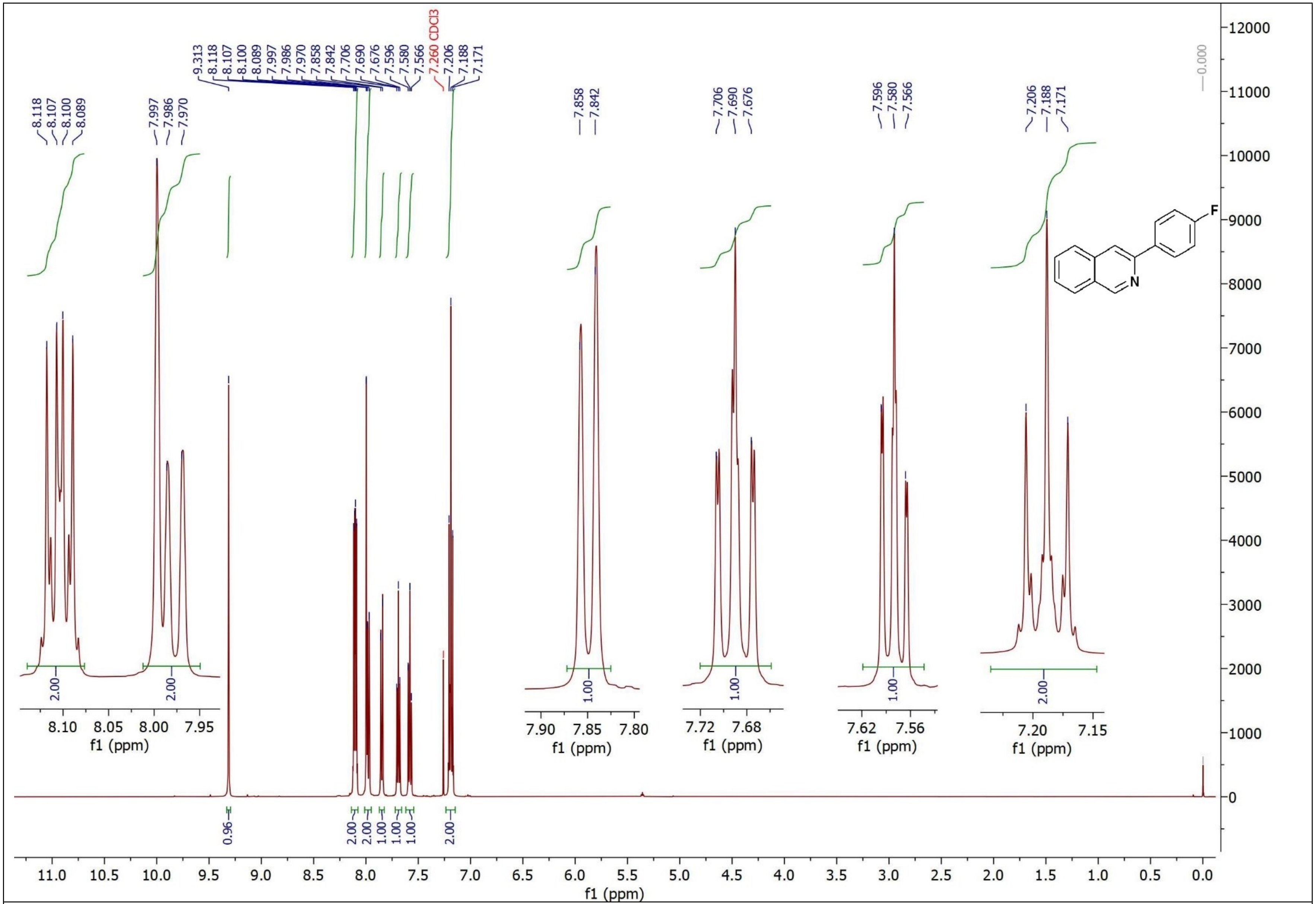

^{1}H NMR (500 MHz, $CDCl_3$): **3-(4-Fluorophenyl)isoquinoline (3g)**

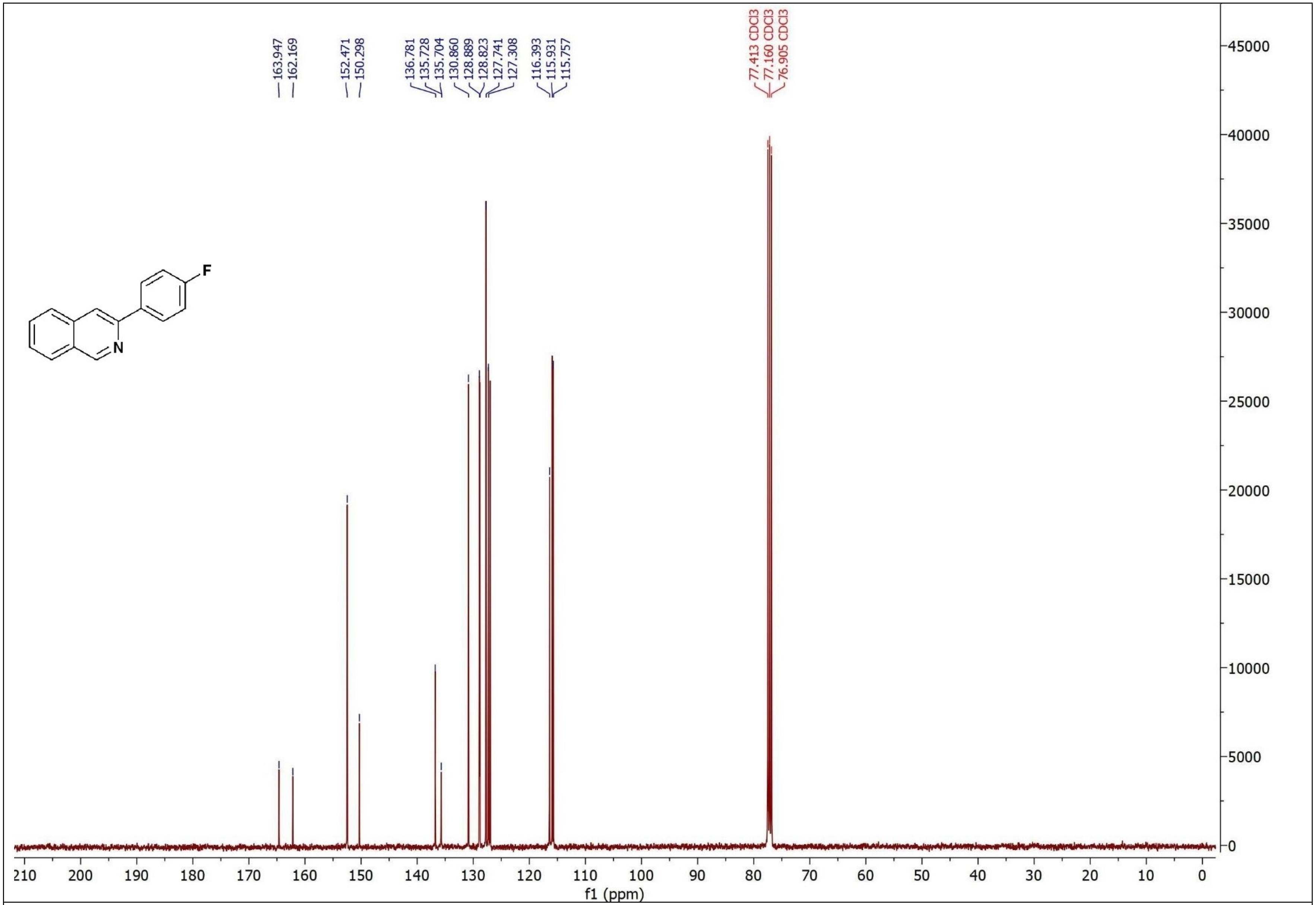

^{13}C NMR (125 MHz, $CDCl_3$): **3-(4-Fluorophenyl)isoquinoline (3g)**

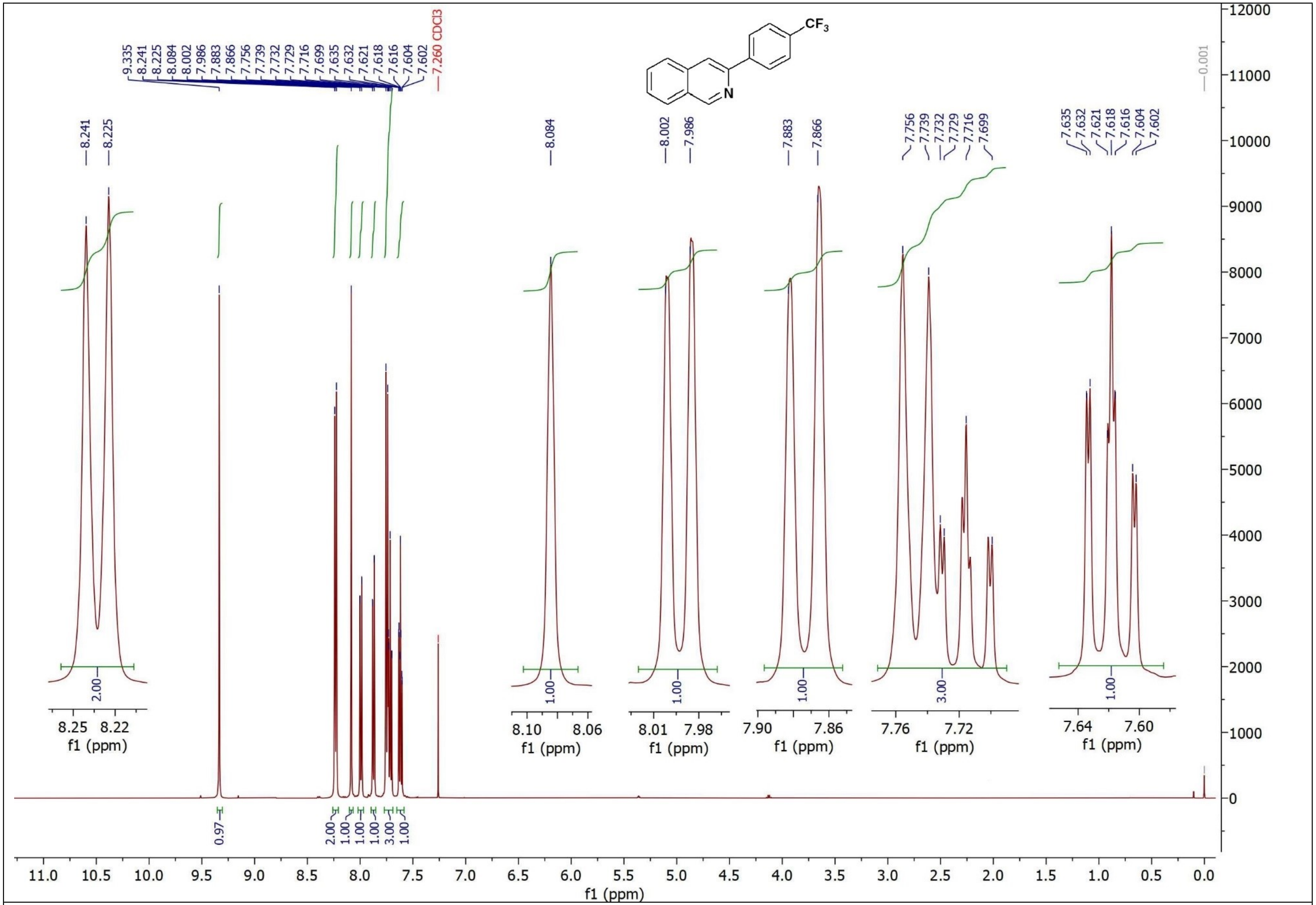

^{1}H NMR (500 MHz, $CDCl_3$): **3-(4-(Trifluoromethyl)phenyl)isoquinoline (3h)**

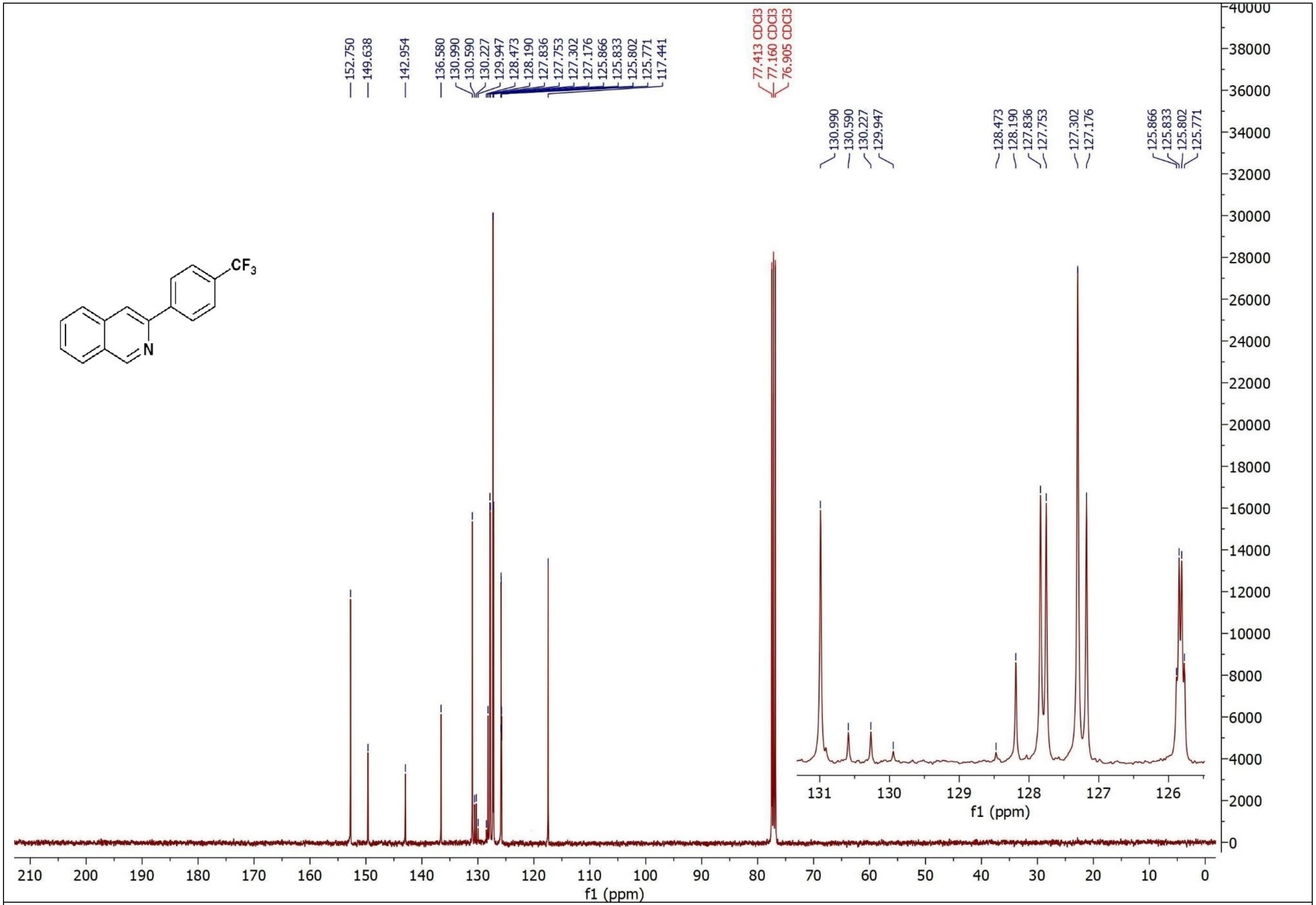

^{13}C NMR (125 MHz, $CDCl_3$): **3-(4-(Trifluoromethyl)phenyl)isoquinoline (3h)**

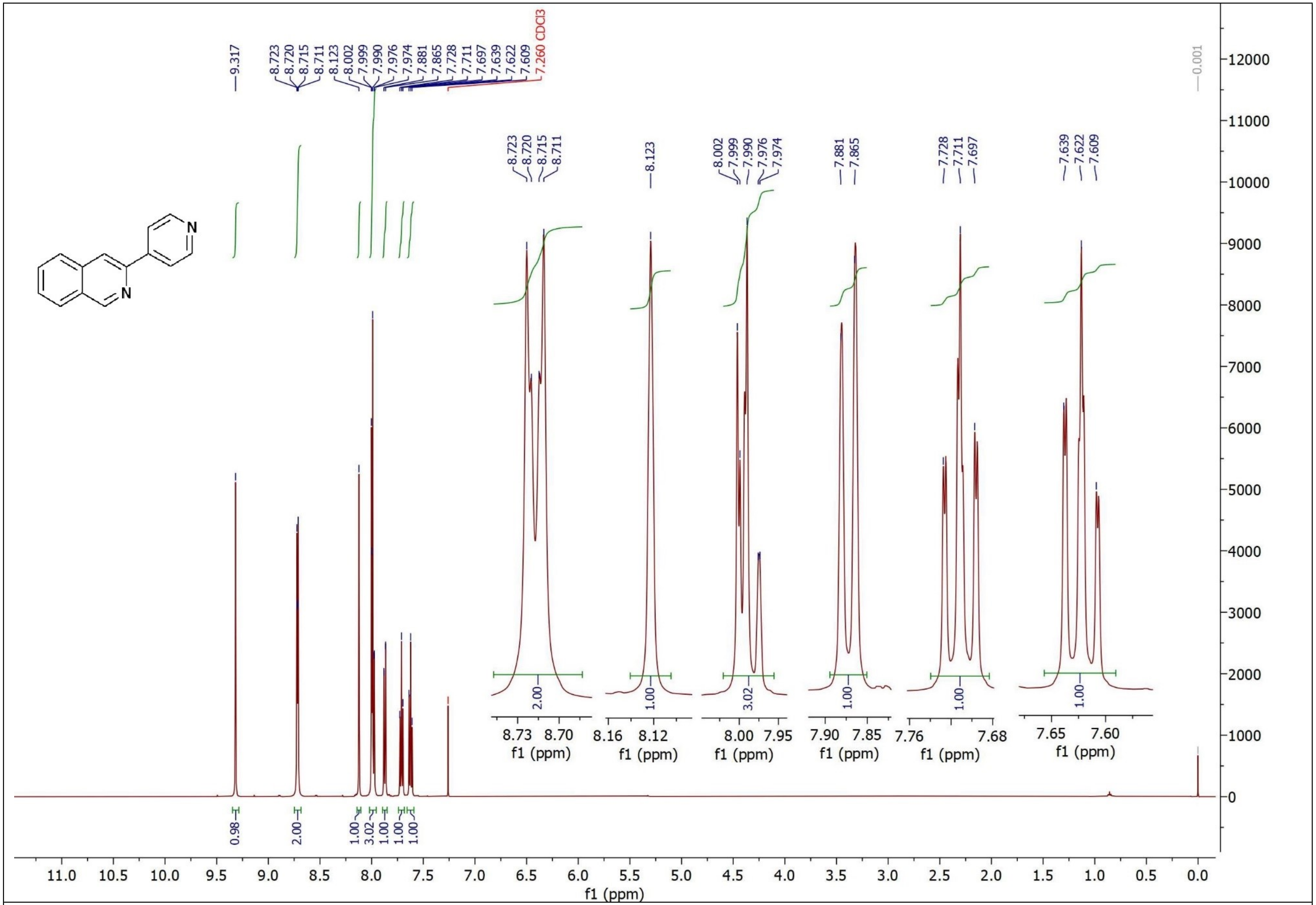

^{1}H NMR (500 MHz, $CDCl_3$): **3-(Pyridin-4-yl)isoquinoline (3i)**

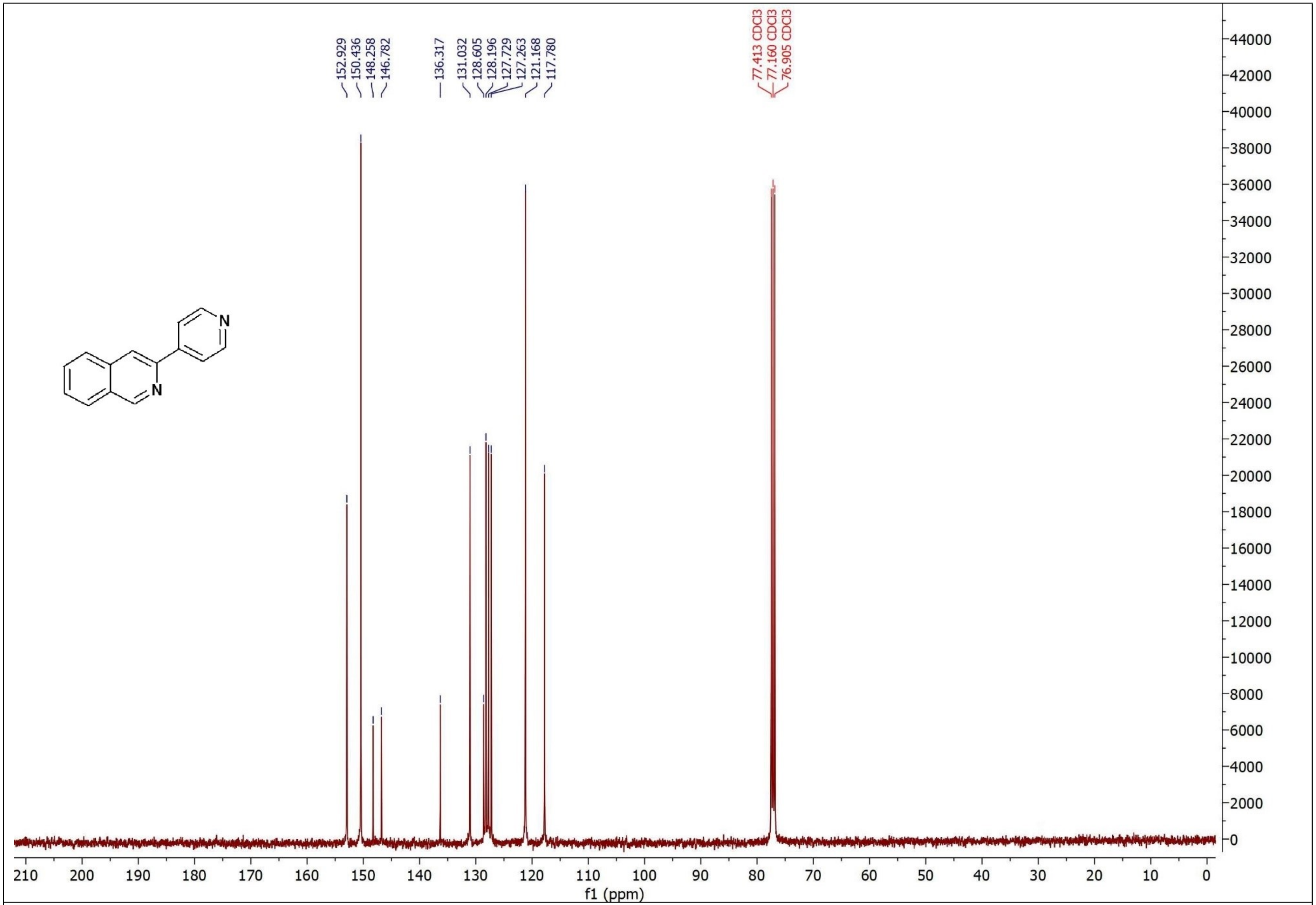

^{13}C NMR (125 MHz, $CDCl_3$): **3-(Pyridin-4-yl)isoquinoline (3i)**

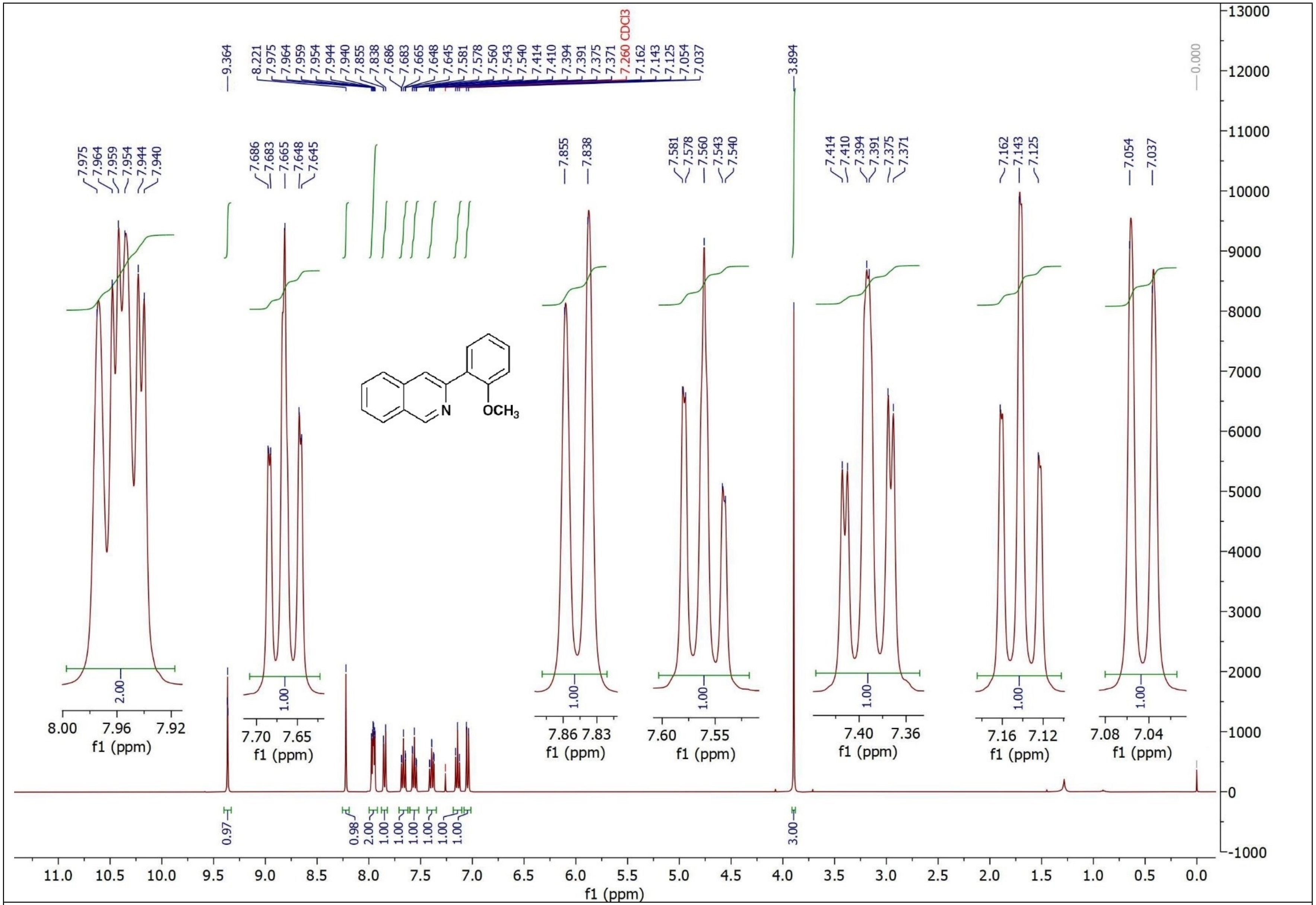

^{1}H NMR (500 MHz, $CDCl_3$): **3-(2-Methoxyphenyl)isoquinoline (3j)**

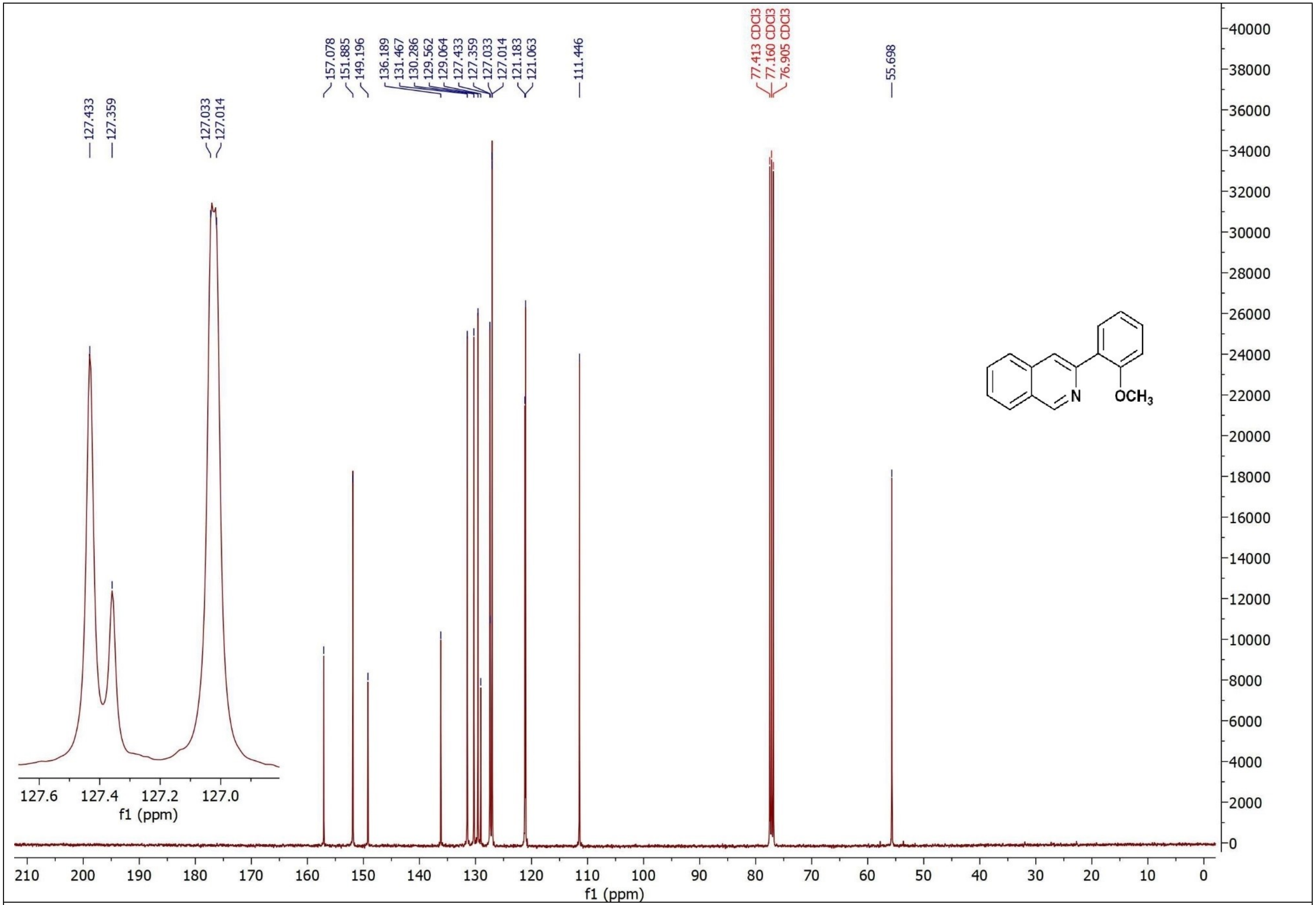

^{13}C NMR (125 MHz, $CDCl_3$): **3-(2-Methoxyphenyl)isoquinoline (3j)**

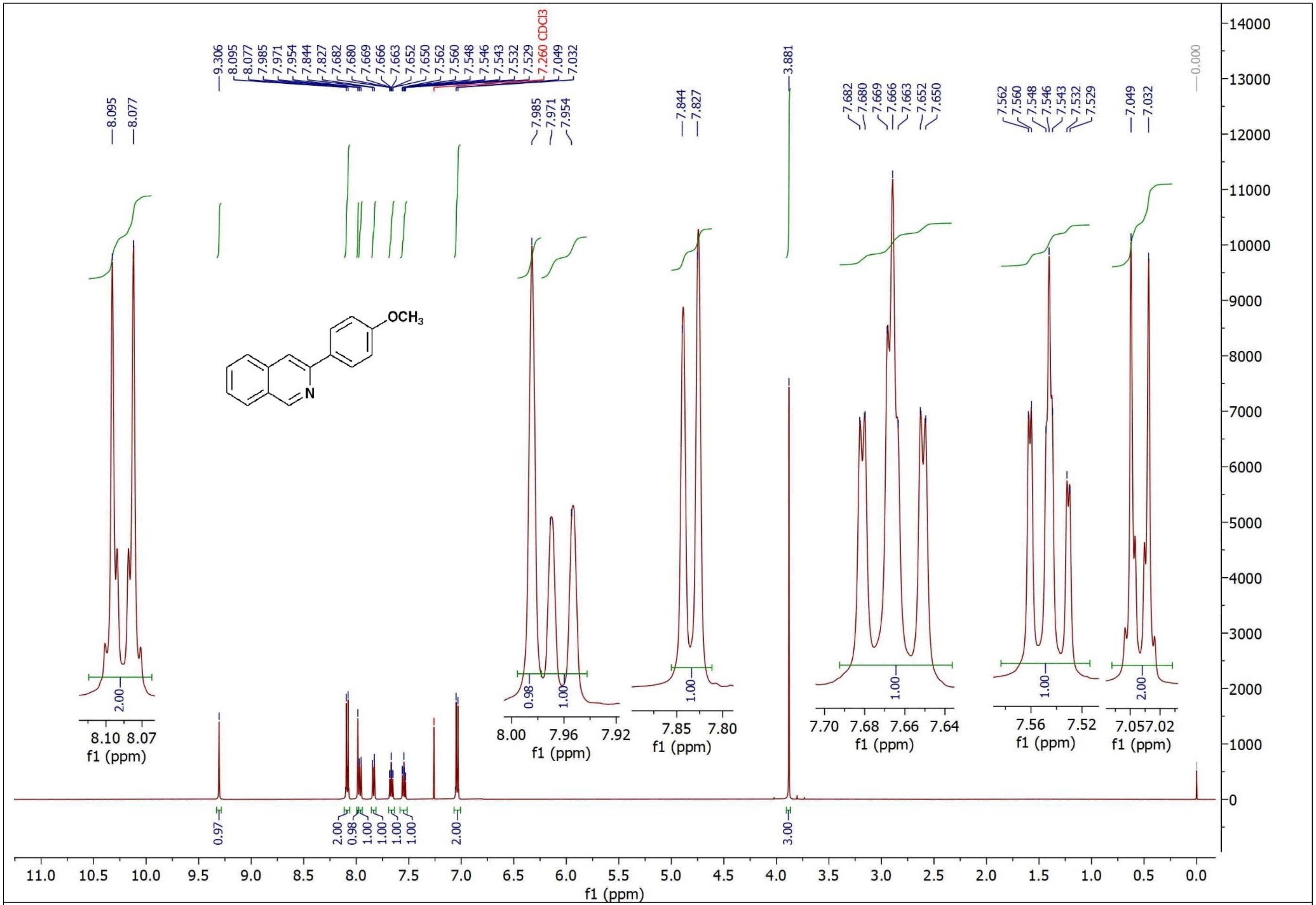

^{1}H NMR (500 MHz, $CDCl_3$): **3-(4-Methoxyphenyl)isoquinoline (3k)**

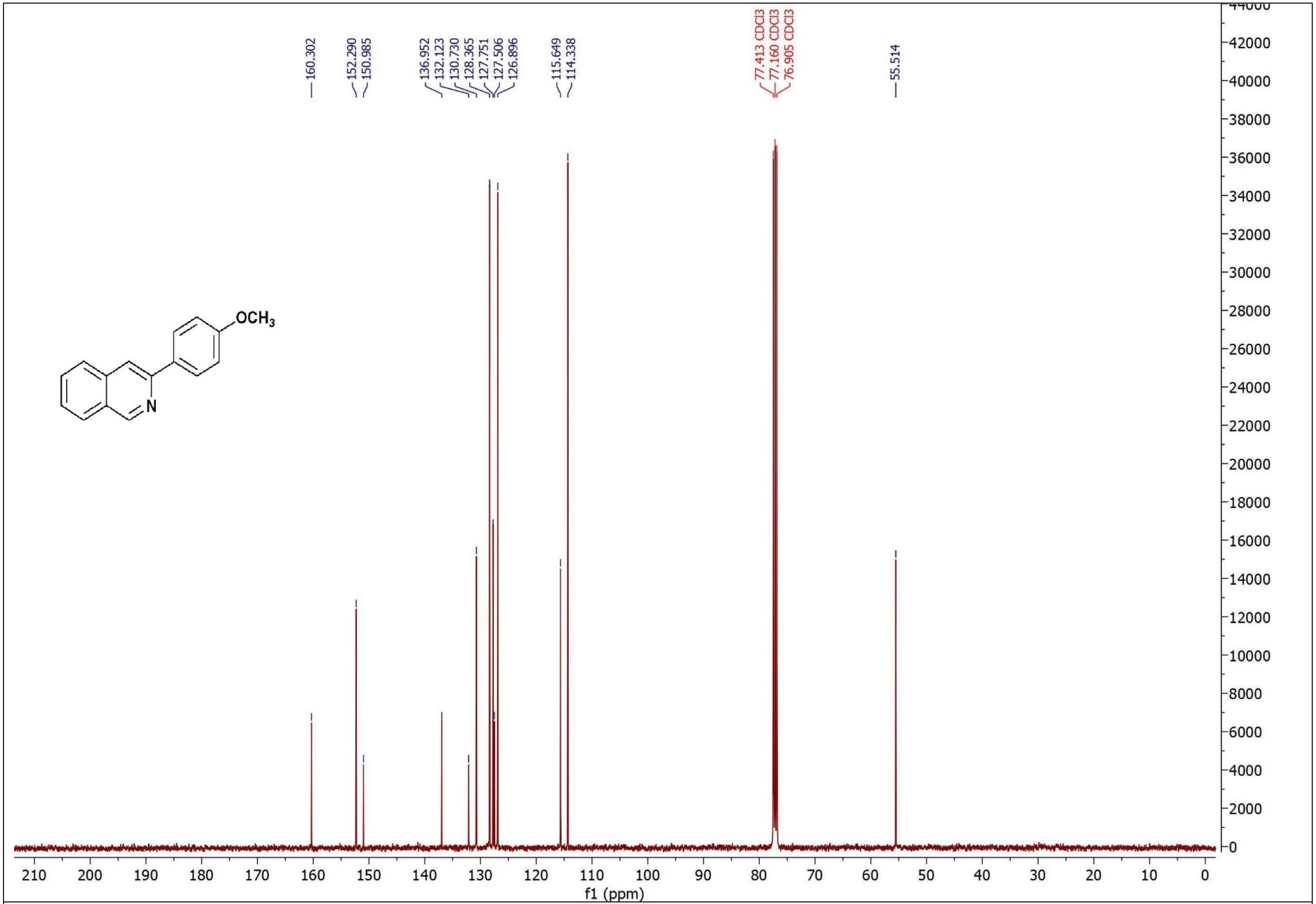

^{13}C NMR (125 MHz, $CDCl_3$): **3-(4-Methoxyphenyl)isoquinoline (3k)**

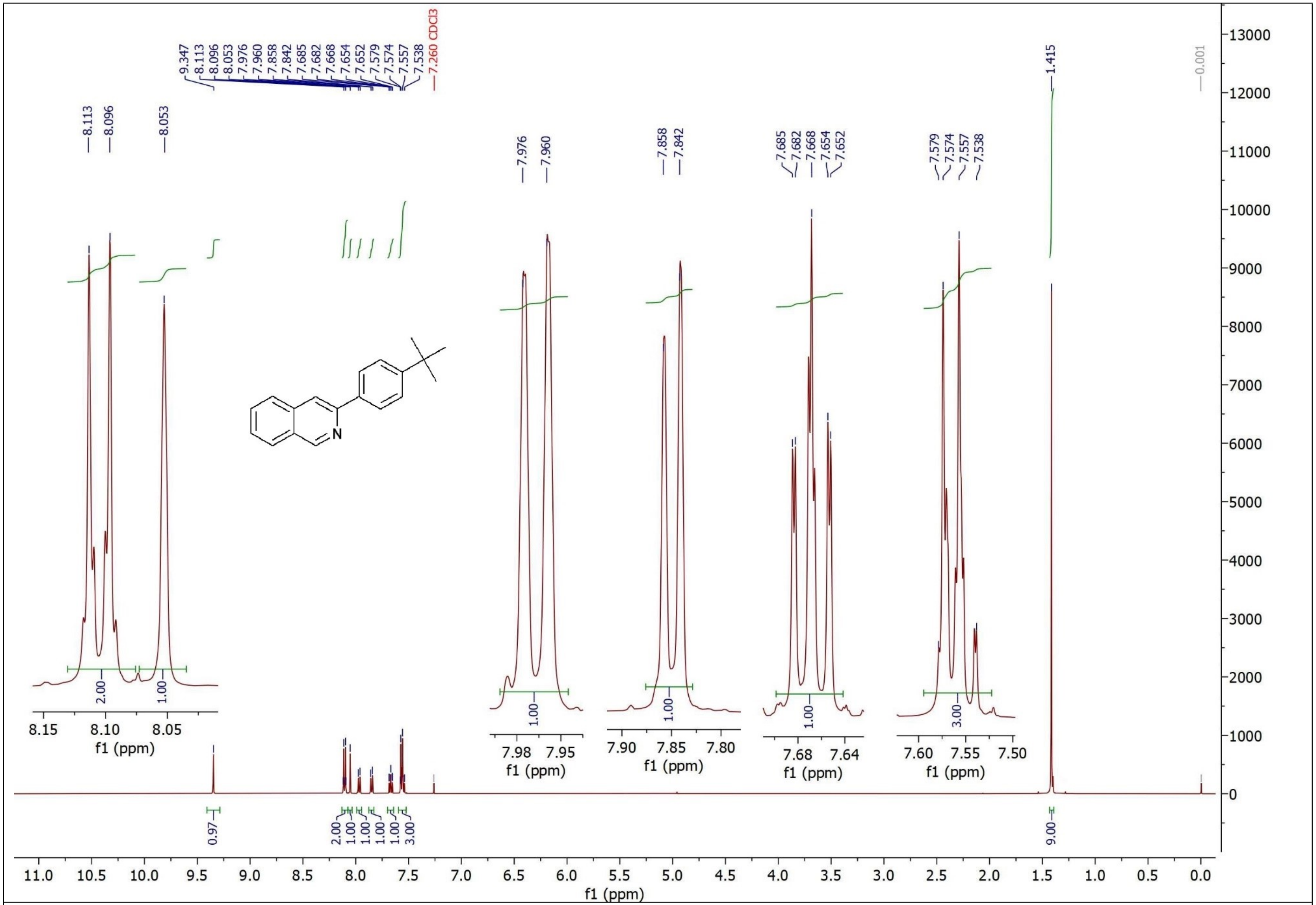

^{1}H NMR (500 MHz, $CDCl_3$): **3-(4-(*tert*-Butyl)phenyl)isoquinoline (3l)**

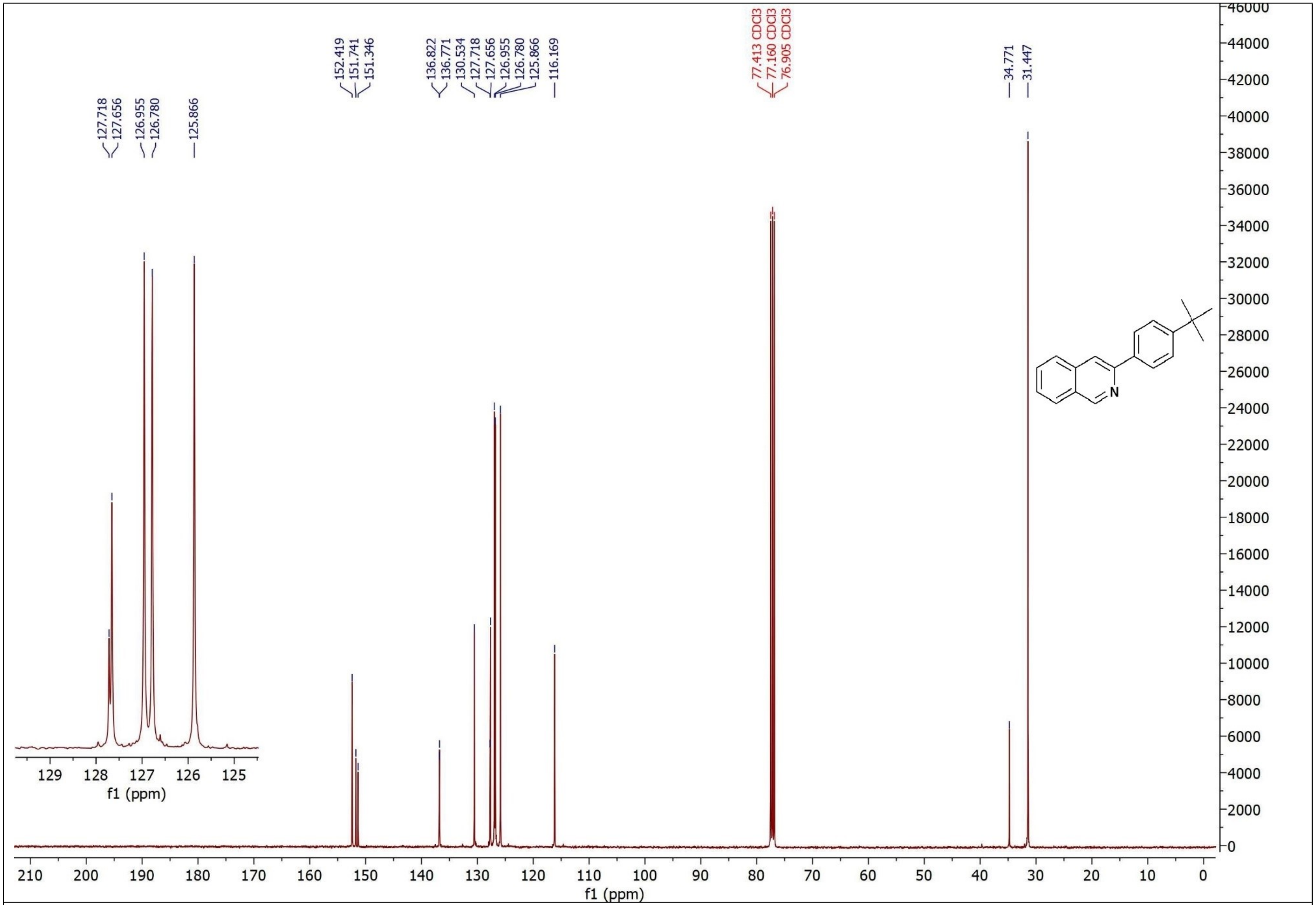

^{13}C NMR (125 MHz, $CDCl_3$): **3-(4-(*tert*-Butyl)phenyl)isoquinoline (3l)**

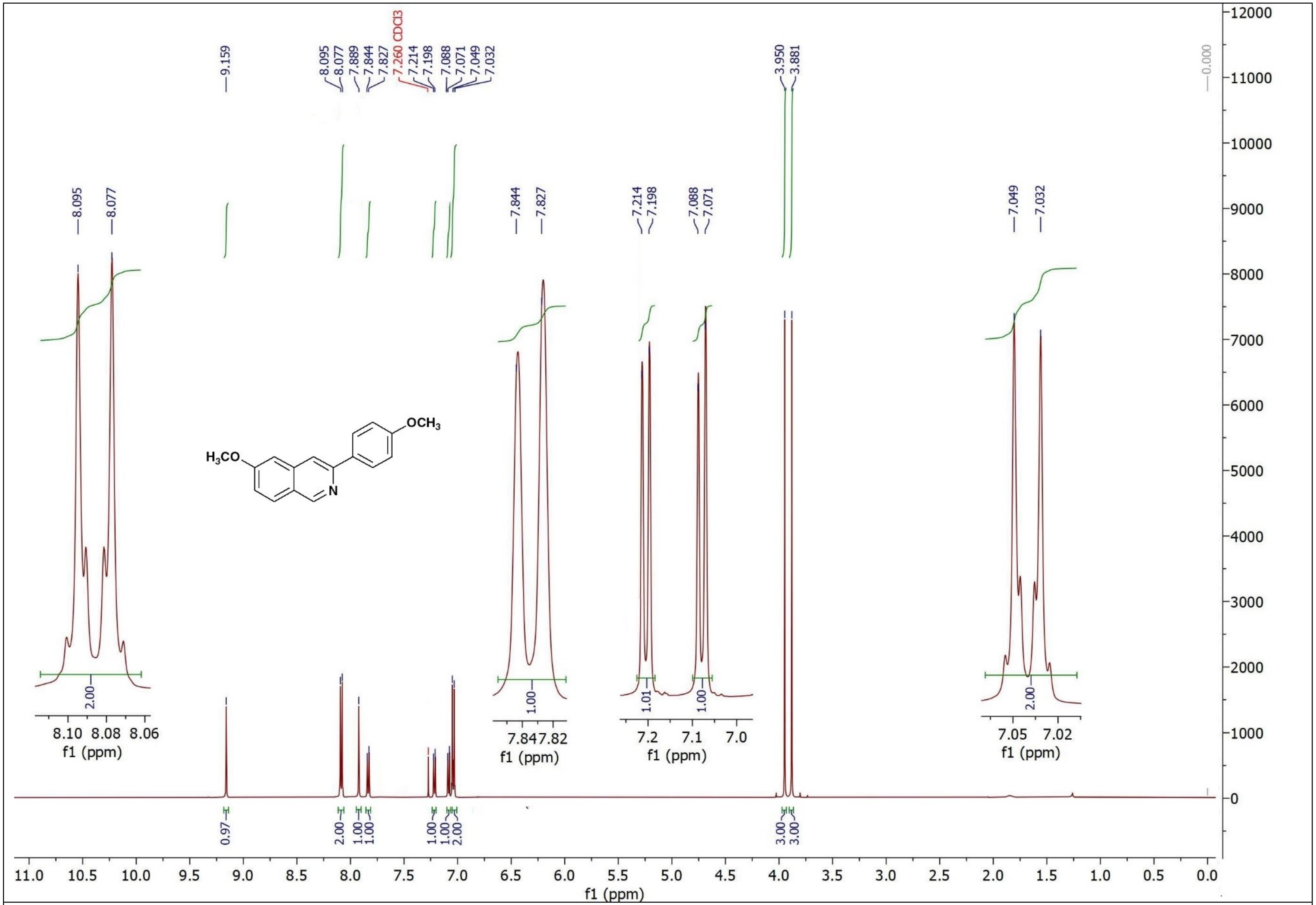

^1H NMR (500 MHz, $CDCl_3$): **6-Methoxy-3-(4-methoxyphenyl)isoquinoline (3m)**

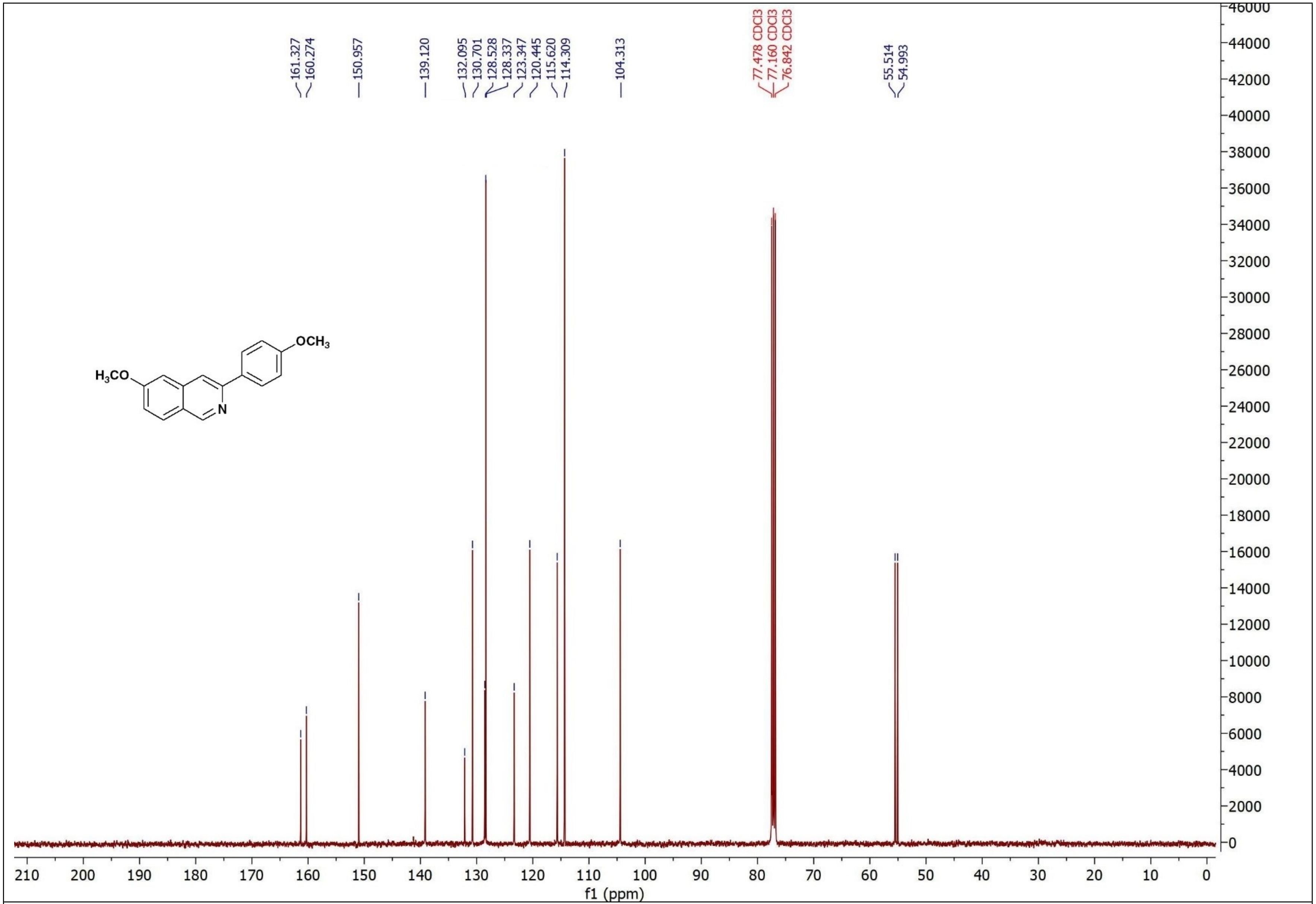

^{13}C NMR (125 MHz, $CDCl_3$): **6-Methoxy-3-(4-methoxyphenyl)isoquinoline (3m)**

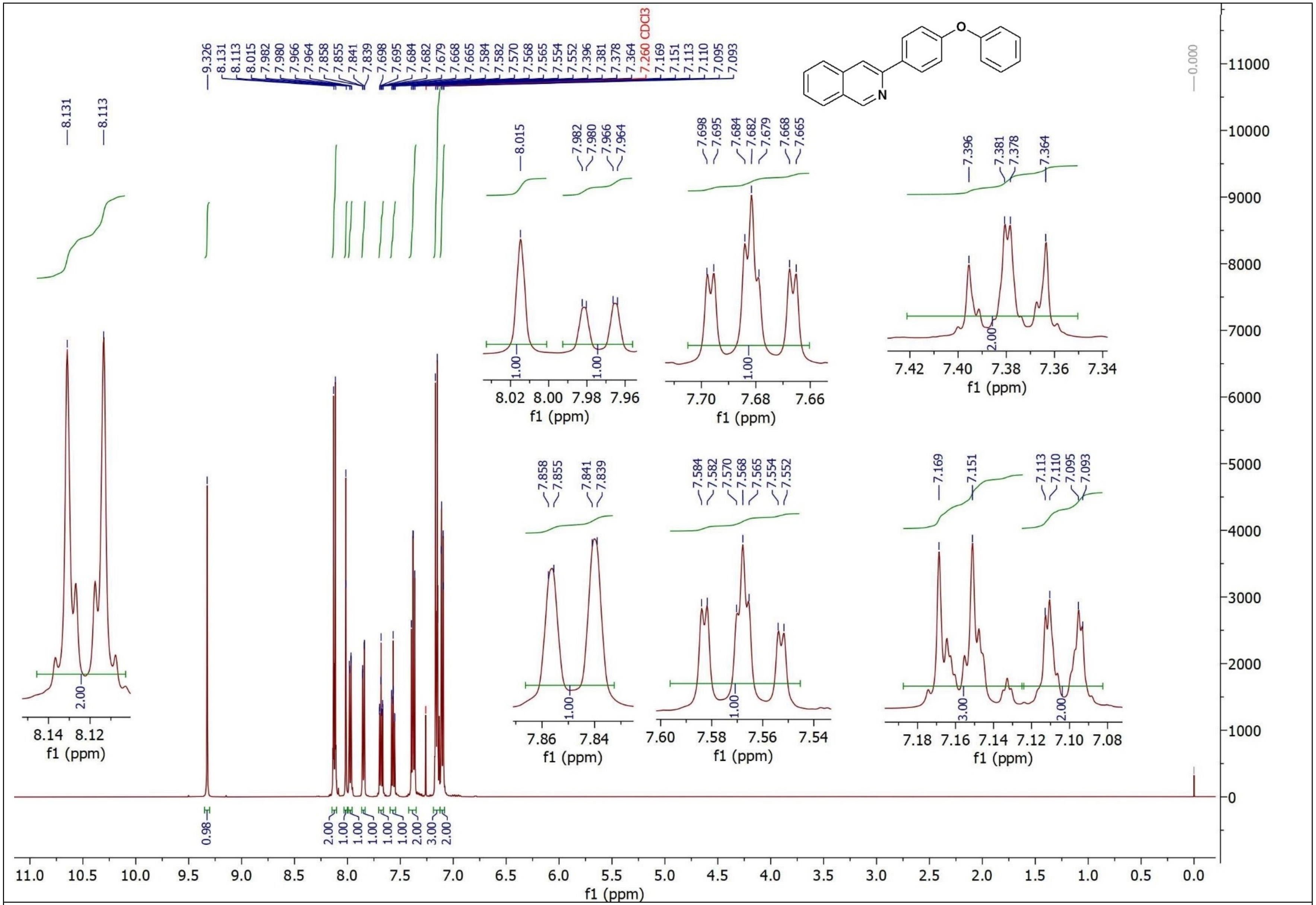

^{1}H NMR (500 MHz, $CDCl_3$): **3-(4-Phenoxyphenyl)isoquinoline (3p)**

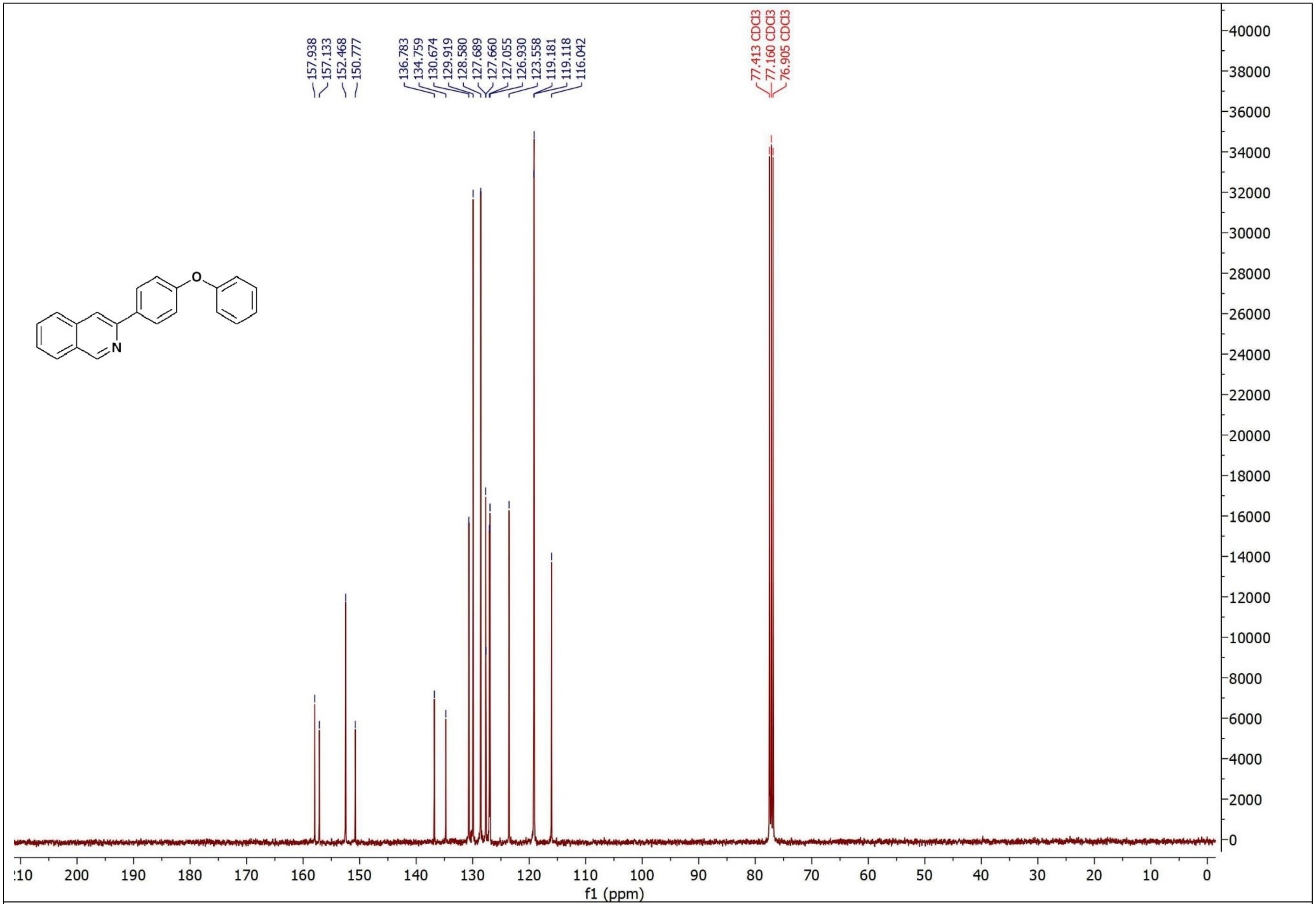

^{13}C NMR (125 MHz, $CDCl_3$): **3-(4-Phenoxyphenyl)isoquinoline (3p)**

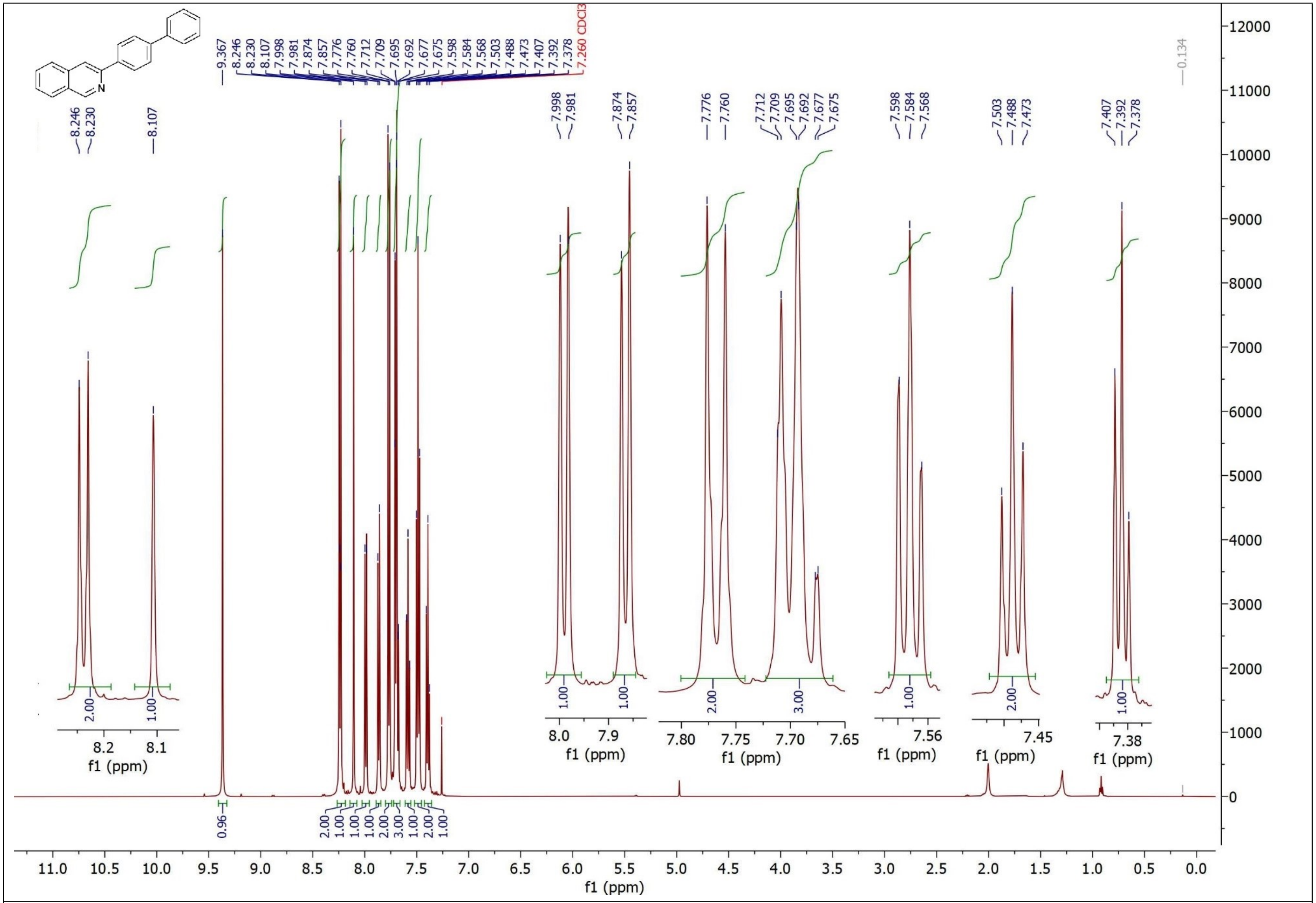

^{1}H NMR (500 MHz, $CDCl_3$): **3-([1,1'-Biphenyl]-4-yl)isoquinoline (3o)**

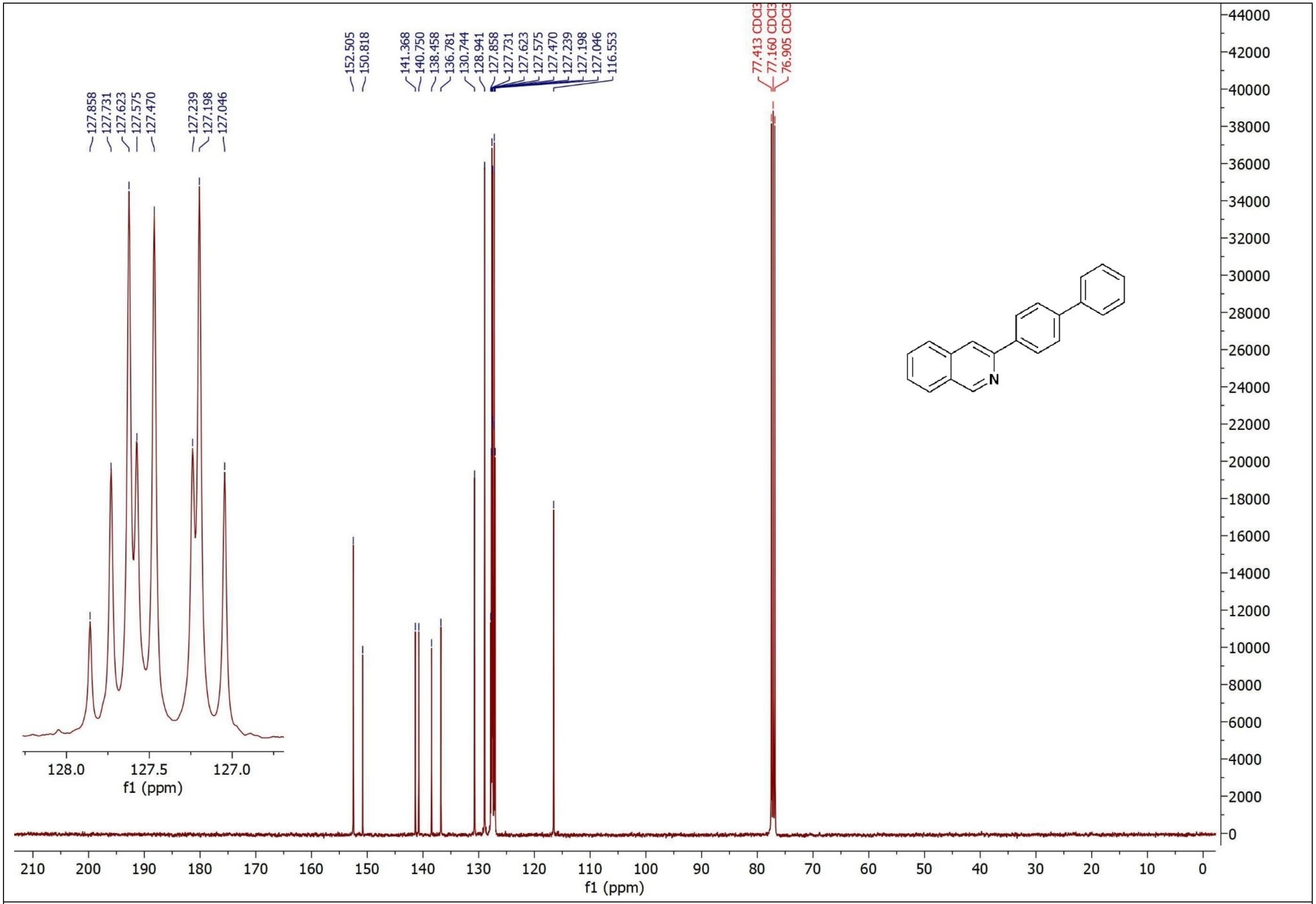

^{13}C NMR (125 MHz, $CDCl_3$): **3-([1,1'-Biphenyl]-4-yl)isoquinoline (3o)**

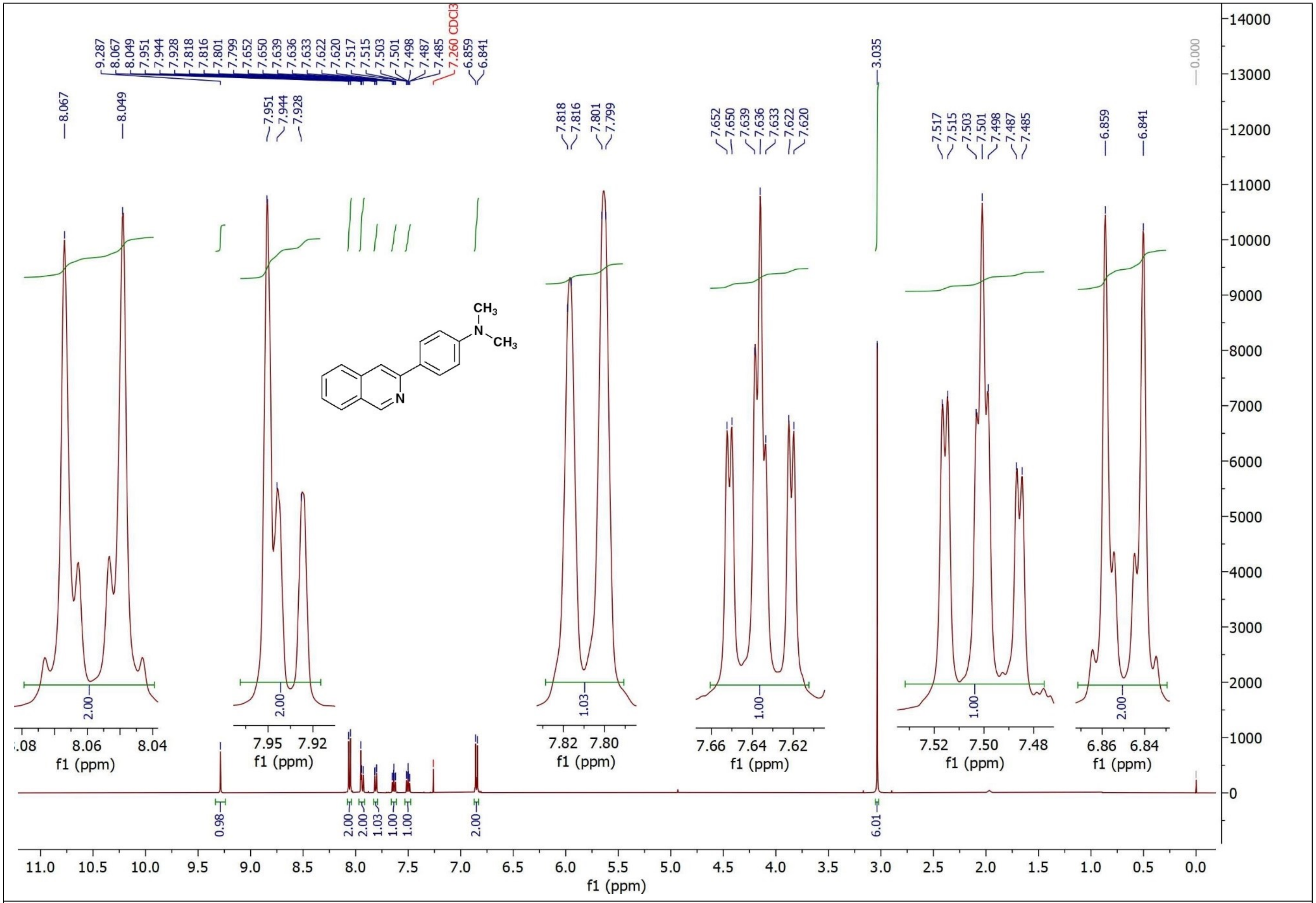

^{1}H NMR (500 MHz, $CDCl_3$): **4-(Isoquinolin-3-yl)-*N*,*N*-dimethylaniline (3p)**

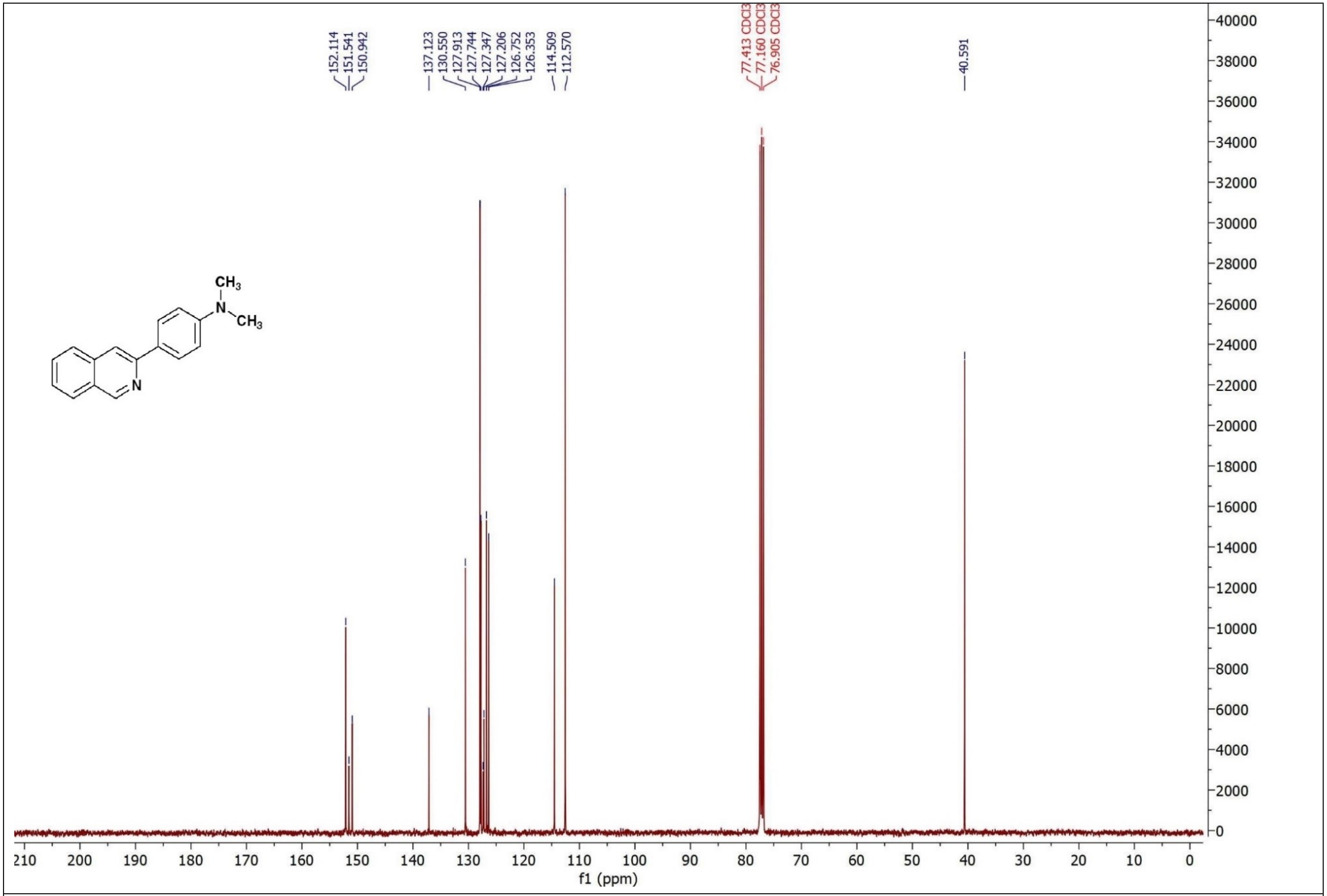

^{13}C NMR (125 MHz, $CDCl_3$): **4-(Isoquinolin-3-yl)-*N*,*N*-dimethylaniline (3p)**
